# Evaluating and Improving Automatic Sleep Spindle Detection by Using Multi-Objective Evolutionary Algorithms

**DOI:** 10.3389/fnhum.2017.00261

**Published:** 2017-05-18

**Authors:** Min-Yin Liu, Adam Huang, Norden E. Huang

**Affiliations:** ^1^Department of Biomedical Sciences and Engineering, Institute of Systems Biology and Bioinformatics, National Central UniversityTaoyuan, Taiwan; ^2^Research Center for Adaptive Data Analysis, National Central UniversityTaoyuan, Taiwan

**Keywords:** sleep spindles, automatic detection, Hilbert-Huang transform, performance assessment, multi-objective evolutionary algorithm, Pareto front

## Abstract

Sleep spindles are brief bursts of brain activity in the sigma frequency range (11–16 Hz) measured by electroencephalography (EEG) mostly during non-rapid eye movement (NREM) stage 2 sleep. These oscillations are of great biological and clinical interests because they potentially play an important role in identifying and characterizing the processes of various neurological disorders. Conventionally, sleep spindles are identified by expert sleep clinicians via visual inspection of EEG signals. The process is laborious and the results are inconsistent among different experts. To resolve the problem, numerous computerized methods have been developed to automate the process of sleep spindle identification. Still, the performance of these automated sleep spindle detection methods varies inconsistently from study to study. There are two reasons: (1) the lack of common benchmark databases, and (2) the lack of commonly accepted evaluation metrics. In this study, we focus on tackling the second problem by proposing to evaluate the performance of a spindle detector in a multi-objective optimization context and hypothesize that using the resultant Pareto fronts for deriving evaluation metrics will improve automatic sleep spindle detection. We use a popular multi-objective evolutionary algorithm (MOEA), the Strength Pareto Evolutionary Algorithm (SPEA2), to optimize six existing frequency-based sleep spindle detection algorithms. They include three Fourier, one continuous wavelet transform (CWT), and two Hilbert-Huang transform (HHT) based algorithms. We also explore three hybrid approaches. Trained and tested on open-access DREAMS and MASS databases, two new hybrid methods of combining Fourier with HHT algorithms show significant performance improvement with F_1_-scores of 0.726–0.737.

## Introduction

Sleep spindles are brief (at least 0.5 s), distinct bursts of brain activity in the sigma frequency range (11–16 Hz) as measured by electroencephalography (EEG). They are characterized by the waxing and waning shape of a spindle. Along with K-complexes they are key EEG features used to define non-rapid eye movement (NREM) stage 2 sleep in sleep scoring according to AASM (Iber et al., 2007) guidelines. These oscillations are also of great biological and clinical interests because they potentially play an important role in identifying and characterizing the processes of aging, learning, memory consolidation, as well as various neurological disorders. For example, spindle density (events per minute), amplitude, and duration decrease with age (Crowley et al., 2002; Martin et al., 2013). Recent evidence also suggests that spindle density, frequency, and activity have been correlated with both intelligence and general mental ability (Bódizs et al., 2005; Fogel et al., 2007; Schabus et al., 2008; Geiger et al., 2011; Gruber et al., 2013). In addition, increased sleep spindle density following learning improves memory consolidation (Eschenko et al., 2006; Tamminen et al., 2010; Bergmann et al., 2012). On the other hand, the sleep spindle deficiency in schizophrenia subjects may reflect dysfunction in thalamic-reticular and thalamocortical mechanisms (Ferrarelli et al., 2007). Some sleep spindle abnormalities implicating thalamocortical network dysfunction are also observed in schizophrenia (Wamsley et al., 2012). Furthermore, sleep spindle alterations are associated with later development of dementia in Parkinson's disease, and thus may serve as an additional biomarker of cognitive decline in these patients (Latreille et al., 2015). For the aforementioned reasons, detecting sleep spindles, and scoring their properties have become an important task in both research and clinical settings.

Sleep spindles are conventionally identified through visual inspection of the EEG data by expert sleep clinicians. Although such practice is the gold standard for spindle detection, it is a laborious, subjective process, and the results are rather inconsistent among different experts (O'Reilly and Nielsen, 2015). Because of the rapidly growing biological and clinical interests in sleep spindles, many automated detection methods of sleep spindles have been developed to improve the process. There are several basic methodological strategies for automating spindle detection, each of which has given rise to many closely related spindle detectors. One of the first automated sleep spindle detectors based on a bandpass filtering and amplitude thresholding approach was published by Schimicek et al. (1994). Thereafter, Fourier-based bandpass filtering has become the foundation of numerous new algorithms for frequency-based discrimination (Mölle et al., 2002; Ferrarelli et al., 2007; Huupponen et al., 2007; Bódizs et al., 2009; Wendt et al., 2012; Martin et al., 2013). Some algorithms replace the bandpass filtering with wavelet transformation (Sitnikova et al., 2009; Wamsley et al., 2012; Adamczyk et al., 2015; Lajnef et al., 2015; Tsanas and Clifford, 2015). Alternatively, Causa et al. (2010) propose using Hilbert-Huang transform (HHT) for determining sleep spindle's instantaneous frequency and amplitude (Huang et al., 1998). Although there are more and more open-access automated sleep spindle detectors becoming available in the literature (O'Reilly, 2013; Warby et al., 2014), the performance of these open-access spindle detectors remains equally inconsistent from study to study because of: (1) the lack of common benchmark databases, and (2) the lack of commonly accepted evaluation metrics. The first problem has been addressed recently and in response, there are two publicly available databases: the DREAMS database (Devuyst, 2013) and the Montreal Archive of Sleep Study (MASS) database (O'Reilly et al., 2014). The second problem has also received quite a lot of attention and resulted in several fruitful papers in the literature (Huupponen et al., 2007; Devuyst et al., 2011; Warby et al., 2014; O'Reilly and Nielsen, 2015). However, we think that the second problem remains ambivalent as the gold standard may vary greatly from expert to expert (or institute to institute) even for the same dataset (Tables 1, 2; Devuyst et al., 2011; Warby et al., 2014; O'Reilly and Nielsen, 2015). To resolve this dilemma, we suggest that commonly acceptable evaluation metrics should be gold standard adaptive. This adaptive capability is crucial because abnormal spindles in general play a more important role in a real clinical setting. Therefore, an ideal detector should excel in the ability to find the clinically significant sleep spindles specified by a gold standard of the user's choice.

**Table 1:** **Summary of sleep spindle numbers in DREAMS database with 4 different gold standards**.

**GS\Subj**	**Subj 1**	**Subj 2**	**Subj 3**	**Subj 4**	**Subj 5**	**Subj 6**	**Subj 7**	**Subj 8**	**Total**
Scorer 1	52	60	5	44	56	72	18	48	355
Scorer 2	115	52	44	25	86	87	–	–	409
Intersection	33	35	5	6	39	42	–	–	160
Union	134	77	44	63	103	117	18	48	604

*GS stands for gold standard*.

**Table 2:** **Summary of MASS SS2 database with gold standards by Scorers 1 and 2, and their overlapping information by intersection and union operations**.

**Subj\GS**	**Scorer 1**	**Scorer 2**	**Intersection**	**Union**
Subj 1	1,040	2,389	1,025	2,404
Subj 2	1,142	2,191	1,120	2,212
Subj 3	143	596	134	605
Subj 4	250	–	–	–
Subj 5	341	1,186	331	1,194
Subj 6	150	829	139	838
Subj 7	905	1,572	820	1,655
Subj 8	384	–	–	–
Subj 9	810	1,643	781	1,671
Subj 10	790	1,909	769	1,930
Subj 11	605	1,521	594	1,529
Subj 12	705	1,188	653	1,236
Subj 13	692	1,427	658	1,458
Subj 14	708	1,601	681	1,626
Subj 15	97	–	–	–
Subj 16	445	–	–	–
Subj 17	469	1,189	453	1,205
Subj 18	1,156	1,662	1,045	1,773
Subj 19	315	1,048	311	1,052
Total	11,147	21,951	9,514	22,388

**Figure 1:**
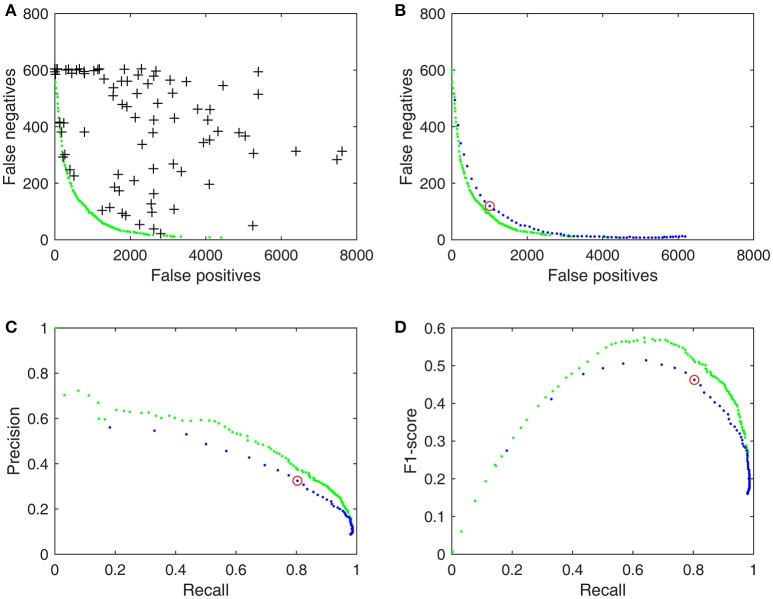
**Pareto fronts and proposed performance metrics for detector d_3_, trained results by using DREAMS database with the union gold standard (Table 1)**. **(A)** 100 randomly chosen operating parameter values (crosses) and the Pareto front (green dots) solved by SPEA2. **(B)** The performance of d_3_ with a single set of values (red circle) and with an adjustable threshold (blue dots). **(C)** PR-curve as performance metrics derived from FN and FP in **(B)**. **(D)** Derived *F*_1_-curve.

In this paper, we have focused on tackling the second problem by proposing to evaluate the performance of a spindle detector in a multi-objective optimization context with the resultant Pareto fronts as the basis for deriving more commonly accepted performance evaluation metrics such as precision (P), recall (R), and F_1_-scores. In a nutshell, the performance of any type of detector can be characterized by two competing objectives: low false negative (FN) and low false positive (FP) rates (Huang et al., 2010). As a sleep spindle detector generally has several operating parameters such as upper/lower frequency, amplitude, and duration criteria, these parameters can be adjusted and optimized according to a given training dataset with a specific gold standard. Among all possible combinations of operating parameter values, the commonly accepted optimal solutions of such a multi-objective problem are a set of Pareto optimal solutions known as the Pareto front in objective space (Figure 1A; Knowles and Corne, 2000; Zitzler et al., 2001a; Messac et al., 2003). Although no close form solutions are available for most spindle detectors, these types of optimization problems can be solved by using multi-objective evolutionary algorithms (MOEA). MOEA is a mature technique that is applied in many fields (Doncieux et al., 2011) and efficient MOEAs now exist, as for instance NSGA-II (Deb et al., 2002) and ε-MOEA (Deb et al., 2005). Compared with any empirical chosen operating point shown in Figure 1, the Pareto optimal solutions of FN and FP and their derived PR- and F_1_-curves are much more informative in evaluating and comparing automatic spindle detectors. We hypothesize that the Pareto optimal operating parameters solved by using MOEA will improve automatic sleep spindle detection. The rationale and derivation are given in the Methods section.

Here we used the Strength Pareto Evolutionary Algorithm (SPEA2) (Zitzler et al., 2001a,b), another popular MOEA, to optimize six existing frequency-based sleep spindle detection algorithms. They included three Fourier (Mölle et al., 2002; Ferrarelli et al., 2007; Martin et al., 2013), one continuous wavelet transform (CWT) (Tsanas and Clifford, 2015), and two Hilbert-Huang transform (HHT) based algorithms (Causa et al., 2010; Huang et al., 2015). We also explored three hybrid approaches in combining Fourier- or CWT- with HHT-based methods for improvement. The experiment was conducted by using both hold-out and cross-validation strategies such that hold-out was used to assess overfitting by our SPEA2 implementations and cross-validation was used to assess spindle detectors. The sleep spindle data and experimental methods are delineated in the following section.

## Materials and methods

### Data

Two publicly available databases were used to evaluate 6 simplex and 3 hybrid automated sleep spindle detection algorithms. The first sleep EEG database was from the DREAMS Spindles Database of University of MONS—TCTS Laboratory and Université Libre de Bruxelles—CHU de Charleroi Sleep Laboratory (Devuyst, 2013). It consists of eight patients (4 men and 4 women aged between 31 and 53) with different pathologies (Devuyst et al., 2011). A segment of 30 min of the central EEG channel (C3-A1 or Cz-A1) from these 8 individual patients are publicly available on the DREAMS Database website where the sampling frequency is 200 Hz (6 patients), 100 Hz (1 patient), and 50 Hz (1 patient) respectively. Sleep spindles of these 30-min-long EEG signals are annotated independently by two experts. The second expert annotated only six out of eight datasets and the spindles are uniformly assigned 1-s-long duration. In order to build a common ground truth from multiple raters, there are several different approaches such as treating individual rater separately (O'Reilly and Nielsen, 2015), the total agreement (intersection operation; Devuyst et al., 2011), and partial agreement (union operation; Warby et al., 2014; Tsanas and Clifford, 2015). For forming (total/partial) agreement sets, we took the same approach proposed by Tsanas and Clifford (2015) specifically for the DREAMS database. In the case of overlapped spindles, we only kept the annotations by expert 1 for their better duration assessment. In total, the number of identified spindles was 355 (from 8 subjects) by scorer 1, 409 (from 6 subjects) by scorer 2, 160 by both, and 604 by either, respectively. The details are summarized in Table 1. Note that the authors of the DREAMS database did not specify which scoring rules the experts used for scoring spindles.

The second EEG sleep database was a subset (denoted as SS2) of the MASS database. The MASS database consists of 200 polysomnographic (PSG) recordings gathered from eight research protocols conducted between 2001 and 2013 in three different laboratories at the Center for Advanced Research in Sleep Medicine (CARSM), Montreal, Canada (O'Reilly et al., 2014). The MASS SS2 consists of 19 complete-night PSG recordings sampled at 256 Hz from young healthy subjects. For this subset, sleep spindles are scored by two experts on NREM stage 2 sleep epochs and on channel C3 with linked-ear reference. The first scorer used traditional AASM scoring rules. The second scorer used both broad-band EEG signals (0.35–35 Hz band) and sigma filtered signals (11–17 Hz band) to facilitate the identification of short duration, small amplitude or obscured (by delta waves or K-complexes) spindles. Also, no minimal spindle duration was used by the second scorer and four datasets (out of the 19) were not scored due to poor quality sleep or signal (O'Reilly and Nielsen, 2015). In total, the number of identified spindles was 11,147 (from 19 subjects) by scorer 1, 21,951 (from 15 subjects) by scorer 2, 9,514 by both, and 22,388 by either, respectively. The details are summarized in Table 2. Note that the total/partial (intersection/union) agreement in Table 2 is solely given as supplementary information to describe inter-rater agreement. The intersection and union operations were carried out based on each sample point (by-sample) and then the resultant spindle points were regrouped into individual sleep spindles (by-event) without duration checking.

### Performance evaluation

The performance of a diagnostic test is generally characterized by sensitivity and specificity. However, sleep spindles are sparse events such that their lengths sum up to 8.2 (mean) ± 4.9 (standard deviation) and 29.4 ± 11.2 min per whole night sleep according to scorers 1's and 2's annotations of the MASS SS2 database respectively. The performance evaluation using specificity measurement will be high and therefore provide unrealistically positive results. To avoid this pitfall, Warby et al. (2014) propose using precision, recall, and F_1_-score for the evaluation of infrequent, discrete events such as sleep spindles in the EEG signal. Let *TP* denote the amount of true positives, *FN* false negatives and *FP* false positives, precision, *P*, and recall, *R*, are defined as:

(1) $$P=\frac{TP}{TP+FP}$$

(2) $$R=\frac{TP}{TP+FN}$$

Taking the weighted harmonic average of precision and recall leads to the *F*-score,

(3) $$F_{\beta }=\left(1+\beta^{2}\right)\frac{PR}{R+\beta^{2}P}=\frac{\left(1+\beta^{2}\right)TP}{\left(1+\beta^{2}\right)TP+\beta^{2}FN+FP}$$

If we assume a uniform prior (β = 1), then

(4) $$F_{1}=\frac{2PR}{P+R}.$$

Although *P, R*, and *F*_1_ are commonly used evaluation metrics for the assessment of spindle detector performance, so far their potentials have not been fully explored yet. In the original work of Warby et al. (2014), spindle detectors were only tested with default operating parameters. This view is too narrow as a detector generally has several adjustable operating parameters that allow the user to optimize its performance between two competing objectives: minimizing *FN* and minimizing *FP*. For example, we can take the root mean square (RMS) algorithm of Martin et al. (2013) and test it out on the DREAMS database, the default operating parameter with a threshold of 0.95 (95 percentile) and a duration between 0.3 and 3 s. The result is simply a point (red circle) among many other possible performances (black crosses) on the objective space as shown in Figures 1A,B. O'Reilly and Nielsen (2015) took a step forward by making the threshold an adjustable parameter within a range between 0.7 and 0.995. The resultant *FN*-*FP* pairs, *PR*-curve and *F*_1_-curve (blue dots in Figures 1B–D) provide a broader view to evaluate the detector's performance. In this study, we extended the idea of O'Reilly and Nielsen (2015) even further by making adjustable more operating parameters (such as the upper and lower duration criteria) and derived the performance metrics from the Pareto optimal solutions (green dots in Figure 1) in the multi-objective optimization context. Figure 1 demonstrates that results from a detector can be improved substantially in a multi-objective context by allowing the operating parameters to adapt to a training gold standard and that its Pareto optimal solutions enable us to define some useful performance metrics uniquely. The following section summarizes the proposed performance metrics more formally.

### Pareto front-derived performance metrics

In a multi-objective context, the performance of a sleep spindle detector is described by a pair of raw numbers: *FN* and *FP*. The number of detections on the C3 channel of EEG signals that do not have an appropriate overlap rate (*R*_ov_) with any true sleep spindle (SS) is defined as *FP*; similarly, the number of true sleep spindles that are not detected by automatic detectors is defined as *FN*. Note that *FN* and *FP* were defined in the event-by-event analysis context (Warby et al., 2014) throughout this study. Note also that a true positive event-detection (D) was scored based on an overlap rate,

(5) $$R_{\text{ov}}=\frac{\text{SS}\cap \text{D}}{\text{SS}\cup \text{D}}>0.2.$$

We followed these scoring rules to make our results comparable to the work by Warby et al. (2014) because the first three Fourier-based detectors evaluated in this study were derived directly from their work.

In mathematical terms, let *FN* and *FP* be described as functions of an operating parameter set **x**: *FP*(**x**) and *FN*(**x**). For example, **x** includes threshold, lower duration, and upper duration for the RMS algorithm illustrated in Figure 1. Figure 1A shows a random selection **x**'s of 100 possible operating parameter combinations with their results [*FN*(**x**), *FP*(**x**)] scattered around the objective space. The optimal solutions of such a detector involve minimizing both *FN* and *FP* rates simultaneously. Since low *FN* and *FP* rates are two conflicting objectives, this problem does not produce a single optimal solution but a set of possible solutions known as a Pareto optimal set, which results in a Pareto front (green dots in Figure 1A) in objective space. A Pareto front is essentially an objective boundary such that any solution on the front can only be outperformed by another solution in at most one of the two competing objectives. Therefore, a Pareto optimal set is also called a Pareto non-dominated set.

Formally the multi-objective optimization problem can be equivalently stated as minimizing a two-objective vector

(6) F(x)=(FN(x),FP(x))

where **x** is the vector of a detector's operating parameters. A solution **x**_1_ is said to dominate **x**_2_ if and only if

(7) $$\begin{array}{l} FN\left(x_{1}\right)\;\leq \;FN\left(x_{2}\right)\;\;\text{and}\\ FP\left(x_{1}\right)\;\leq FP\left(x_{2}\right)\;\text{ and}\\ \{FN\left(x_{1}\right)<FN\left(x_{2}\right)\;\text{ or}\;FP\left(x_{1}\right)<FP\left(x_{2}\right)\} \end{array}$$

where **x**_2_ ≠ **x**_1_. From the aforementioned Pareto optimal solutions and Equations (1–4), we are able to derive PR-curves and *F*_1_-curves that are Pareto optimal as illustrated in Figures 1C,D.

The solutions of Equation (6) satisfying the conditions listed in Equation (7) can be found rather efficiently by MOEA with genetic mechanisms. In a standard genetic algorithm, there are usually four steps in the evolutionary procedure: (1) randomly initializing the solution population, (2) evaluating and assigning a fitness value for each individual in the population according to its performance, (3) selecting individuals based on their fitness values to procreate, and (4) using crossover and mutation to produce next generation from the selected individuals. In this study, we used the SPEA2 algorithm to find the Pareto fronts of all of the 9 examined detectors. SPEA2 was selected for its fast convergence rate and good performance because it kept a relatively small, yet diverse population (Zitzler et al., 2001a).

**Figure 2:**
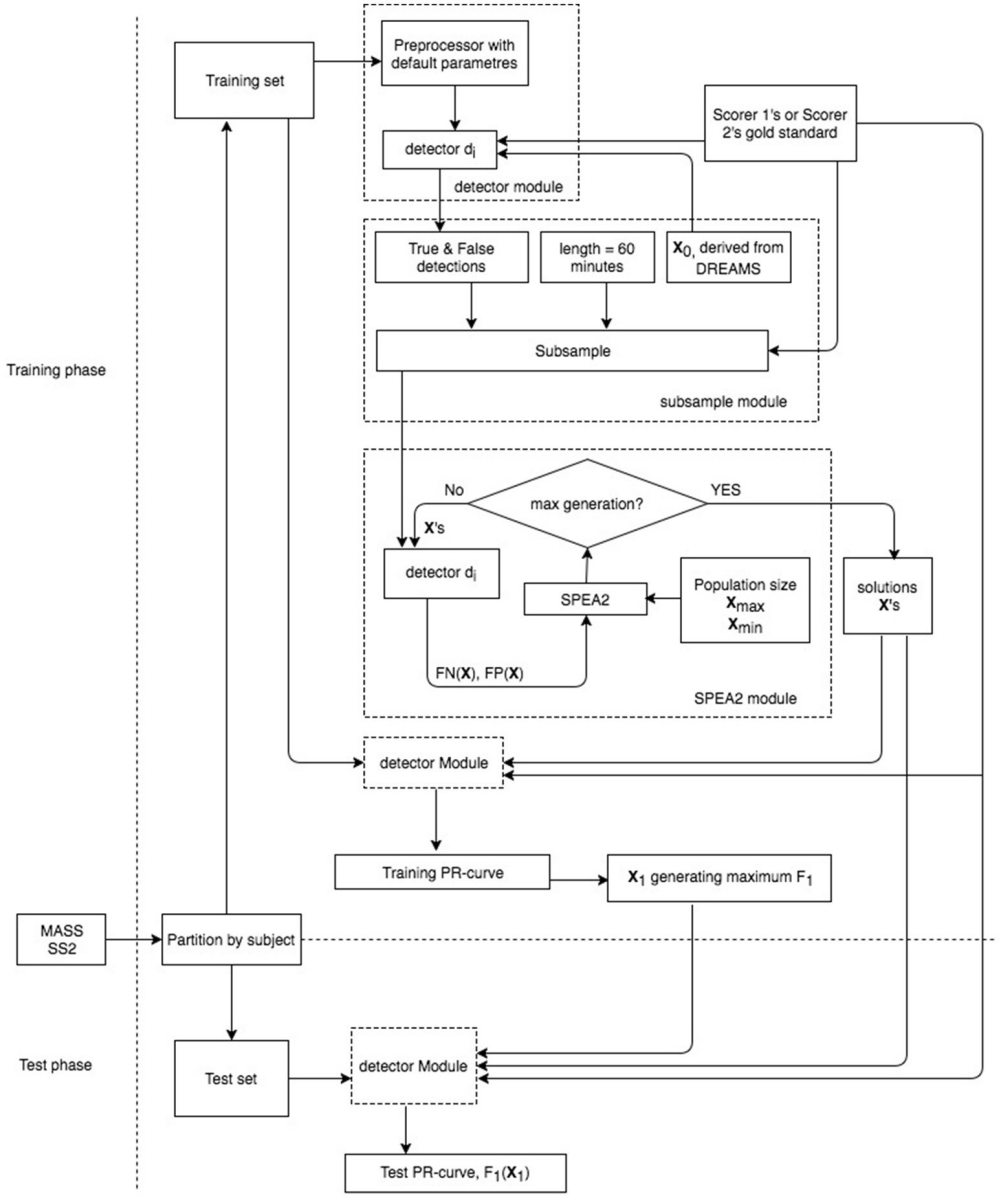
**Work flowchart of proposed evaluation metrics**.

A brief work flowchart of our proposed metrics is provided in Figure 2. It consists of three key modules: (1) detector, (2) subsample, and (3) SPEA2 modules. Six simplex and 3 hybrid detectors with their operating parameter descriptions are given in Sections Six Simplex Detectors and Three Hybridization Detectors respectively. Subsample and SPEA2 implementations are delineated in Sections Subsample Strategy and SPEA2 Module.

### Six simplex detectors

**Figure 3:**
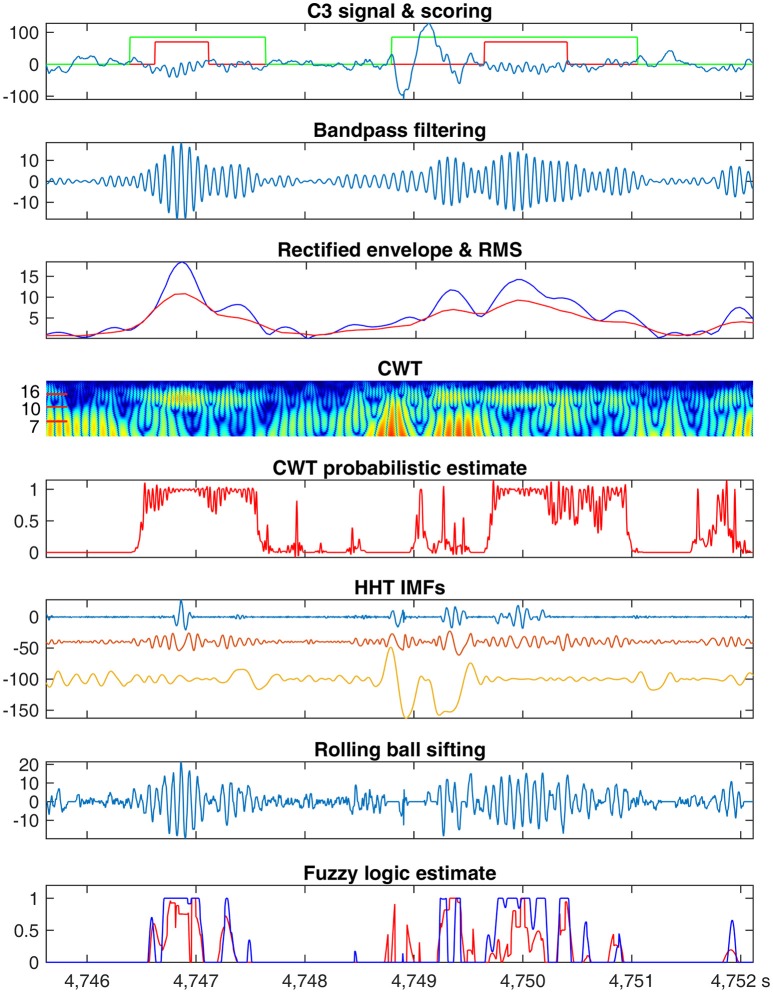
**Frequency-based sleep spindle detection**. An example C3 signal (data 1 from the MASS database) with sleep spindles marked by scorer 1 in red and scorer 2 in green (1st row). Bandpass filtering results (2nd row). Envelope of rectified filtered signal peaks (blue) and RMS (red) of filtered results (3rd row). Normalized power spectrum of CWT coefficients (4th row). CWT probabilistic estimate derived from top 10 coefficients (5th row). First 3 IMFs by the EMD method (6th row). High frequency component extracted by the rolling ball sifting algorithm with a cutoff frequency at 10 Hz (7th row). Fuzzy logic estimates of d_5_ (red) and d_6_ (blue) (8th row).

Fourier-based bandpass filtering (Figure 3, 2nd row) is the foundation of many automated detection methods for identifying the frequency of sleep spindles. The main difference among such bandpass automatic spindle detectors is to apply various methodological strategies for improving the identification of the “right amplitude” and “right duration” of sleep spindles. Alternatively, CWT and HHT for frequency discrimination are also proposed in more recent approaches. We have focused on six existing algorithms: three using Fourier filtering, one using CWT, and two using HHT. They are briefly reviewed as follows.

#### Detector 1. (d_1_). Ferrarelli's bandpass filtering and amplitude thresholding

The first detector d_1_, proposed by Ferrarelli et al. (2007), detects sleep spindles through bandpass filtering and using lower and upper amplitude thresholds. In our Pareto-optimization software implementation (refer to Supplementary Material for all software implementation), the EEG signal was preprocessed with a filter using the bandpass setting published by Warby et al. (2014) and the envelope of the rectified filtered signal peaks (blue curve in Figure 3, 3rd row) was thresholded by two parameters: *p*_1,1_ × *A*_1_ and *p*_1,2_ × *A*_2_. *A*_1_ and *A*_2_ were derived respectively from the peak and average amplitude of filtered signals in NREM stages 2, 3, and 4; *p*_1,1_ and *p*_1,2_ were the lower and upper threshold ratios respectively. Finally, each spindle candidate was examined by the lower and upper duration criteria *p*_1,3_ and *p*_1,4_ s. As an optimization algorithm in our implementation, the vector of operating parameters for detector d_1_ can be explicitly expressed as

(8) $$x_{\text{d}1}=\left(p_{1,1},p_{1,2},p_{1,3},p_{1,4}\right).$$

#### Detector 2. (d_2_). Mölle's bandpass filtering and RMS thresholding

Mölle et al. (2002) published a detection method by first applying a bandpass filter to the EEG signal and then computing the RMS of the filtered signal. In our software implementation, the RMS of the filtered signal was calculated with a time resolution of *p*_2,1_ s and a window of *p*_2,2_ s. The lower amplitude threshold was defined as *p*_2,3_ (a threshold ratio) times the standard deviation of the filtered NREM stage 2 signals. Parameters *p*_2,4_ and *p*_2,5_ defined the lower and upper spindle durations. The vector of operating parameters for detector d_2_ was

(9) $$x_{\text{d}2}=\left(p_{2,1},p_{2,2},p_{2,3},p_{2,4},p_{2,5}\right).$$

#### Detector 3. (d_3_). Martin's bandpass filtering and RMS thresholding

Martin et al. (2013) published a detection method that also took the RMS approach (similar to d_2_) with different time resolution *p*_3,1_ = 0.25 s, time window *p*_3,2_ = 0.25 s, and a threshold ratio *p*_3,3_ as the 95 percentile (0.95) of the RMS amplitude of the bandpass filtered signal in NREM stages 2, 3, and 4. And *p*_3,4_ and *p*_3,5_ defined the lower and upper spindle durations. In this study, however, time resolution *p*_3,1_ and time window *p*_3,2_ were fixed at 0.1 and 0.25 s respectively so that RMS was preprocessed only once to reduce the high RMS-and-percentile computation cost for all NREM stages 2, 3, and 4. Note that *p*_3,1_ was lowered from 0.25 to 0.1 s for a finer time resolution. An example RMS is shown as the red curve in Figure 3, 3rd row. The final vector of operating parameters for detector d_3_ was

(10) $$x_{\text{d}3}=\left(p_{3,3},p_{3,4},p_{3,5}\right).$$

#### Detector 4. (d_4_). Tsanas' CWT instantaneous probabilistic estimate with moving averaging

Detector d_4_ is one of two CWT-based methods proposed by Tsanas and Clifford (2015). With a Morlet basis function which identifies regions where the power of CWT coefficients corresponding to frequencies of spindles, d_4_ has the advantage over the previous 3 Fourier-based methods without needing the sleep stage information for deriving a normalized threshold. However, it is also the most complicated detector with 15 adjustable parameters. In our implementation, we first made the lower and upper spindle frequencies adjustable parameters *p*_4,1_ and *p*_4,2_. The normalized percentage power of the CWT coefficients (Figure 3, 4th row) were sorted in descending order at each time instant and the instantaneous probabilistic estimate of spindle occurrence was derived from the top 10 scales which fell in the range between *p*_4,1_ and *p*_4,2_ Hz. Second, the probabilistic estimate was smoothed by a moving average filter of *p*_4,3_ s (Figure 3, 5th row**)**. Third, candidate spindles were detected by a probabilistic estimate threshold *p*_4,4_ and initial regions longer than *p*_4,5_ s were kept and merged with neighboring regions if their time gap was shorter than *p*_4,6_ s. The last step was to group together regions which contained series of samples with high probabilities of denoting spindles. There were two grouping rules. One was for grouping intermediate spindle candidates with a lower average probability threshold *p*_4,10_ and one candidate was at least *p*_4,8_ s long and the other was at least *p*_4,9_ s. Similarly, *p*_4,7_, *p*_4,11_, and *p*_4,12_ were for grouping strong spindle candidates with an higher average probability threshold *p*_4,7_ and a duration over *p*_4,11_ or *p*_4,12_ s. Finally, *p*_4,13_, *p*_4,14_, and *p*_4,15_ were the merging time gap, lower duration, and upper mergeable duration criteria, respectively. The vector of operating parameters for detector d_4_ was

(11) $$\begin{array}{l} x_{\text{d}4}=(p_{4,1},p_{4,2},p_{4,3},p_{4,4},p_{4,5},p_{4,6},p_{4,7},p_{4,8},p_{4,9},p_{4,10},p_{4,11},\\ \;p_{4,12},p_{4,13},p_{4,14},p_{4,15}) \end{array}$$

#### Detector 5. (d_5_). Causa's HHT instantaneous frequency and amplitude fuzzy-logic estimate

Causa et al. (2010) proposed using HHT-derived instantaneous amplitude and frequency for determining sleep spindle's probabilistic estimate. HHT is fundamentally different from Fourier- or CWT-based approaches because it can generate physically meaningful components, called intrinsic mode functions (IMFs), empirically through a sifting procedure which fits extrema with splines recursively (Huang et al., 1998). In our software implementation, we generated first three IMFs (Figure 3, 6th row) and used a zero crossing method (Huang et al., 2009) for estimating their instantaneous frequency. For deriving probabilistic estimates, 4 positive parameters *p*_5,1_, *p*_5,2_, *p*_5,3_, and *p*_5,4_ μV were used to form the trapezoidal-shaped membership function (*p*_5,2−_ *p*_5,1_, *p*_5,2_, *p*_5,2+_ *p*_5,3_, *p*_5,2+_ *p*_5,3+_ *p*_5,4_) for amplitude. Similarly, 4 parameters *p*_5,5_, *p*_5,6_, *p*_5,7_, and *p*_5,8_ Hz for frequency. The fuzzy-logic estimates from amplitude and frequency were multiplied for each IMF respectively, and the maximal estimate of all 3 IMFs at each sample time was retained as the final fuzzy-logic estimate (red curve in Figure 3, 8th row). Lastly, the final estimate was thresholded by *p*_5,9_, merged by a time gap criterion *p*_5,10_ s, and checked by lower and upper duration criteria *p*_5,11_ and *p*_5,12_ s. The vector of operating parameters for d_5_ was

(12) $$\begin{array}{l} x_{\text{d}5}=(p_{5,1},p_{5,2},p_{5,3},p_{5,4},p_{5,5},p_{5,6},p_{5,7},p_{5,8},p_{5,9},p_{5,10},\\ \;p_{5,11},p_{5,12}) \end{array}$$

#### Detector 6. (d_6_). Huang's rolling ball sifting frequency and amplitude fuzzy-logic estimate

Huang et al. (2015) proposed a new HHT-based detector that applied a bandpass empirical mode decomposition (EMD) algorithm to extract IMFs with an adjustable frequency discriminating capability. It worked in a way similar to d_5_ except that the IMF containing sleep spindles was extracted by using two rolling balls (with cutoff frequencies at 10 and 16 Hz respectively) for selecting appropriate extrema in the sifting process. Since the rolling ball algorithm was computationally expensive, instead of using two balls, we applied a new rolling ball sifting algorithm (Huang et al., 2016) with only one ball (cutoff frequency at 10 Hz) in our new software implementation for processing long MASS datasets. The extracted high frequency component (>10 Hz), illustrated in Figure 3, 7th row, generated only one set of instantaneous amplitude. However, 5 average frequencies were estimated with a window of 1, 3, 5, 7, 9 zero-crossings for smoothing purpose. The fuzzy-logic estimation (blue curve in Figure 3, 8th row), thresholding, merging and duration checking were performed identically as d_5_. Therefore, the vector of operating parameters for d_6_ was also identical to **x**_d5_.

### Three hybridization detectors

There are different ways in hybridizing different detectors for performance improvement. Simple approaches include intersection (total agreement) and union (partial agreement) of the results from multiple detectors. However, such a simple design through giving different weights to different detectors increase the computational cost cumulatively because it needs to collect the results from all participating detectors. Instead, in this study, we followed a double reading paradigm, in which the first detector screened all data to find potential sleep spindles as usual. However, the second detector only reviewed the sleep spindle candidates identified by the first detector. This approach was taken to balance between accuracy and computation cost.

We selected d_2_, d_4_, and d_6_ from the RMS, CWT, and HHT categories respectively for composing 3 hybridization detectors. First, hybrid detector d_7_ was composed of d_6_ as the first detector and d_2_ as the second detector. Its operating vector **x**_d7_ = {**x**_d6_, **x**_d2_} had 17 parameters in total. Second, hybrid detector d_8_ was composed of d_2_ as the first detector and d_6_ as the second detector. Its operating vector **x**_d8_ = {**x**_d2_, **x**_d6_} had 17 parameters. Third, hybrid detector d_9_ was composed of d_4_ as the first detector and d_6_ as the second detector. Its operating vector **x**_d9_ = {**x**_d4_, **x**_d6_} had 27 parameters.

### Subsample strategy

Since the total length of sleep spindles in the MASS SS2 datasets ranged from only 8 to 30 min per night, we decided to extract a subsample of 60 min from each whole night sleep in the training datasets to reduce SPEA2 computation time. Considering that our tested detectors used intrinsically different methods to estimate frequency, we did not want to create a common, single subsample for all of them. Instead, we tried to apply the optimal parameters derived from the DREAMS database by each individual detector to collect its own false positives from the training set of MASS database. These detector-dependent false positives and all true positives (defined by the chosen gold standard) were used to create small segments of signals. These small segments were dilated by 2.5 s from both ends and merged (if overlapped) to form a pool of bigger segments. From this pool, we randomly picked a total amount of 60 min long signals with true and false spindles. Tested with the maximal F_0.5_-, F_1_-, and F_2_-score (beta = 0.5, 1, 2 in Equation 3), we found that the parameters achieving the highest F_2_-score based on the union gold standard of the DREAMS database were a good choice for generating false detection subsamples from the MASS database.

### SPEA2 module

SPEA2 software has three mandatory input operating parameters: (1) solution population size, (2) maximum number of generations, and (3) minimum and maximum boundary values of the optimized detector's operating parameters. In this study, the solution population sizes and maximum generations were empirically chosen such that they were at least 10 times of the number of operating parameters. On several occasions, some of these numbers were increased by 50–100 than the aforementioned recommendations to ensure convergence to Pareto front solutions. Note that population size, number of generations, and parameter boundary values were empirically chosen in the training stage of the hold-out experiment described in the following section. Their values are listed in Section Spindle Detection Performance on DREAMS Database. Finally, for the other SPEA2 parameters (such as mutation and crossover) we used the default values implemented by Popov (2005).

### Statistics

For reporting the performance of each automatic spindle detector, we first derive the vector of operating parameters that achieves the maximal *F*_1_-score from its Pareto-optimal solutions solved by the SPEA2 algorithm based on a training dataset. Second, we apply the optimal operating parameter vector to a test dataset (unseen to the detector) to assess the resultant *F*_1_-score (Figure 2). Two validation strategies were conducted to assess the performance of spindle detectors in the proposed Pareto optimal and gold standard adaptive context. First, a hold-out strategy was used to assess overfitting by our SPEA2 implementations. Second, a k-fold cross-validation strategy was used to assess spindle detectors.

In our hold-out experiment, we divided the datasets into three groups: (1) all DREAMS datasets, (2) the first half of MASS datasets (subjects 1–9), and (3) the second half of MASS datasets (subjects 10–19). Subjects 7 and 8 were excluded from the DREAMS database if the gold standard of scorer 2 or intersection was applied; data 4, 8, 15, 16 were excluded from the MASS database if scorer 2's gold standard was applied. There were two versions of EEG signals in the MASS database. We used the new version published in 2015 to evaluate all the 9 detectors described in Sections Six Simplex Detectors and Three Hybridization Detectors. Our hold-out paradigm was conducted such that DREAMS and the first half of MASS were freely explored to find the proper ranges of adjusted parameters for each detector and to choose the proper population size and the number of generations for SPEA2. At this stage, we also experimented with a few different ways to generate an appropriate subsample for MASS datasets. Although subjecting a detector to 9 whole-night sleep EEG was not an impossibility, the idea of “gold standard adaptive” would work for most clinicians only if the training time was within hours by a regular personal computer. Once the parameter ranges, population size, number of generations, subsample strategy were decided, they were then fixed and used to train all the detectors with every different gold standard to find their own Pareto optimal solutions. The second half of MASS datasets that remained unseen to the spindle detectors during the entire training stage were then used to test the final solutions only once for deciding a conservative error bound (Brun et al., 2008).

The 95% confidence intervals (CI) of the estimated *F*_1_-scores by the training and test datasets in the hold-out experiment were derived by using probabilistic interpretation. Since the distributions of the precision and recall are Beta distributions (Goutte and Gaussier, 2005), the 95% CI of a F_1_-score can be estimated by running Monte Carlo simulation of precision and recall that are calculated from the data. We assumed that the number of true negatives in the data was 10 times the number of true positives and estimate the 95% CI by running Monte Carlo simulation for 10,000 times.

Lastly, 3-fold cross-validation, where the subjects in the MASS SS2 database were randomly partitioned into 3 equal sized subgroups, was conducted to assess the performance of 9 detectors. Of the 3 subgroups, a single subgroup was retained as the validation data for testing the detectors, and the remaining 2 subgroups were used as training data. In each fold, multiple Pareto optimal parameter vectors were solved by using a subsample of the training data as shown in Figure 2 for computation efficiency. However, the parameter vector **x** which generated the maximum F_1_-score was judged based on the complete-night training data. This maximum F_1_-score **x** was then used to assess the FN and FP results on the complete-night validation data. The cross-validation process was repeated 3 times with each of the 3 subgroups used exactly once as the validation data. The validation results from all 3-folds were finally grouped together to produce a single estimation.

### Software implementation

All the sleep spindle detection algorithms evaluated in this study were implemented in Matlab version R2015a (MathWorks, Natick, MA, USA) and C language. Fourier-based bandpass filtering software (d_1_, d_2_, and d_3_) was originally developed by Warby et al. (2014). The CWT-based software (d_4_) was developed by Tsanas and Clifford (2015). Parts of these Matlab codes were rewritten in C language for reducing SPEA2 computation time. The HHT-based software (d_5_ and d_6_) and hybrid software (d_7_, d_8_, and d_9_) was developed by our group. All Matlab and C source codes are available in Supplementary Material. Third party software such as SPEA2 and EMD is available in Popov (2005) and Wang et al. (2014) respectively. Computation time estimates were performed on a Fujitsu Lifebook laptop with Intel Core i7-3632QM processors at 2.20 GHz, using 12 GB of RAM memory running a 64-bit Windows 10 operating system.

## Results

### Spindle detection performance on dreams database

In the training stage of the hold-out experiment, we found that SPEA2 was able to converge to an adequate set of solutions with a population size of 100 parameter vectors that evolved 100 generations for detectors d_1_-d_3_ with 3–5 adjustable parameters. For detectors d_4_-d_9_ with more than 10 parameters, the population size was ascertained by 10 times the number of parameters and the number of generations was empirically determined by the population size plus 50–100 to ensure convergence to Pareto front solutions for the DREAMS database. In summary, the population sizes of d_1_-d_9_ were 100, 100, 100, 150, 120, 120, 170, 170, and 270 respectively. The maximum generations were 100, 100, 100, 250, 200, 200, 250, 250, and 320 respectively. The ranges of lower and upper parameter values were also empirically determined based the Pareto front results of the DREAMS database for all 9 detectors. The ascertained minimum and maximum (boundary) values of detectors d_1_-d_5_ parameters were

(13) $$x_{\text{d}1,\text{min}}=\left(0.1,\;0.1,\;0.3,\;0.3\right),$$

(14) $$x_{\text{d}1,\text{max}}=\left(20,\;30,\;1,\;3\right),$$

(15) $$x_{\text{d}2,\text{min}}=\left(0.05,\;0.05,\;0.1,\;0.3,\;0.3\right),$$

(16) $$x_{\text{d}2,\text{max}}=\left(0.5,\;0.5,\;10,\;1,\;3\right),$$

(17) $$x_{\text{d}3,\text{min}}=\left(0.1,\;0.3,\;0.3\right),$$

(18) $$x_{\text{d}3,\text{max}}=\left(99,\;1,\;3\right),$$

(19) $$\begin{array}{l} x_{\text{d}4,\text{min}}=(8,\;14,\;0.05,\;0.1,\;0.01,\;0.05,\;0.3,\;0.05,\;0.1,\;0.3,\\ \;0.05,\;0.05,\;0.1,\;0.3,\;0.3), \end{array}$$

(20) $$\begin{array}{l} x_{\text{d}4,\text{max}}=(12,\;18,\;0.25,\;0.8,0.1,\;0.25,\;0.9,\;0.25,\;0.5,\;0.8,\\ \;\;0.25,\;0.5,\;0.5,\;1,\;3), \end{array}$$

(21) $$x_{\text{d}5,\text{min}}=\left(1,5,10,1,0.1,8,1,0.1,0.01,0.05,0.3,0.3\right),$$

(22) $$x_{\text{d}5,\text{max}}=\left(10,40,120,50,4,13.5,8,4,0.99,0.5,\;1,\;3\right)$$

**Figure 4:**
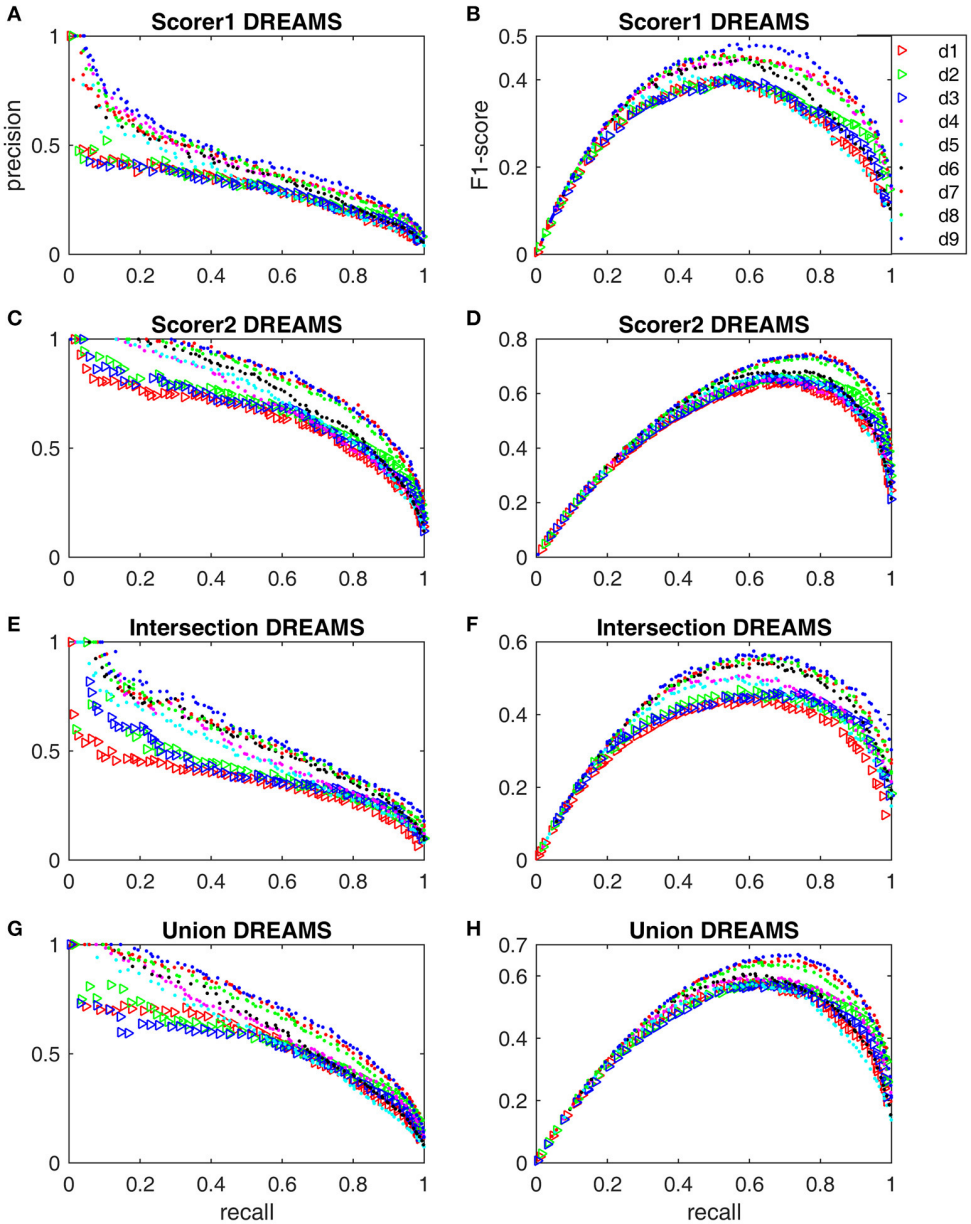
**Pareto front-derived PR-curves and F1-scores for the DREAMS database with scorer 1's gold standard (A,B)**, scorer 2's gold standard **(C,D)**, intersection gold standard **(E,F)**, and union gold standard **(G,H)**.

The minimum and maximum values of **x**_*d*6_ were the same as **x**_*d*5_. The hybrid detectors d_7_-d_9_ used the same minimum and maximum boundaries for their respective components d_2_, d_4_ and d_6_. The ranges of other parameters are given in Supplementary Material for code transparency and result reproducibility. Figure 4 shows the Pareto front-derived PR-curves and F_1_-scores of 9 sleep spindle detectors, simplex d_1_–d_6_ and hybrid d_7_–d_9_, solved by using the SPEA2 algorithm with the DREAMS database of 4 different gold standards listed in Table 1. The PR-curves (Figures 4A,C,E,G) and F_1_-scores (Figures 4B,D,F,H) vary dramatically from the worst performance evaluated based on scorer 1's gold standard to the gold standard of intersection, union, and finally to the best performance of scorer 2's gold standard. Although the F_1_-scores of all tested detectors vary, the hybrid detectors (especially d_9_) seem to perform better regardless of which gold standard is used.

### Spindle detector hold-out validation on mass database

**Figure 5:**
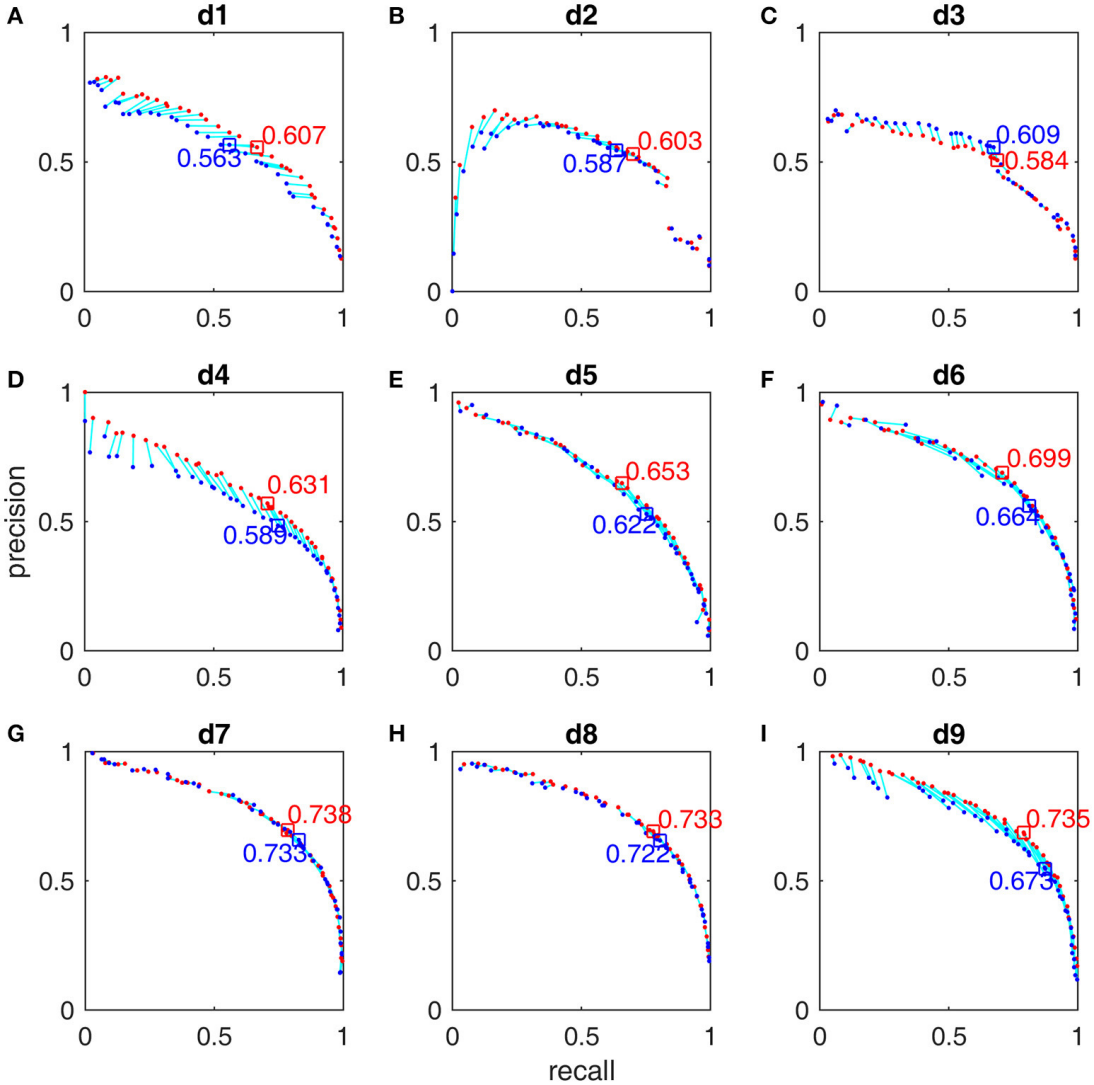
**Pareto-front-derived PR-curves for the MASS database by scorer 1's gold standard for detectors d_1_ (A)**, d_2_ **(B)**, d_3_ **(C)**, d_4_ **(D)**, d_5_**(E)**, d_6_ **(F)**, d_7_ **(G)**, d_8_ **(H)**, and d_9_ **(I)**. The training results (red) and the test results (blue) are connected by light blue lines to indicate that they are derived by using the same vector of operating parameters. The maximal training F_1_-scores and their corresponding test results are marked with squares and their values presented.

**Figure 6:**
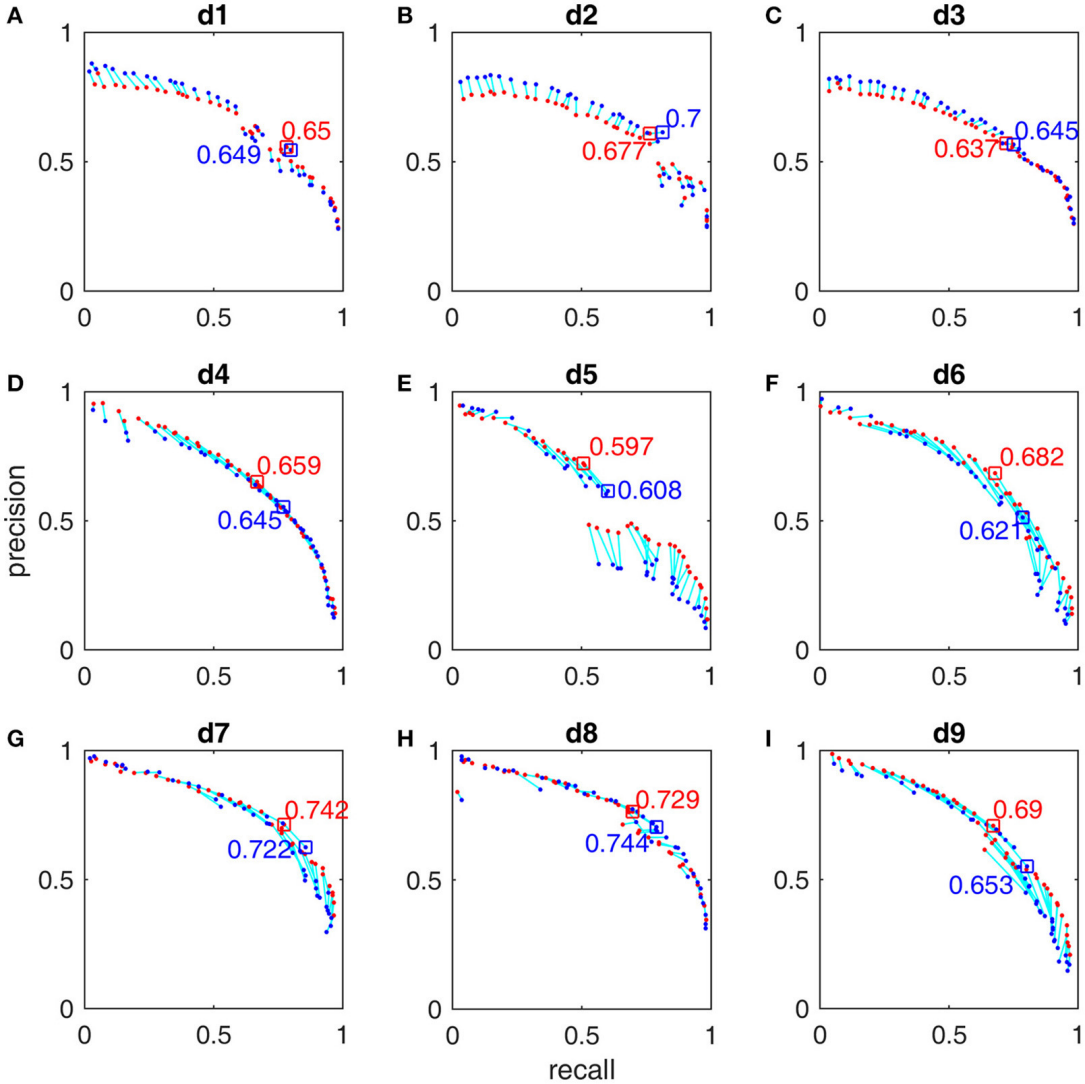
**Pareto-front-derived PR-curves for the MASS database by scorer 2's gold standard for detectors d_1_ (A)**, d_2_ **(B)**, d_3_ **(C)**, d_4_ **(D)**, d_5_**(E)**, d_6_ **(F)**, d_7_ **(G)**, d_8_ **(H)**, and d_9_ **(I)**. The training results (red) and the test results (blue) are connected by light blue lines to indicate that they are derived by using the same vector of operating parameters. The maximal training F_1_-scores and their corresponding test results are marked with squares and their values presented.

After we trained these 9 detectors with the subsamples extracted from the first half of MASS database by using the subsample strategy (Section Subsample Strategy), we reported their training performance based on the FN and FP calculated from the whole night training datasets with the Pareto optimal solutions derived from subsamples. The Pareto front-derived PR-curves estimated in the training procedure are shown as red dots in Figures 5, 6 for the gold standard of scorers 1 and 2 respectively. The PR-values of test results derived from the second half of MASS database are shown as blue dots (in Figures 5, 6) and are connected by a light blue line to the training results (red dots) that are estimated by using the same vector of operating parameters. The maximal F_1_-scores acquired by training are marked by red squares in Figures 5, 6. Note that the blue squares in Figures 5, 6 are not the maximal test F_1_-scores but the test results by using the same set of operating parameters of the corresponding red squares. Here we did not report the maximal test F_1_-scores because in real practice we can choose only a small number of operating parameters after the software system has been optimized. The PR-values, F_1_-scores, and 95% CI of F_1_ at the operating points marked by squares in Figures 5, 6 are listed in Table 3 and their operating parameter values are given in Supplementary Material.

**Table 3:** **Sleep spindle detector performance evaluation**.

**Detector**	**Scorer 1's gold standard**				**Scorer 2's gold standard**			
	**P**	**R**	**F1**	**F1 [95% CI]**	**P**	**R**	**F1**	**F1 [95% CI]**
d_1_ train	0.557	0.668	0.607	[0.597, 0.618]	0.556	0.782	0.650	[0.643, 0.657]
test	0.567	0.560	0.563	[0.552, 0.574]	0.546	0.799	0.649	[0.642, 0.655]
3-fold	0.544	0.639	0.588	–	0.601	0.738	0.662	–
baseline	0.481	0.360	0.412	–	0.599	0.194	0.293	–
d_2_ train	0.531	0.699	0.603	[0.593, 0.614]	0.609	0.764	0.677	[0.671, 0.684]
test	0.546	0.636	0.587	[0.578, 0.598]	0.614	0.814	0.700	[0.694, 0.706]
3-fold	0.500	0.762	0.604	–	0.594	0.788	0.677	–
baseline	0.268	0.970	0.420	–	0.538	0.807	0.646	–
d_3_ train	0.508	0.688	0.584	[0.574, 0.595]	0.571	0.721	0.637	[0.630, 0.644]
test	0.556	0.673	0.609	[0.599, 0.619]	0.567	0.748	0.645	[0.638, 0.652]
3-fold	0.508	0.684	0.583	–	0.577	0.748	0.651	–
baseline	0.267	0.943	0.416	–	0.535	0.751	0.625	–
d_4_ train	0.570	0.708	0.631	[0.621, 0.642]	0.650	0.667	0.659	[0.651, 0.666]
test	0.485	0.749	0.589	[0.579, 0.598]	0.554	0.770	0.645	[0.638, 0.651]
3-fold	0.535	0.710	0.610	–	0.627	0.677	0.651	–
baseline	0.177	0.980	0.300	–	0.352	0.845	0.497	–
d_5_ train	0.648	0.657	0.653	[0.642, 0.663]	0.724	0.508	0.597	[0.589, 0.606]
test	0.531	0.751	0.622	[0.613, 0.631]	0.615	0.601	0.608	[0.601, 0.616]
3-fold	0.601	0.670	0.633	–	0.605	0.688	0.644	–
baseline	0.228	0.884	0.362	–	0.623	0.660	0.641	–
d_6_ train	0.690	0.708	0.699	[0.689, 0.709]	0.684	0.680	0.682	[0.674, 0.689]
test	0.562	0.812	0.664	[0.655, 0.673]	0.514	0.786	0.621	[0.615, 0.628]
3-fold	0.649	0.712	0.679	–	0.643	0.697	0.669	–
baseline	0.051	0.873	0.097	–	0.138	0.903	0.240	–
d_7_ train	0.697	0.783	0.738	[0.729, 0.747]	0.714	0.773	0.742	[0.736, 0.749]
test	0.659	0.827	**0.733**	**[0.725, 0.742]**	0.624	0.857	0.722	[0.716, 0.728]
3-fold	0.720	0.745	**0.732**	–	0.699	0.781	**0.737**	–
d_8_ train	0.693	0.779	0.733	[0.724, 0.742]	0.764	0.696	0.729	[0.722, 0.736]
test	0.656	0.804	0.722	[0.714, 0.731]	0.705	0.789	**0.744**	**[0.738, 0.750]**
3-fold	0.706	0.749	0.727	–	0.685	0.774	0.726	
d_9_ train	0.686	0.791	0.735	[0.725, 0.744]	0.709	0.671	0.690	[0.682, 0.697]
test	0.548	0.874	0.673	[0.664, 0.681]	0.551	0.802	0.653	[0.647, 0.660]
3-fold	0.657	0.748	0.700	–	0.658	0.703	0.680	–

*CI stands for confidence intervals and the best hold-out test results are bold faced. 3-fold stands for 3-fold cross validation. The baseline performance is the result of using the suggested parameters of the original publication*.

**Figure 7:**
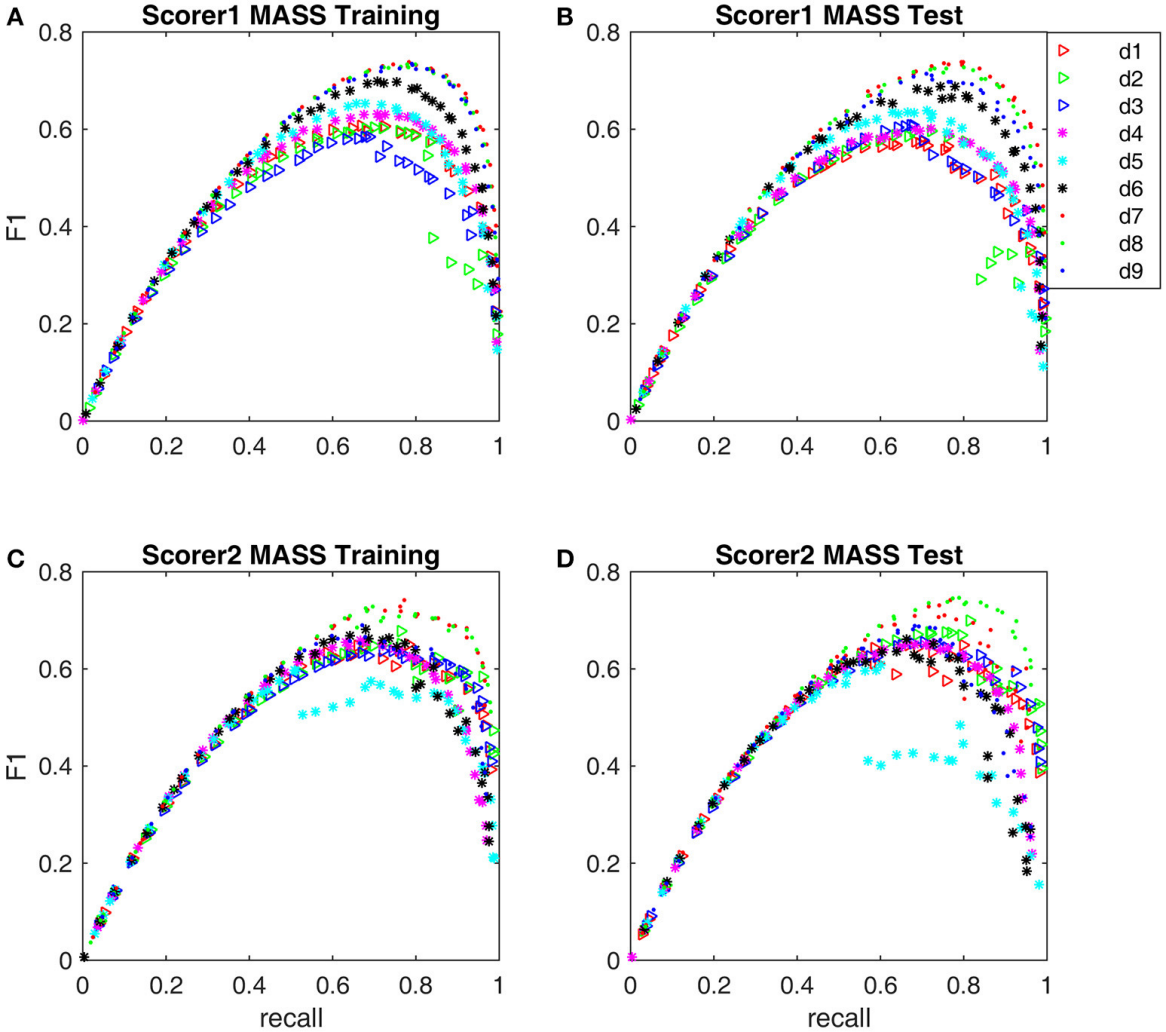
**The Pareto front-derived F_1_-scores of detectors d_1_-d_9_ are shown along the recall coordinate. (A)** Training results by scorer 1's gold standard. **(B)** Test results by scorer 1's gold standard. **(C)** Training results by scorer 2's gold standard. **(D)** Test results by scorer 2's gold standard.

**Figure 8:**
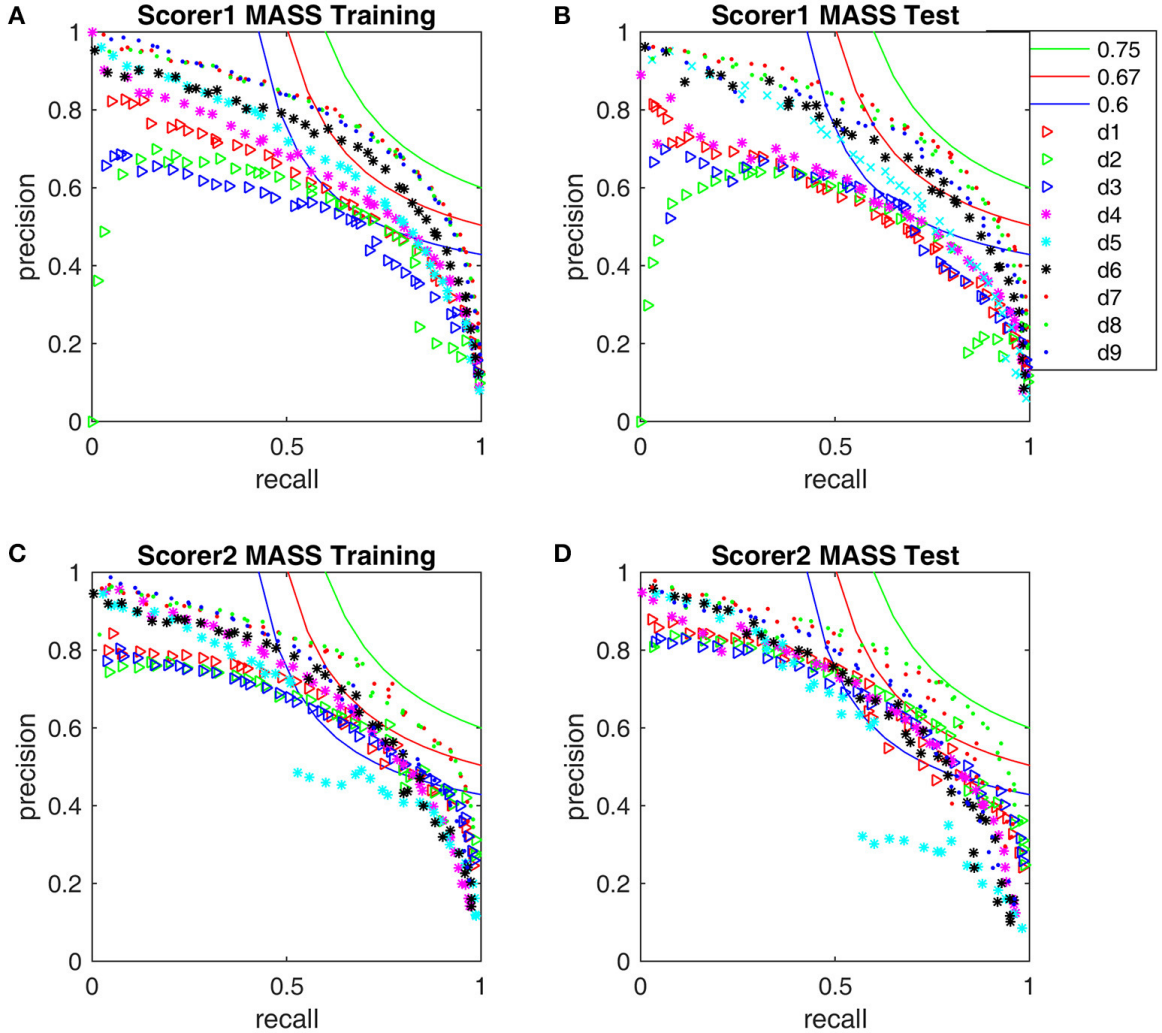
**Performance comparison with the iso-curves at F_1_-scores of 0.75, 0.67, and 0.6. (A)** The training PR-curves on the first half MASS database and **(B)** the test PR-curves on the second half MASS database by scorer 1's gold standard. **(C)** The training PR-curves on the first half MASS database and **(D)** the test PR-curves on the second half MASS database by scorer 2's gold standard.

The F_1_-scores for all 9 detectors by the gold standards of scorer 1 and scorer 2 are illustrated in Figure 7. From Figures 7A,B, Table 3, we observe that Fourier-based simplex detectors d_1_–d_3_ perform the worst with the estimated maximal F_1_-scores 0.563–0.609 by scorer 1's gold standard. HHT-based simplex detectors perform better with the estimated maximal F_1_-scores 0.622–0.699. Hybrid detectors d_7_ and d_8_ perform the best with the estimated maximal F_1_-scores 0.722–0.738. CWT-based simplex detector d_4_ and hybrid d_9_ perform well with high training F_1_-scores at 0.631 and 0.735 but low test results of 0.589 and 0.673 respectively. The results in Table 3 indicate that Fourier-based detectors improve significantly by scorer 2's gold standard [scored based on both broad-band EEG signals (0.35–35 Hz band) and sigma-band filtered signals (11–17 Hz band)]. Despite the existence of a biased gold standard in favor of Fourier filtering, hybrid d_7_ and d_8_ still outperform with maximal F_1_-scores 0.722–0.744 compared to 0.637–0.7 by simplex Fourier-based detectors. Finally, automatic detectors are compared to the iso-curves of F_1_-scores of 0.75 (good) and 0.67 (average) in Figure 8. The performance levels of “good” and “average” refer to the study by Warby et al. (2014), where experts' average F_1_ was 0.75 and non-experts' consensus 0.67. Figures 8A,B illustrate that the optimized simplex detectors d_1_-d_5_ are indeed inferior to human performance of 0.67. However, HHT-based simplex detector d_6_ perform comparably to the level of non-experts. Hybrid detectors d_7_ and d_8_ perform well on a level slightly below that of experts regardless which gold standard is used (Figures 8A–D).

### Spindle detector 3-fold cross-validation on mass database

The maximum F_1_-scores of d_1_–d_9_ estimated by 3-fold cross-validation were 0.588, 0.604, 0.583, 0.61, 0.633, 0.679, 0.732, 0.727, and 0.7 respectively based on scorer 1's gold standard. They were 0.622, 0.677, 0.651, 0.651, 0.644, 0.669, 0.737, 0.726, and 0.68 respectively based on scorer 2's gold standard. Most of these numbers are substantially better than the baseline performance by using their originally published parameters. Both 3-fold validation, baseline performance, and their corresponding PR-values are also listed in Table 3 for comparison. These cross-validation estimates were comparable to the hold-out estimates except d_5_'s F_1_-score on scorer 2's gold standard. The hold-out test F_1_-score was 0.608 (95% confidence intervals 0.601–0.616), which was significantly smaller than the cross-validation's F_1_ estimate of 0.644.

### Computation time

The SPEA2 training time for d_1_–d_3_, d_4_, and d_5_–d_6_ was 27–42 m, 5.3 h, and 1.7–2.2 h, respectively; for hybrid detectors d_7_, d_8_, and d_9_ was 4.8, 4.4, and 16 h, respectively. Although the computation time (in seconds) to preprocess the C3-A1 channel of a complete-night MASS dataset was 0.9, 7, 16, 28, 73, 835, 838, 390, 390 for d_1_–d_9_ respectively, the execution time of spindle detectors d_1_–d_9_ implemented in C language was 0.06, 0.09, 0.16, 0.63, 0.20, 0.14, 0.22, 0.27, and 0.71, which is well below 1 s per complete-night dataset. Therefore, the training time was mainly proportional to the product of population size and number of generations, which approximated the square of a detector's parameter number.

## Discussion

Table 4 summarizes four previous published evaluation methodologies and our new approach. From Table 4, We identify three important trends in evaluating automatic sleep spindle detection. First, using open-access databases (Devuyst et al., 2011; O'Reilly and Nielsen, 2015) and/or making software source codes open-access (Warby et al., 2014; O'Reilly and Nielsen, 2015) are the two most important factors in advancing our understanding of performance improvement of various spindle detectors. Second, using non-specificity-derived metrics such as F_1_-score (Warby et al., 2014; O'Reilly and Nielsen, 2015) for reporting performance is another important trend to make results from different studies comparable. Third, a newly developing trend identified by O'Reilly and Nielsen (2015) is to involve gold standards in the process of performance estimation. To make this idea work, we suggest that commonly acceptable evaluation metrics for spindle detection should be gold standard adaptive.

**Table 4:** **Comparison of evaluation methods for sleep spindle detection**.

	**Huupponen et al., 2007**	**Devuyst et al., 2011**	**Warby et al., 2014**	**O'Reilly and Nielsen, 2015**	**New method**
Open-access database used	No	DREAMS	No	DREAMS and MASS	DREAMS and MASS
Number of evaluated detectors	4	1	6	4	9
Detector source code open-access	unspecified	No	Yes	Yes	Yes
Operating parameter adjustment	According to frequency and amplitude statistics of sleep spindles	According to frequency and amplitude statistics of sleep spindles	Using the original published setting	Using the original published setting	Multiple parameters were optimized by MOEA
True detection criterion	By-event	By-event	By-event (with 0.2 overlap rate)	By-event and by-sample	By-event (with 0.2 overlap rate)
Evaluation metrics	Sensitivity, specificity, ROC-curve	Sensitivity, specificity, ROC-curve	PR, F_1_-score	ROC-, PR-curve, F_1_-score, Matthew's correlation coefficient, Cohen's Kappa	PR-curve, F_1_-score, Pareto front
Statistics	Parameters were set and tested on the same data	Parameters were set and tested on the same data	Using default parameters and threshold	Threshold-dependent	Hold-out, 3-fold cross validation

Here we first discuss the necessity of being gold standard adaptive in evaluating a sleep spindle detector. Figure 4 shows that the performance of a detector on the same database by different gold standards can vary at most by a difference of 0.2 in F_1_-score. One may argue that the DREAMS database is too small for an adequate evaluation. However, as an example, take d_2_ in Table 3 evaluated based on the MASS database, the F_1_-score still changed noticeably from a low level of 0.604 by scorer 1's gold standard to an average (non-expert human's) level of 0.677 by scorer 2's. Although the score of 0.677 is still considerably lower than 0.726–0.737 by d_7_ and d_8_, d_2_ is a much faster detector that can be useful in processing a big database of thousands of patients if the gold standard of scorer 2 is adequate for our study purpose. On the other hand, for performance accuracy and consistency, d_7_ maybe the most adaptive performer with a F_1_-score of 0.727 and 0.737 no matter which gold standard was considered. The point we want to emphasize is that the definition of true spindles may be age- or disease-dependent. In addition, some applications may require a higher duration accuracy criterion by making the overlap rate *R*_*ov*_ (Equation 5) another adjustable parameter. Yet, other applications may want to adjust the ranges of a Fourier filter's sigma-band for detecting slow or fast spindles. Therefore, the so-called optimal detector should not only be gold standard adaptive but also application-dependent.

Second, a commonly accepted performance metric that is suitable for gold standard adaptive should be formally and uniquely defined. Although PR-curves and F_1_-scores are known to be good metrics and commonly used in evaluating detectors of sparse events such as sleep spindles, Figure 1 demonstrates that PR-curves and F_1_-scores derived from non-optimal operating points are not uniquely defined (as compared to the uniquely defined Pareto front) even for a simple detector with 3 adjustable parameters. The situations to define *ad hoc* PR-curves and F_1_-scores for more complex detectors such as d_4_–d_6_ deteriorated and d_9_ with 27 parameters will be an almost impossibility. In this study, we propose evaluating a spindle detector in a multi-objective optimization context with the resultant Pareto fronts for deriving PR-curves and F1-scores formally and uniquely. A Pareto front is essentially an objective boundary such that any solution on the front can only be outperformed by another solution in at most one of the two competing objectives. Therefore, a Pareto optimal set is uniquely defined for a given data with a specific gold standard. We demonstrated that the Pareto fronts can be efficiently solved by SPEA2 for spindle detectors with the proposed subsample strategy.

Third, as evolutionary algorithms are able to solve the optimization problems with implicit solutions that are not easily foreseen in the process of designing a new detector, we also demonstrated the possibility to develop new hybrid methods by combining two existing simplex detectors. Note that among 6 simplex detectors d_1_-d_6_, choosing two in an order yields 30 possible hybrid approaches. We did not perform all 30 possible combinations because we did not have the resources to do a full-fledged hybridization experiment. Detectors d_4_-d_5_ and d_4_-d_6_ combinations with 27 operating parameters were particularly time-consuming. As our main goal was to prove that spindle detectors would be improved by using MOEA, we only tested the hybridization approach on HHT-Fourier and HHT-CWT combinations. The rationale was that hybridization usually would perform best by combining different computational mechanisms. Fourier and wavelet are rather similar because both methods use inner product in deriving their coefficients. On the other hand, HHT uses a sifting procedure that is totally different from the inner product procedure. Therefore, we chose the better HHT-based algorithm d_6_ to hybridize with the Fourier-based d_2_ and CWT-based d_4_. Despite using a very simple double reading design in which the first detector screens all data to find potential sleep spindles and the second detector only reviews the sleep spindle candidates identified by the first detector, this sequential hybridization system allows us to design two very effective hybrid detectors, d_7_ and d_8_, by simply switching the order of applied simplex detectors d_2_ and d_6_. They achieved the highest F_1_-scores of 0.726–0.737 that were notably better than their composite components d_2_ and d_6_ of 0.604–0.679.

Last, our experiment was conducted in both hold-out and k-fold cross-validation paradigms. The hold-out validation allowed us to derive both model-based 95% CI and a more conservative error bound estimate (Brun et al., 2008) to assess overfitting. Take the best performers d_7_–d_9_ listed in Table 3 for example. Detector d_9_ performed at a good level with its training F_1_-score 0.735 but an average hold-out test score 0.673 (by scorer 1's gold standard), which was much lower than the 95% CI 0.725–0.744 via Monte Carlo simulation at the maximal *F*_1_ = 0.735 from the training datasets. The error bound estimated by a hold-out design is larger and therefore more conservative than the model-based estimation. We double-checked its 3-fold result, which was 0.7. Therefore, the possibility of overfitting did exist. The similar poorer hold-out test scores were also observed for d_4_ (scorer 1's gold standard) and d_6_ (scorer 2's gold standard). This implied that these detectors were more susceptible to subsampling. For example, one possible explanation was that the 60 min long subsamples were not chosen properly by chance. Another possible explanation was that detectors d_4_-d_6_ did not use any amplitude normalization strategy. On the other hand, the hold-out and 3-fold estimated F_1_-scores of d_7_ (scorer 1's gold standard) were 0.733 and 0.732, which fell well in the 95% CI 0.729–0.747 via Monte Carlo simulation of the maximal *F*_1_ = 0.738 from the training datasets. Since d_7_ and d_8_ all performed with F_1_-scores well above 0.7 by both model-based and hold-out error bound estimations, their performance improvement over d_1_–d_6_ was considered statistically significant.

In conclusion, our study has demonstrated that using multi-objective evolutionary algorithms to optimize automatic sleep spindle detectors in a gold standard adaptive approach can potentially improve the effectiveness and consistency of sleep spindle identification and make the analysis of sleep spindle properties more reliable in clinical settings.

## Author contributions

AH was responsible for the overall study design, development, and management. ML and AH involved in coding, processing data, and interpretation of the results. NH provided methodological and computational expertise. All authors made substantial contributions to the drafting, critical revision of the important intellectual content and final approval of the manuscript.

## Funding

This study was supported by the Ministry of Science and Technology Taiwan under Grant MOST 105-2221-E-008-114 and 103-2911-I-008-001.

### Conflict of interest statement

The authors declare that the research was conducted in the absence of any commercial or financial relationships that could be construed as a potential conflict of interest.
