# Supplementary material for: Evaluating and Improving Automatic Sleep Spindle Detection by Using Multi-Objective Evolutionary Algorithms
Source: Front Hum Neurosci. 2017 May 18;11:261. doi: 10.3389/fnhum.2017.00261 (PMC5435763; doi:10.3389/fnhum.2017.00261)
Supplement: Supplementary file 1 [file Presentation1.PDF]

# Supplementary Material

## Evaluating and Improving Automatic Sleep Spindle Detection by Using Multi-Objective Evolutionary Algorithms

Min-Yin Liu[1], Adam Huang[2,]*, Norden E. Huang[1,2]

[1]Institute of Systems Biology and Bioinformatics, Department of Biomedical Sciences and Engineering, National Central University, Taoyuan 32001, Taiwan
[2]Research Center for Adaptive Data Analysis, National Central University, Taoyuan 32001, Taiwan

*Correspondence:
Adam Huang, PhD
adamhuan@ncu.edu.tw
adamhuan@gmail.com

## Detectors' Optimized Operating Parameters Solved in the Hold-out Training Stage by Using Scorers 1's, 2's Gold Standards and the Original Default Settings

**Table S1. Detector $d_1$'s optimized and default operating parameters**

| | Lower amplitude threshold ratio | Upper amplitude threshold ratio | Lower duration (s) | Upper duration (s) |
|---|---|---|---|---|
| $\mathbf{X}_{d1}$ | $p_{1,1}$ | $p_{1,2}$ | $p_{1,3}$ | $p_{1,4}$ |
| 1's | 3.4992 | 6.1048 | 0.8218 | 2.9844 |
| 2's | 2.0938 | 3.7034 | 0.8006 | 2.9920 |
| default | 2 | 8 | 0.3 | 3 |

**Table S2. Detector $d_2$'s optimized and default operating parameters**

| | RMS Time resolution (s) | RMS window (s) | Upper RMS threshold ratio | Lower duration (s) | Upper duration (s) |
|---|---|---|---|---|---|
| $\mathbf{X}_{d2}$ | $p_{2,1}$ | $p_{2,2}$ | $p_{2,3}$ | $p_{2,4}$ | $p_{2,5}$ |
| 1's | 0.0504 | 0.0705 | 1.8961 | 0.4412 | 2.7958 |
| 2's | 0.2374 | 0.4927 | 1.3564 | 0.3834 | 2.7097 |
| default | 0.05 | 0.1 | 1.5 | 0.3 | 3 |

**Table S3. Detector $d_3$'s optimized and default operating parameters**

| | Upper RMS threshold (percentile) | Lower duration (s) | Upper duration (s) |
|---|---|---|---|
| $\mathbf{X}_{d3}$ | $p_{3,3}$ | $p_{3,4}$ | $p_{3,5}$ |
| 1's | 94.6105 | 0.6509 | 2.0138 |
| 2's | 79.0110 | 0.9974 | 2.8316 |
| default | 95 | 0.3 | 3 |

**Table S4. Detector $d_4$'s optimized and default operating parameters**

| | Lower freq (Hz) | Upper freq (Hz) | Smooth window (s) | Thres-hold | Min duration (s) | Merge gap (s) | Strong threshold | Dura-tion (s) |
|---|---|---|---|---|---|---|---|---|
| $\mathbf{X}_{d4}$ | $p_{4,1}$ | $p_{4,2}$ | $p_{4,3}$ | $p_{4,4}$ | $p_{4,5}$ | $p_{4,6}$ | $p_{4,7}$ | $p_{4,8}$ |
| 1's | 11.8479 | 15.368 | 0.2474 | 0.6726 | 0.0698 | 0.0791 | 0.8601 | 0.2494 |
| 2's | 11.9869 | 15.039 | 0.2404 | 0.4427 | 0.0808 | 0.0626 | 0.6583 | 0.1902 |
| default | 11 | 16 | 0.11 | 0.3 | 0.03 | 0.1 | 0.7 | 0.1 |

| Dura-tion (s) | Inter-mediate threshold | Dura-tion (s) | Dura-tion (s) | Merge time gap (s) | Lower duration (s) | Upper Mergeable duration (s) |
|---|---|---|---|---|---|---|
| $p_{4,9}$ | $p_{4,10}$ | $p_{4,11}$ | $p_{4,12}$ | $p_{4,13}$ | $p_{4,14}$ | $p_{4,15}$ |
| 0.1003 | 0.3176 | 0.0504 | 0.4057 | 0.4794 | 0.3245 | 2.9207 |
| 0.2832 | 0.5567 | 0.0575 | 0.3233 | 0.4949 | 0.3492 | 2.9936 |
| 0.3 | 0.6 | 0.1 | 0.2 | 0.3 | 0.4 | 1.5 |

**Table S5. Detector $d_5$'s optimized and default operating parameters**

| | Amplitude fuzzy logic parameters (uV) | | | | Frequency fuzzy logic parameters (Hz) | | | |
|---|---|---|---|---|---|---|---|---|
| $\mathbf{X}_{d5}$ | $p_{5,1}$ | $p_{5,2}$ | $p_{5,3}$ | $p_{5,4}$ | $p_{5,5}$ | $p_{5,6}$ | $p_{5,7}$ | $p_{5,8}$ |
| 1's | 6.5427 | 13.461 | 38.477 | 42.525 | 3.5135 | 12.380 | 2.7642 | 1.6175 |
| 2's | 2.9939 | 6.0575 | 74.915 | 39.327 | 3.9688 | 13.424 | 1.0034 | 2.7247 |
| default | 5 | 15 | 105 | 30 | 0.5 | 10 | 6 | 0.5 |

| Thres-hold | Merge time gap (s) | Lower duration (s) | Upper duration (s) |
|---|---|---|---|
| $p_{5,9}$ | $p_{5,10}$ | $p_{5,11}$ | $p_{5,12}$ |
| 0.1657 | 0.0500 | 0.4899 | 2.5457 |
| 0.0363 | 0.0503 | 0.7581 | 2.9536 |
| 0.5 | 0.2 | 0.3 | 3 |

**Table S6. Detector $d_6$'s optimized and default operating parameters**

| | Amplitude fuzzy logic parameters (uV) | | | | Frequency fuzzy logic parameters (Hz) | | | |
|---|---|---|---|---|---|---|---|---|
| **$X_{d6}$** | $p_{6,1}$ | $p_{6,2}$ | $p_{6,3}$ | $p_{6,4}$ | $p_{6,5}$ | $p_{6,6}$ | $p_{6,7}$ | $p_{6,8}$ |
| 1's | 9.5836 | 25.473 | 57.204 | 7.2513 | 1.7971 | 13.368 | 1.0409 | 0.1004 |
| 2's | 9.8418 | 21.325 | 59.971 | 12.735 | 0.3092 | 13.194 | 1.0204 | 0.2523 |
| default | 5 | 15 | 105 | 30 | 0.5 | 10 | 6 | 0.5 |

| Thres-hold | Merge time gap (s) | Lower duration (s) | Upper duration (s) |
|---|---|---|---|
| $p_{6,9}$ | $p_{6,10}$ | $p_{6,11}$ | $p_{6,12}$ |
| 0.3574 | 0.0501 | 0.4312 | 2.9909 |
| 0.4068 | 0.1027 | 0.3946 | 2.9998 |
| 0.5 | 0.2 | 0.3 | 3 |

**Table S7. Detector $d_7$'s optimized and default operating parameters**

| | Amplitude fuzzy logic parameters (uV) | | | | Frequency fuzzy logic parameters (Hz) | | | |
|---|---|---|---|---|---|---|---|---|
| **$X_{d7}$** | $p_{6,1}$ | $p_{6,2}$ | $p_{6,3}$ | $p_{6,4}$ | $p_{6,5}$ | $p_{6,6}$ | $p_{6,7}$ | $p_{6,8}$ |
| 1's | 5.6312 | 18.208 | 117.07 | 18.625 | 3.9949 | 13.450 | 1.4203 | 1.2140 |
| 2's | 9.6855 | 16.422 | 100.15 | 5.0105 | 0.1529 | 12.328 | 5.5736 | 3.7623 |

| Thres-hold | Merge time gap (s) | Lower duration (s) | Upper duration (s) | RMS Time resolution (s) | RMS window (s) | Upper RMS threshold ratio | Lower duration (s) | Upper duration (s) |
|---|---|---|---|---|---|---|---|---|
| $p_{6,9}$ | $p_{6.10}$ | $p_{6.11}$ | $p_{6,12}$ | $p_{2,1}$ | $p_{2,2}$ | $p_{2,3}$ | $p_{2,4}$ | $p_{2,5}$ |
| 0.6193 | 0.0503 | 0.4438 | 2.9987 | 0.0505 | 0.4896 | 1.4879 | 0.5431 | 2.9589 |
| 0.3793 | 0.0508 | 0.3439 | 2.9996 | 0.2602 | 0.4978 | 0.9646 | 0.4569 | 2.9869 |

**Table S8. Detector $d_8$'s optimized and default operating parameters**

| | RMS Time resolution (s) | RMS window (s) | Upper RMS threshold ratio | Lower duration (s) | Upper duration (s) | Amplitude fuzzy logic parameters (uV) | | |
|---|---|---|---|---|---|---|---|---|
| $\mathbf{X}_{d8}$ | $p_{2,1}$ | $p_{2,2}$ | $p_{2,3}$ | $p_{2,4}$ | $p_{2,5}$ | $p_{6,1}$ | $p_{6,2}$ | $p_{6,3}$ |
| 1's | 0.0501 | 0.4849 | 1.7724 | 0.3025 | 2.9903 | 8.8610 | 17.983 | 95.442 |
| 2's | 0.0529 | 0.4669 | 1.3288 | 0.4150 | 2.5127 | 4.4462 | 13.250 | 112.85 |

| | Frequency fuzzy logic parameters (Hz) | | | | Thres-hold | Merge time gap (s) | Lower duration (s) | Upper duration (s) |
|---|---|---|---|---|---|---|---|---|
| $p_{6,4}$ | $p_{6,5}$ | $p_{6,6}$ | $p_{6,7}$ | $p_{6,8}$ | $p_{6,9}$ | $p_{6,10}$ | $p_{6,11}$ | $p_{6,12}$ |
| 43.961 | 1.0969 | 12.420 | 4.9864 | 1.2069 | 0.8015 | 0.0500 | 0.4456 | 2.8276 |
| 28.152 | 1.1942 | 12.967 | 5.2226 | 2.3198 | 0.1145 | 0.0511 | 0.4960 | 2.8055 |

**Table S9. Detector $d_9$'s optimized and default operating parameters**

| | Lower freq (Hz) | Upper freq (Hz) | Smooth window (s) | Thres-hold | Min duration (s) | Merge gap (s) | Strong threshold | Dura-tion (s) |
|---|---|---|---|---|---|---|---|---|
| $\mathbf{X}_{d9}$ | $p_{4,1}$ | $p_{4,2}$ | $p_{4,3}$ | $p_{4,4}$ | $p_{4,5}$ | $p_{4,6}$ | $p_{4,7}$ | $p_{4,8}$ |
| 1's | 12.000 | 17.754 | 0.2472 | 0.3766 | 0.0806 | 0.1163 | 0.8624 | 0.2328 |
| 2's | 11.995 | 17.528 | 0.0566 | 0.3219 | 0.0201 | 0.1055 | 0.6659 | 0.0528 |

| Dura-tion (s) | Inter-mediate threshold | Dura-tion (s) | Dura-tion (s) | Merge time gap (s) | Lower duration (s) | Upper Mergeable duration (s) | Amplitude fuzzy (uV) | |
|---|---|---|---|---|---|---|---|---|
| $p_{4,9}$ | $p_{4,10}$ | $p_{4,11}$ | $p_{4,12}$ | $p_{4,13}$ | $p_{4,14}$ | $p_{4,15}$ | $p_{6,1}$ | $p_{6,2}$ |
| 0.2273 | 0.5541 | 0.0571 | 0.1099 | 0.4941 | 0.3874 | 0.3402 | 6.0425 | 21.932 |
| 0.4401 | 0.3022 | 0.2230 | 0.3969 | 0.4964 | 0.3393 | 2.9070 | 9.9895 | 23.346 |

| logic parameters | | Frequency fuzzy logic parameters (Hz) | | | | Thres-hold | Merge time gap (s) | Lower duration (s) |
|---|---|---|---|---|---|---|---|---|
| $p_{6,3}$ | $p_{6,4}$ | $p_{6,5}$ | $p_{6,6}$ | $p_{6,7}$ | $p_{6,8}$ | $p_{6,9}$ | $p_{6,10}$ | $p_{6,11}$ |
| 71.339 | 47.492 | 3.6139 | 10.766 | 3.6235 | 2.1585 | 0.7359 | 0.0670 | 0.3578 |
| 58.796 | 7.9279 | 2.0020 | 13.497 | 3.7117 | 0.8504 | 0.7427 | 0.4996 | 0.3512 |

| Upper duration (s) |
|---|
| $p_{6,12}$ |
| 2.9826 |
| 2.9994 |

# Step-by-Step Instructions for Setting up Software and Experiments

```
% ... Steps in Setting up Software and the Experiment ...

% % % % % % % % % % % % % % % % % % % % % % % % % % % % % % % % %
% 0.1 download SPEA2
%     A Matlab version can be downloaded from
%     http://p0p0v.com/science/downloads/MOEA_SPEA2.zip
%     accessed 2016/10/10
%     put moea.m and spea2.mexw64 (compiled with mex)
%     in the working directory
% 0.2 Preprocess data
%     Use a0_preprocess_data.m as an example
% % % % % % % % % % % % % % % % % % % % % % % % % % % % % % % % %

% %.......... beginning of 'a0_preprocess_data.m' ...........
function a0_preprocess_data
% Please arrange the data file structure as follows:
%     C3 (Nx1 vector)
%     stage (Nx1 vector), Wake,REM,Unknown valued as 2,1,0
%                         NREM S1,S2,S3,S4 valued as -1,-2,-3,-4
%     fs (scalar) sample frequency
%     D1 (N1x2) head-tail pairs of spindles at sample location
%               by expert 1 ([100 250;... length(C3)])
%     D2 (N2x2) by expert 2
%     DI (NIx2) by intersection of 1&2 (for DREAMS only)
%     DU (NUx2) by union of 1&2 (for DREAMS only)
% in a single file 'data1.mat', 'data2.mat',...
% DREAMS data stored at '../DREAMS/data1.mat','../DREAMS/data2.mat'
% MASS data stored at '../MASS/data1.mat','../MASS/data2.mat',...
for i=1:8 % for DREAMS database
    % First, arrange C3, stage, fs, D1, D2, DI, DU as described above
    % for DREAMS database from data1 to data8.
    save(['../DREAMS/data',num2str(i),'.mat'],'C3','stage','fs',...
        'D1','D2','DI','DU');
end
for i=1:19 % for MASS database
    % First, arrange C3, stage, fs, D1, D2 as described above for
    % MASS database from data1 to data19.
    save(['../MASS/data',num2str(i),'.mat'],...
        'C3','stage','fs','D1','D2');
end
end % %.......... end of 'a0_preprocess_data.m' ...........

% % % % % % % % % % % % % % % % % % % % % % % % % % % % % % % % %
% 0.3 copy the following 5 files and save in the directory
%     get_datai_local.m
%     compTotalTP.m
%     detection2location.m
%     compCroppedSegments.m
%     compSpindles.m
% % % % % % % % % % % % % % % % % % % % % % % % % % % % % % % % %

% %........... beginning of get_datai_local.m ..............
```

```
function [C3,stage,fs,DD]=get_datai_local(i,gold_standard,datadir)
% datadir='DREAMS';or datadir='MASS';
load(['..\',datadir,'\data',num2str(i),'.mat']);%read datai
if gold_standard=='1'
    DD=D1;
elseif gold_standard=='2'
    DD=D2;
elseif gold_standard=='I'
    DD=DI;
else
    DD=DU;
end
end % %........... end of get_datai_local.m ..............

% %........ beginning of compTotalTP.m .........
function [TP]=compTotalTP(database,dnum,gold_standard)
% input: database, 'MASS' or 'DREAMS'
%        dnum, set of data# [1,2,...,n]
%        gold_standard, '1', '2', 'I', 'U'
% output: TP, total true positives
TP=0;
for i=1:length(dnum)
    load(['..\',database,'\data',num2str(dnum(i)),'.mat']);
    if gold_standard=='U'
        DD=DU;
    elseif gold_standard=='1'
        DD=D1;
    elseif gold_standard=='2'
        DD=D2;
    else
        DD=DI;
    end
    TP=TP+length(DD);
end
end % %........... end of compTotalTP.m ..............

% %.......... beginning of detection2location.m ............
function [D]=detection2location(d)
% derive head-tail pairs from a location vector of 0 and 1
len=length(d);
ix=zeros(len+2,1);
ix(2:len+1)=double(d);
tmp=ix(2:len+1)-ix(1:len);
start=find(tmp>0);
tmp=ix(2:len+1)-ix(3:len+2);
tail=find(tmp>0);
D=[start tail];
end % %.......... end of detection2location.m ............

% %............ beginning of compCroppedSegments.m ..............
function [SS,T0]=compCroppedSegments(dd,DD,len,fs,stime,seed2)
% ...subsample strategy...
% crop small segments from a whole night C3-EEG signal by including
% dd (detections by using a p0) and DD (ground truth),fs (sample
```

```
% freq),stime (sample data length in minute), seed2 (control
% randperm process)
% output: SS, heads-tails of cropped segments (total time ~ stime)
%         T0, original time length
C=zeros(len,1);
for k=1:2
    if k==1
        TT=dd;
    else
        TT=DD;
    end
    for i=1:length(TT) % expand the detected spindles to 5-s segments
        t1=TT(i,1);t2=TT(i,2); % head, tail of an event detection
        d0=max(1,floor((t2+t1)/2-2.5*fs));% begin of 5-s segments
        d1=min(len,floor(d0+5*fs));         % end of 5-s segments
        C(d0:d1)=1;
    end
end
SS=detection2location(C);
T0=sum(SS(:,2)-SS(:,1))/fs/60;% in minutes
sz1=size(SS,1);
if sz1>0 % at least one segment
    rng(seed2);
    [iSS]=randperm(sz1);
    slen=stime*60*fs;% upper sub-sample length in points
    t=0;
    for i=1:length(iSS)
        t=t+(SS(iSS(i),2)-SS(iSS(i),1));
        if t>=slen
            break;
        end
    end
    SS=SS(iSS(1:i),:);
end
end % %............ end of compCroppedSegments.m .............

% %............... beginning of compSpindles.m ................
function [dd]=compSpindles(DD,d0,d1)
% crop true spindles in DD in singal C3(d0:d1)
ix1=(DD(:,1)>=d0);
ix2=(DD(:,2)<=d1);
dd=DD(ix1&ix2,:);
end % %................ end of compSpindles.m .................

% % % % % % % % % % % % % % % % % % % % % % % % % % % % % % % %
% 0.4 copy the following c file and save in the directory
%     assessByEventJaccard_c.c
% 0.5 build MEX function by command "mex assessByEventJaccard_c.c"
%     a file assessByEventJaccard_c.mexw64 is created
% % % % % % % % % % % % % % % % % % % % % % % % % % % % % % % %

// % % ....... beginning of assessByEventJaccard_c.c .......
#include "mex.h"
//% mex assessByEventJaccard_c.c (compile c-program in MATLAB)
```

```
//% [fp,fn,tp]=assessByEventJaccard_c(detect,DD,ovlp); (MATLAB Call)
//%********** no input checking, use correct input ONLY**********
//% input: detect (Nx1) double vector, 1: spindle, 0: otherwise
//%        DD (Mx2) double array, [heads,tails] of true spindles
//%        ovlp, overlapping rate (Jaccard coefficient)
//% output: false positive/negative, true positive numbers
//% Using the by-event and JACCRAD overlap coefficient criterion
//% copyright 2016, National Central University
int dnum(int len,double *d);
void d2loc(int len, double* d,double* D31,double* D32);
double overlapJaccard(double a0,double a1,double b0,double b1);

void mexFunction(int nlhs,mxArray *plhs[],int nrhs,const mxArray
 *prhs[])
{
    double *D1,*D31,*D32,*detect;
    double *C0,*C1;
    int i,i0,i1,len,len0,len1;
    double fn,fp,tp,r,ovlp;
    mxArray *DD31,*DD32,*CC0,*CC1;
    len=(int)mxGetM(prhs[0]);//%number of rows in array
    detect=mxGetPr(prhs[0]);//%pointer to an mxArray of type double
    len0=dnum(len,detect);//%find number of detected spindles
    len1=(int)mxGetM(prhs[1]);//%get number of ground truth spindles
    D1=mxGetPr(prhs[1]);//%get heads&tails of ground truth spindles
    ovlp=*mxGetPr(prhs[2]);
    DD31=mxCreateDoubleMatrix(len0,1,mxREAL);
    D31=mxGetPr(DD31);//%heads of detected spindles
    DD32=mxCreateDoubleMatrix(len0,1,mxREAL);
    D32=mxGetPr(DD32);//%tails of detected spindles
    plhs[0]=mxCreateDoubleMatrix(1,1,mxREAL);
    plhs[1]=mxCreateDoubleMatrix(1,1,mxREAL);
    plhs[2]=mxCreateDoubleMatrix(1,1,mxREAL);
    CC0=mxCreateDoubleMatrix(len0,1,mxREAL);
    C0=mxGetPr(CC0);//%detected spindles are (true/false)
    CC1=mxCreateDoubleMatrix(len1,1,mxREAL);
    C1=mxGetPr(CC1);//%ground-truth spindles are (detected/not)
    d2loc(len,detect,D31,D32);
    for (i=0;i<len0;i++) {
        C0[i]=0;
    }
    for (i=0;i<len1;i++) {
        C1[i]=0;
    }
    if (len0>0 && len1>0) {
        i0=0;
        i1=0;
        while (i0<len0 && i1<len1) {
            r = overlapJaccard(D31[i0],D32[i0],D1[i1],D1[i1+len1]);
            if (r>ovlp) {
                C0[i0]=1;
                C1[i1]=1;
            }
            if (D32[i0]>D1[i1+len1]) {
```

```
                i1++;
            }
            else {
                i0++;
            }
        }
    }
    tp=0;
    fn=0;
    for (i=0;i<len1;i++) {
        tp+=C1[i];
        if (C1[i]<1) {
            fn+=1;
        }
    }
    fp=0;
    for (i=0;i<len0;i++) {
        if (C0[i]<1) {
            fp+=1;
        }
    }
    *mxGetPr(plhs[0])=fp;
    *mxGetPr(plhs[1])=fn;
    *mxGetPr(plhs[2])=tp;
}

int dnum(int len,double* d)
{
    int i,count,flag;
    flag=0;
    count=0;
    for (i=0;i<len;i++) {
        switch (flag) {
            case 0:
                if (d[i]>0) {
                    flag=1;
                    count++;
                }
                break;
            case 1:
                if (d[i]<1) {
                    flag=0;
                }
                break;
            default:
                break;
        }
    }
    return count;
}

void d2loc(int len,double* d,double* D31,double* D32)
{
    int i,i0,count,flag;
```

```
    flag=0;
    count=0;
    for (i=0;i<len;i++) {
        switch (flag) {
            case 0:
                if (d[i]>0) {
                    i0=i;
                    flag=1;
                }
                break;
            case 1:
                if (d[i]<1) {
                    D31[count]=i0+1;//%index from C to matlab
                    D32[count]=i;
                    count++;
                    flag=0;
                }
                break;
            default:
                break;
        }
    }
    if (flag>0) {
        D31[count]=i0+1;
        D32[count]=len;
    }
}

double overlapJaccard(double a0,double a1,double b0,double b1)
{
    double r,mn0,mx0,mn1,mx1;
    if (a0<=b0) {
        mn0=a0;
        mx0=b0;
    }
    else {
        mn0=b0;
        mx0=a0;
    }
    if (a1>=b1) {
        mx1=a1;
        mn1=b1;
    }
    else {
        mx1=b1;
        mn1=a1;
    }
    if (mn1>=mx0) {
        r=(mn1-mx0+1)/(mx1-mn0+1);
    }
    else {
        r=0;
    }
    return r;
```

```
} //% % ....... end of assessByEventJaccard_c.c ............

% % % % % % % % % % % % % % % % % % % % % % % % % % % % % % % %
% 0.6 prepare code for training/testing detectors
%     copy the following 4 files and save in the directory
%     a0_run_example.m
%     a0_train_DREAMS.m
%     a0_train_test_MASS.m
%     a0_demo_moea.m
% % % % % % % % % % % % % % % % % % % % % % % % % % % % % % % %

% % ........ beginning of a0_run_example.m .........
function a0_run_example(anum,g_std2,stime,FLAG)
% This is an example code shows how to train/test a1-a9 algorithms
% Example: a0_run_example(1,'1',60,1); a0_run_example(9,'2');
% input: anum, the detector number (1 to 9)
%        g_std2, ('1' or '2') choosing gold_standard for MASS data
%        stime, (default 60) cropped subsample in minutes
%        FLAG, (>0) compute p0, otherwise, use preprocessed results
% output: figures of Pareto fronts and PR curves
%         detailed results saved to files such as
%         'Figure1_a#_gstdU.mat'
%         'Figure2_a#_gstd#_stime##.mat'
if nargin<4
    FLAG=0;
end
if FLAG>0 % re-train alg with DREAMS database to get p0
    g_std1='U';% union of scorers 1&2 as the gold standard
    [p0]=a0_train_DREAMS(anum,g_std1);% train alg with DREAMS data
else % Or using preprocessed (max F2) results to save time
    P0{1}=[1.1871 4.2128 0.5612 2.9934];
    P0{2}=[0.1334 0.4884 1.1999 0.4663 2.8647];
    P0{3}=[89.6995 0.4847 2.6493];
    P0{4}=[11.9977 14.6945 0.2430 0.2193 0.0709 0.1589 0.8348 ...
        0.1271 0.3791 0.3045 0.1542 0.4662 0.4990 0.3749 2.3920];
    P0{5}=[9.9925 10.3175 11.9280 17.0701 3.3169 10.8780 ...
        4.7887 3.9063 0.5212 0.0501 0.3767 2.5441];
    P0{6}=[9.6618 19.2805 118.7401 12.7849 2.4261 13.4997 ...
        1.0029 0.1130 0.3146 0.0501 0.3008 2.9974];
    P0{7}=[7.1586 13.4217 90.4234 9.5959 2.7653 12.2352 ...
        1.8697 1.7345 0.4930 0.0507 0.3290 2.9867 ...
           0.0501 0.4880 0.9379 0.5592 2.3588];
    P0{8}=[0.0504 0.4964 1.2488 0.3099 2.8456 ...
           9.4912 10.0983 82.1434 28.5410 2.1315 10.9554 ...
           2.7704 2.1566 0.5155 0.0503 0.4227 2.5302];
    P0{9}=[11.8447 15.2971 0.2182 0.2172 0.0330 0.2340 0.3047 ...
        0.1053 0.4693 0.4490 0.0857 0.4999 0.2041 0.3001 0.3086 ...
           9.4624 18.3195 31.4160 46.5177 2.0060 12.0655 ...
           1.5433 1.4993  0.3935 0.0846 0.3011 2.0749];
    p0=P0{anum};
end
% To get a set of 'adequate' training sample segments from long data
% use the initial parameters p0 to crop segments for computational
% efficiency, stime controls the total length (in minutes) of
```

```
% extracted segments from each 'MASS' whole-night C3 signal.
if nargin<2
    g_std2='1';% or '2' which scorer as the gold standard
end
if nargin<3
    stime=60;% cropped signal's total time for each C3 data
end
% train/test with half/half MASS
a0_train_test_MASS(anum,g_std2,p0,stime);
end % % .......... end of 'a0_run_example.m' ............

% % ........ a0_train_DREAMS.m .........
function [p0]=a0_train_DREAMS(anum,g_std)
% Train alg_name{anum}, show the Pareto front (FP,FN) and its PR curve
% for 'DREAMS' database
% input: anum, (1~9) alg number. == detectors d1-d9
%        g_std, '1' scorer 1; '2' for scorer 2 as gold standard
%               'U' union of 1&2, 'I' intersection of 1&2
% output: p0, operating parameters that generates max F2-score
%         save the results to a file (see the code in the end)
alg_name={'a1_ferrarelli','a2_moelle','a3_martin','a4_tsanas',...
    'a5_causa','a6_huang','a7_huang_moelle','a8_moelle_huang',...
    'a9_tsanas_huang'};
PSZ=[100 100 100 150 120 120 170 170 270];% population size a1-a9
GEN=[100 100 100 250 200 200 250 250 320];% generation for a1-a9
if g_std=='1' || g_std=='U' % scorer 1 or union
    trains=[1 2 3 4 5 6 7 8];
else % '2' or 'I' scorer 2 or intersection
    trains=[1 2 3 4 5 6];
end
ovlp=0.2;% overlap rate with true spindle (JACCARD coefficient)
database='DREAMS';
PN=compTotalTP(database,trains,g_std);%total true positives (gstd)
tic,
[genes,fitness] = a0_demo_moea(alg_name{anum},ovlp,database,...
    trains,g_std,PSZ(anum),GEN(anum),1);% call spea2
ttrain=toc;
disp([alg_name{anum},' training time: ',num2str(ttrain,4),...
    ' seconds']);
FP=fitness(:,1);FN=fitness(:,2);
figure,plot(FP,FN,'r.');%draw Pareto front of (FP,FN)
TP=PN-FN;
P=TP./(TP+FP);R=TP./(TP+FN);
figure,plot(R,P,'r.');%draw PR curve
hold on,plot([0 1],[0 1],'k+');%draw corner points at [0 0] & [1 1]
hold off,xlabel('recall');ylabel('precision');axis square;
beta=2;F2=(1+beta^2)*(P.*R)./(beta^2.*P+R);
[~,k]=max(F2);%find index to max F2
p0=genes(k,:);%p0 that generates max F2
save(['Figure1_a',num2str(anum),'_gstd',g_std,'.mat'],'database',...
    'g_std','trains','genes','fitness','P','R','p0','ttrain');
end % % ............. end of a0_train_DREAMS.m .............

% % ........ a0_train_test_MASS.m .........
```

```
% Train/test alg with MASS database using a holdout design
function a0_train_test_MASS(anum,g_std,p0,stime)
% Train & test alg_name{anum}, show the resultant Pareto fronts and PR
% curves by using the 'MASS' database
% input: anum, (1~9) alg number
%        g_std, '1' scorer 1; '2' for scorer as gold standard
%        p0, parameters used to generate false positives for training
%        stime, (default 60) subsample data in minutes for training
% output: save results to a file (see the code in the end)
alg_name={'a1_ferrarelli','a2_moelle','a3_martin','a4_tsanas',...
    'a5_causa','a6_huang','a7_huang_moelle','a8_moelle_huang',...
    'a9_tsanas_huang'};
PSZ=[100 100 100 150 120 120 170 170 270];% population size a1-a9
GEN=[100 100 100 250 200 200 250 250 320];% generations for a1-a9
if g_std=='1'% scorer 1
    trains=[1 2 3 4 5 6 7 8 9];
    tests=[10 11 12 13 14 15 16 17 18 19];
else % '2'
    trains=[1 2 3   5 6 7   9];% 4,8 not scored
    tests=[10 11 12 13 14       17 18 19];% 15,16 not scored
end
ovlp=0.2;% overlap rate with true spindle (JACCARD coefficient)
database='MASS';
if nargin<4
    stime=60;% total segments are 60 minutes long
end
seed1=1;% control spea2's randomization
seed2=1;% control the randomization for selecting subsample
tic,%train with 60-minute-long per data listed in 'trains'
% run SPEA2
[genes,fitness] = a0_demo_moea(alg_name{anum},ovlp,database,trains,...
    g_std,PSZ(anum),GEN(anum),seed1,p0,stime,seed2);
ttrain=toc;%keep training compuation time
disp([alg_name{anum},' training time: ',num2str(ttrain,4),...
    ' seconds']);
% test with the whole-night data in 'trains'
[Pi,Ri,F1i]=feval([alg_name{anum},'_test'],database,trains,...
    genes,ovlp,g_std);
% test with the whole-night data in 'tests'
[Pj,Rj,F1j]=feval([alg_name{anum},'_test'],database,tests,...
    genes,ovlp,g_std);
figure,% Pareto front of (FP,FN)
plot(fitness(:,1),fitness(:,2),'r.');xlabel('FP');ylabel('FN');
% train-test paired PR curve plot
figure,hold on,
for i=1:length(Ri)
    plot([Ri(i) Rj(i)],[Pi(i) Pj(i)],'c');
end
plot(Ri,Pi,'r.',Rj,Pj,'bs');
plot([0 1],[0 1],'k+');%draw corner points at [0 0] and [1 1]
hold off,
xlabel('recall (red:train, blue:test)');ylabel('precision');
axis square;
save(['Figure2_a',num2str(anum),'_gstd',g_std,'_stime',...
```

```
    num2str(stime),'.mat'],'database','g_std','p0','trains',...
    'tests','genes','fitness','ttrain','Pi','Ri','F1i','Pj',...
    'Rj','F1j');
end % % ........... end of a0_train_test_MASS.m ............

% % ........ a0_demo_moea.m .........
% % % Interface to run SPEA2 % % %
function [genes,fitness]=a0_demo_moea(alg_name,ovlp,database,...
    trains,g_std,psz,gen,seed,p0,stime,seed2)
% input: alg_name, name of algorithm
%        ovlp, using by-event criterion with JACCARD overlap coef
%        database, 'DREAMS' or 'MASS'
%        trains, [1 2 3 ...] set of training data's numbers
%        g_std, which gold_stand to use, '1' expert1 '2' expert2,
%               'I' intersection, 'U' union of experts1,2
%        psz, population size of parameters in SPEA2
%        gen, max (evolution) generations in SPEA2
%        seed, for SPEA2's (control random number generation)
%        p0 (1xN), if given, extract segments for training (by p0)
%          if not, the whole data is used (for short DREAMS data)
%        stime, (minute) the time length of subsample for training
%        seed2, control random subsample selection for result
%               reproducibility
% output: genes, (psz x N) 'psz' sets of N optimized parameters
%         fitness, (psz x 2) optimal false positive/negative pairs
global opt;
global bounds;
% .....step1 init moea.....
disp('moea init...');
opt = moea();% Get the default option of spea2 moea
% Set the number of generations, populations, seed for spea2
opt.MaxGen = gen; % (10~20) times number-of-parameter
opt.PopSize= psz; % (10~20) times number-of-parameter
opt.Seed = seed; % default value: 1 (control spea2 randomization
                 % for result reproducibility if by the same seed)
opt.Dim = 2; % objective dimension 2 [FP FN]
% Initialize moea parameter-searching boundaries
feval([alg_name,'_init_bounds']);
% .....step2 init data.....
disp('data init...');% Initialize training data
if nargin==8 % for short data (using all data)
    feval([alg_name,'_init_data'],ovlp,database,trains,g_std);
elseif nargin==11  % for large data (using segments detected by p0)
    feval([alg_name,'_init_data'],ovlp,database,trains,g_std,...
        p0,stime,seed2);
else
    disp('function input argument error');
    genes=[];fitness=[];
    return;
end
% .....step3 Make the optimization run.....
disp('spea2 running...');
[genes, fitness] = moea([alg_name,'_train'], bounds, opt);
end % % ............. end of a0_demo_moea.m ..............
```

```
% % % % % % % % % % % % % % % % % % % % % % % % % % % % % % % %
% 1.1 prepare code for training detector d1
%     copy the following 7 M files and save in the directory
%     a1_ferrareli_run1.m
%     a1_ferrarelli_spindle_detection0.m
%     a1_ferrarelli_spindle_detection1.m
%     a1_ferrarelli_init_bounds.m
%     a1_ferrarelli_init_data.m
%     a1_ferrarelli_train.m
%     a1_ferrarelli_test.m
% % % % % % % % % % % % % % % % % % % % % % % % % % % % % % % %

% % ......... beginning of 'a1_ferrarelli_run1.m' ...........
function [detect]=a1_ferrarelli_run1(C3,stage,fs,p0)
% to find spindles using given parameter 'p0' for 1 data
% input: C3, (Nx1)
%        stage, (Nx1) sleep stage (refer to 'a0_preprocess_data.m'
%        fs: sample frequency
%        p0: parameter
%        p0=[3.4992 6.1048 0.8218 2.9844] by MASS scorer 1's gstd
%        p0=[2.0938 3.7034 0.8006 2.9920] by MASS scorer 2's gstd
% output: detect (Nx1) 1 for positive, 0 for negative detections
[rectD,peakD,pampD,trouD,tampD,maxD,meanD]...
    = a1_ferrarelli_spindle_detection0(C3,stage,fs);
detect = a1_ferrarelli_spindle_detection1(rectD,...
    peakD,pampD,trouD,tampD,maxD,meanD,fs,p0);
end % .......... end of 'a1_ferrarelli_run1.m' .............

% % .... beginning of a1_ferrarelli_spindle_detection0.m ....
function [RectifiedData,envelope_peaks,envelope_peaks_amp,...
    envelope_troughs,envelope_troughs_amp,ampdist_max,...
    ampnrem_mean]...
    = a1_ferrarelli_spindle_detection0(C3,stage,fs)
% Adopted from S.L. Wendt's code for finding Pareto front using SPEA2
% by Adam Huang and Min-Yin Liu, National Central University
% July 2014
%
% FERRARELLI Detect sleep spindles using the Ferrarelli algorithm.
% Ferrarelli et al. "Reduced Sleep Spindle Activity in Schizophrenia
% Patients", Am J Psychiatry 164, 2007, pp 483-492
%
% Implemented by M.Sc. Sabrina Lyngbye Wendt, July 2013
% published in S.C. Warby, S.L. Wendt et al. "Sleep-spindle detection:
% crowdsourcing and evaluating performance of experts, non-experts and
% automated methods", Nature Methods 11(4):385-392, 2014
nremsamples = find(stage<=-2); % Use data from stage S2+S3+S4
% Bandpass filter from 11-15 Hz and rectify filtered signal
C3_band = bandpass_filter_ferrarelli(C3,fs);
RectifiedData = abs(C3_band);
% Create envelope from the peaks of rectified signal
% (peaks found using zero-crossing of the derivative)
% x(2)-x(1), x(3)-x(2), ... + at increase, - at decrease
datader = diff(RectifiedData);
```

```
posder = zeros(length(datader),1);
% index of all points at which the rectified signal
posder(datader>0) = 1; % is increasing in amplitude
% -1 going from increase to decrease, 1 going from
diffder = diff(posder); % decrease to increase, 0 no change
% peak index of rectified signal
envelope_samples = find(diffder==-1)+1;
% peak amp of rectified signal
Envelope = RectifiedData(envelope_samples);
% Finds peaks of the envelope
datader = diff(Envelope);
posder = zeros(length(datader),1);
% index of all points at which the rectified signal
posder(datader>0) = 1; % is increasing in amplitude
diffder = diff(posder);
% peak index of Envelope signal
envelope_peaks = envelope_samples(find(diffder==-1)+1);
% peak amplitude of Envelope signal
envelope_peaks_amp = RectifiedData(envelope_peaks);
% Finds troughs of the envelope
% trough index of Envelope signal
envelope_troughs = envelope_samples(find(diffder==1)+1);
% peak trough of Envelope signal
envelope_troughs_amp = RectifiedData(envelope_troughs);
% Determine upper and lower thresholds
% extract samples that are in NREM stage S2+S3+S4
nrem_peaks_index=stage(envelope_peaks)<=-2;

% divide the distribution peaks of the Envelope signal in 120 bins
[counts amps] = hist(envelope_peaks_amp(nrem_peaks_index),120);
[~,maxi] = max(counts); % select the most numerous bin
ampdist_max = amps(maxi); % peak of the amplitude distribution
ampnrem_mean = mean(RectifiedData(nremsamples));
% Functions
    function out = bandpass_filter_ferrarelli(in,Fs)
    % This function creates a 12th order (if the sampling
    % frequency is 100 Hz) Chebyshev Type II bandpass filter with
    % passband between 10 and 16 Hz. The filter is -3 dB at 10.7
    % and 15 Hz. The input signal is filtered with the created
    % filter and the filtered signal is returned as output.
        Wp=[11 15]/(Fs/2);
        Ws=[10 16]/(Fs/2);
        Rp=3;
        Rs=40;
        [n, Wn]=cheb2ord(Wp,Ws,Rp,Rs);
        [bbp, abp]=cheby2(n,Rs,Wn);
        out=filtfilt(bbp, abp, in);
    end
end % % ..... end of a1_ferrarelli_spindle_detection0.m .....

% % .... beginning of a1_ferrarelli_spindle_detection1.m ....
function detection = a1_ferrarelli_spindle_detection1(...
    RectifiedData,envelope_peaks,envelope_peaks_amp,...
    envelope_troughs,envelope_troughs_amp,ampdist_max,...
```

```
    ampnrem_mean,fs,p)
% see 'a0_ferrarelli_spindle_detection0.m' for reference info
% using 4 adjustable parameters (see 'a1_ferrarelli_init_bounds.m')
% rectify filtered signal
len=length(RectifiedData);
detection = zeros(len,1);
lower_thresh_ratio=p(1);
upper_thresh_ratio=p(2);
dura0=p(3);
dura1=p(4);
% upper and lower thresholds
lower_threshold = lower_thresh_ratio*ampdist_max;
upper_threshold = upper_thresh_ratio*ampnrem_mean;
% Find where peaks are higher/lower than threshold
below_troughs = ...
    envelope_troughs(envelope_troughs_amp<lower_threshold);
above_peaks = envelope_peaks(envelope_peaks_amp>upper_threshold);
if numel(above_peaks)>=1 && numel(below_troughs)>=1
    [detection]=a1_ferrarelli_spindle_detection2_c(len,...
        below_troughs,above_peaks,dura0*fs,dura1*fs);
end
end % % ...... end of a1_ferrarelli_spindle_detection1.m .......

% % ........ beginning of a1_ferrarelli_init_bounds.m ..........
function a1_ferrarelli_init_bounds
% initialize the searching boundaries of a1's operating parameters
global bounds; % global variable for moea's accessibility
% using 4 adjustable parameters (default value)
% p(1) spindle lower boundary threshold ratio (2)
% p(2) spindle upper boundary threshold ratio (8)
% p(3) shortest duration (0.3~0.5s)
% p(4) longest duration (2~3s)
% (set the range of parameters as bounds=[min max;...])
%         p(1)   p(2)    p(3)  p(4)
bounds = [0.1 20; 0.1 30;0.3 1;0.3 3];
end % % ........ end of a1_ferrarelli_init_bounds.m ..........

% % ........ beginning of a1_ferrarelli_init_data.m ..........
function a1_ferrarelli_init_data(Ovlp,database,trains,g_std,...
    p0,stime,seed2)
% Initialization of a1's data struc for computational efficiency
% input argument description: see 'a0_demo_moea.m' for description
global ovlp;% JACCARD overlap coef for true detection grading
global rectN;% store data for segments used in ferrarelli method
global peakN;% store data for segments used in ferrarelli method
global pampN;% store data for segments used in ferrarelli method
global trouN;% store data for segments used in ferrarelli method
global tampN;% store data for segments used in ferrarelli method
global DDN;% ground truth [heads,tails] for each segment
global d0N;% beginnings of segments
global lenM;% data signal length
global maxM;% store data used in ferrarelli method
global meanM;% store data used in ferrarelli method
global fsM;% sampling frequency for each segment
```

```
global segM;% number of segments in a dataset
global DNUM;% total number of datasets
ovlp=Ovlp;
% pre-process sleep C3 dataset
DNUM=length(trains);% total number of training datasets
NUM=0;
for i=1:DNUM
    seg_no=0;
    % load training datasets
    % *******************   !! IMPORTANT !! ***********************
    % User need to provide a meaningful function to load C3 data
    % refer to 'a0_preprocess_data.m'
    [C3,stage,fs,DD]=get_datai_local(trains(i),g_std,database);
    % preprocessing datasets
    [rectD,peakD,pampD,trouD,tampD,maxD,meanD]...
        = a1_ferrarelli_spindle_detection0(C3,stage,fs);
    if nargin==4 % using the whole data as ONE segment for training
        % for short C3 data such as the 'DREAMS' database
        NUM=NUM+1;
        seg_no=seg_no+1;
        rectN{NUM}=rectD;% intermediate data used in a1
        peakN{NUM}=peakD;% intermediate data used in a1
        pampN{NUM}=pampD;% intermediate data used in a1
        trouN{NUM}=trouD;% intermediate data used in a1
        tampN{NUM}=tampD;% intermediate data used in a1
        DDN{NUM}=DD;% groundtruth [heads,tails] for the i-th data
        d0N(NUM)=1;
        ttime=length(C3)/fs/60;T0=ttime;
    elseif nargin==7 % using p0 to crop segments for long C3 data
        % for long C3 data such as the 'MASS' database
        [rectD,peakD,pampD,trouD,tampD,maxD,meanD]...
            = a1_ferrarelli_spindle_detection0(C3,stage,fs);
        detection = a1_ferrarelli_spindle_detection1(rectD,...
            peakD,pampD,trouD,tampD,maxD,meanD,fs,p0);
        dd=detection2location(detection);
        [SS,T0]=compCroppedSegments(dd,DD,length(C3),fs,stime,seed2);
        ttime=sum(SS(:,2)-SS(:,1))/fs/60;
        for j=1:size(SS,1) % cropped segments from whole C3 signal
            NUM=NUM+1;
            seg_no=seg_no+1;
            d0=SS(j,1);d1=SS(j,2); % head and tail of a segment
            rectN{NUM}=rectD(d0:d1);
            tmp=((peakD>=d0) & (peakD<=d1));
            ix=find(tmp>0);
            peakN{NUM}=peakD(ix)-d0+1;
            pampN{NUM}=pampD(ix);
            tmp=((trouD>=d0) & (trouD<=d1));
            ix=find(tmp>0);
            trouN{NUM}=trouD(ix)-d0+1;
            tampN{NUM}=tampD(ix);
            d0N(NUM)=d0;
            ss=compSpindles(DD,d0,d1);% get ground truth
            DDN{NUM}=ss-d0+1; % groundtruth local head-tail locations
        end
```

```
    end
    lenM(i)=length(C3);% signal length
    segM(i)=seg_no;% number of segments in the i-th data
    maxM(i)=maxD;  % intermediate data used in ferrarelli method
    meanM(i)=meanD;% intermediate data used in ferrarelli method
    fsM(i)=fs;% sampling frequency for the i-th data
    disp(['data',num2str(i),' ',num2str(seg_no),' segments',' ',...
        num2str(ttime,3),' out of ',num2str(T0,3),' minutes']);
end % end of for-i loop
end % % .......... end of a1_ferrarelli_init_data.m ...........

% % ......... beginning of 'a1_ferrarelli_train.m' ............
function [R]=a1_ferrarelli_train(p)
% called by spea2 moea during optimization
global ovlp; % overlap rate criterion by Jaccard coef
global rectN;% store data for segments used in ferrarelli method
global peakN;% store data for segments used in ferrarelli method
global pampN;% store data for segments used in ferrarelli method
global trouN;% store data for segments used in ferrarelli method
global tampN;% store data for segments used in ferrarelli method
global DDN; % heads tails of true spindles
global maxM;% store data used in ferrarelli method
global meanM;% store data used in ferrarelli method
global fsM;% sampling frequency for each segment
global segM;% number of segments in a dataset
global DNUM;% total number of datasets
R = [Inf, Inf];
FP=0;% false positives
FN=0;% false negatives
num=0;
for i=1:DNUM
    fs=fsM(i);
    ampdist_max=maxM(i);
    ampnrem_mean=meanM(i);
    if segM(i)==1
        num=num+1;
        detect = a1_ferrarelli_spindle_detection1(rectN{num},...
            peakN{num},pampN{num},trouN{num},tampN{num},...
            ampdist_max,ampnrem_mean,fs,p);
        [fp,fn,~]=assessByEventJaccard_c(detect,DDN{num},ovlp);
        FN=FN+fn;
        FP=FP+fp;
    else
        for j=1:segM(i)
            num=num+1;
            detect = a1_ferrarelli_spindle_detection1(rectN{num},...
                peakN{num},pampN{num},trouN{num},tampN{num},...
                ampdist_max,ampnrem_mean,fs,p);
            [fp,fn,~]=assessByEventJaccard_c(detect,DDN{num},ovlp);
            FN=FN+fn;
            FP=FP+fp;
        end
    end
end
```

```
R(1)=FP;
R(2)=FN;
end % % .......... end of 'a1_ferrarelli_train.m' ...........

% % .......... beginning of 'a1_ferrarelli_test.m' ..........
function [P,R,F1]=a1_ferrarelli_test(database,tests,genes,ovlp,g_std)
% test the parameters in 'genes' for data listed in tests
% refer to a0_train_test_MASS.m for input arguments' description
[PN]=compTotalTP(database,tests,g_std);% find total true positives
npar=size(genes,1);% number of parameter sets
FP=zeros(npar,1);% false positives by each parameter
FN=zeros(npar,1);% false negatives by each parameter
for i=1:length(tests) % set of test data
    disp(tests(i));
    % get data
    [C3,stage,fs,DD]=get_datai_local(tests(i),g_std,database);
    [rectD,peakD,pampD,trouD,tampD,maxD,meanD]...
        = a1_ferrarelli_spindle_detection0(C3,stage,fs);
    for j=1:npar
        p0=genes(j,:);
        detect = a1_ferrarelli_spindle_detection1(rectD,...
            peakD,pampD,trouD,tampD,maxD,meanD,fs,p0);
        [fp,fn,tp]=assessByEventJaccard_c(detect,DD,ovlp);
        FN(j)=FN(j)+fn;
        FP(j)=FP(j)+fp;
    end
end
TP=PN-FN;% detected true positives
P=TP./(TP+FP);% precision
R=TP./(TP+FN);% recall
F1=2*P.*R./(P+R);% F1-score
end % % .......... end of 'a1_ferrarelli_test.m' .............

% % % % % % % % % % % % % % % % % % % % % % % % % % % % % % % %
% 1.2 prepare c code for training detector d1
%     copy the following c file and save in the directory
%     a1_ferrarelli_spindle_detection2_c.c
%     build MEX function "mex a1_ferrarelli_spindle_detection2_c.c"
%     a file a1_ferrarelli_spindle_detection2_c.mexw64 is created
% % % % % % % % % % % % % % % % % % % % % % % % % % % % % % % %

// % % ... beginning of a1_ferrarelli_spindle_detection2_c.c ...
#include "mex.h"
/* [detect]=a1_ferrarelli_spindle_detection2_c(len,below_troughs,
 * above_peaks,dura0*fs,dura1*fs);*/
void mexFunction(int nlhs,mxArray *plhs[],int nrhs,const mxArray
 *prhs[])
{
    int i,j,i0,tnum,pnum,len,flag;
    double *detect,*below_troughs,*above_peaks;
    double dura0,dura1,tmp;
    len=(int)*mxGetPr(prhs[0]);
    tnum=(int)mxGetM(prhs[1]);
    below_troughs=mxGetPr(prhs[1]);
```

```
    pnum=(int)mxGetM(prhs[2]);
    above_peaks=mxGetPr(prhs[2]);
    dura0=(double)*mxGetPr(prhs[3]);
    dura1=(double)*mxGetPr(prhs[4]);
    plhs[0]=mxCreateDoubleMatrix(len,1,mxREAL);
    detect=mxGetPr(plhs[0]);
    for (i=0;i<tnum;i++){
        detect[(int)below_troughs[i]-1]=3;
    }
    for (i=0;i<pnum;i++){
        detect[(int)above_peaks[i]-1]=2;
    }
    if (tnum>0){
        flag=0;
        i0=(int)below_troughs[0]-1;
        i=i0+1;
        while (i<len){
            if (flag==0) {
                if (detect[i]>=3){
                    i0=i;
                }
                else if (detect[i]>=2){
                    flag=1;
                }
            }
            else if (flag==1) {
                if (detect[i]>=3) {
                    tmp=(double)(i-i0);
                    if (tmp>=dura0 && tmp<=dura1){
                        for (j=i0;j<=i;j++){
                            detect[j]=1;
                        }
                    }
                    flag=0;
                    i0=i;
                }
            }
            i++;
        }
        for (i=0;i<tnum;i++){
            if (detect[(int)below_troughs[i]-1]>=3)
                detect[(int)below_troughs[i]-1]=0;
        }
        for (i=0;i<pnum;i++){
            if (detect[(int)above_peaks[i]-1]>=2)
                detect[(int)above_peaks[i]-1]=0;
        }
    }
}// % % .... end of a1_ferrarelli_spindle_detection2_c.c .....

% % % % % % % % % % % % % % % % % % % % % % % % % % % % % % % %
% 1.3 train/test detector d1 with the following command
%     >> a0_run_example(1,'1',60,1);
%     input: 1, use alg 1,
```

```
%           '1' gold_standard,
%            sumsample 60 m,
%            train with DREAMS first
%     output: draw Figure 1A, Figure2G, Figure 4A,
%             save DREAMS results to 'Figure1_a1_gstdU.mat'
%             save MASS results to 'Figure2_a1_gstd1_stime60.mat'
%
%     train/test detector d1 with the following command
%     >> a0_run_example(1,'2');
%     input: 1, use alg 1,
%           '2' gold_standard,
%            sumsample 60 m (default)
%            doesn't train with DREAMS (use pre-computed p0)
%     output: draw Figure 5A,
%             save results to 'Figure2_a1_gstd2_stime60.mat'
%
%     run d1 on any C3 data with a chosen p0
% p0=[3.4992 6.1048 0.8218 2.9844]; train MASS Scorer1 G-STD
% p0=[2.0938 3.7034 0.8006 2.9920]; by Scorer2 Gold-STD
%     >> [detect]=a1_ferrarelli_run1(C3,stage,fs,p0);
%     refer to a1_ferrarelli_run1.m for details
% % % % % % % % % % % % % % % % % % % % % % % % % % % % % % % %

% % % % % % % % % % % % % % % % % % % % % % % % % % % % % % % %
% 1.4 Notes
%     The Pareto results may slightly different from Figs 4,5
%     due to different c-compilers and CPUs.
% % % % % % % % % % % % % % % % % % % % % % % % % % % % % % % %

% % % % % % % % % % % % % % % % % % % % % % % % % % % % % % % %
% 2.1 prepare code for training detector d2
%     copy the following 6 M files and save in the directory
%     a2_moelle_run1.m
%     a2_moelle_spindle_detection0.m
%     a2_moelle_init_bounds.m
%     a2_moelle_init_data.m
%     a2_moelle_train.m
%     a2_moelle_test.m
% % % % % % % % % % % % % % % % % % % % % % % % % % % % % % % %
% % ........... beginning of 'a2_moelle_run1.m' .............
function [detect]=a2_moelle_run1(C3,stage,fs,p0)
% to find spindles using given parameter 'p0' for 1 data
% input: C3, (Nx1)
%        stage, (Nx1) sleep stage (refer to 'a0_preprocess_data.m'
%        fs: sample frequency
%        p0: parameter
% output: detect (Nx1) 1 for positive, 0 for negative detections
[C3_band,std_C3band] = a2_moelle_spindle_detection0(C3,stage,fs);
bandsq=C3_band.^2;
detect=a2_moelle_spindle_detection1_c(bandsq,std_C3band,fs,p0);
end % % .............. end of 'a2_moelle_run1.m' .............

% % ...... beginning of 'a2_moelle_spindle_detection0.m' ......
function [C3_band,std_C3band] = ...
```

```
    a2_moelle_spindle_detection0(C3,stage,fs)
% MOELLE Detect sleep spindles using the Moelle algorithm.
% M. Molle et al. "Grouping of Spindle Activity during Slow
% Oscillations in Human Non-Rapid Eye Movement Sleep", J
% Neurosc 22(24), 2002, pp 10941-10947
%
% Implemented by M.Sc. Sabrina Lyngbye Wendt, July 2013
% published in S.C. Warby, S.L. Wendt et al. "Sleep-spindle
% detection: crowdsourcing and evaluating performance of experts,
% non-experts and automated methods", Nature Methods 11(4):
% 385-392, 2014
%
% by Adam Huang and Min-Yin Liu, National Central University
% July 2014
C3_band = bandpass_filter_lubeck(C3,fs);
std_C3band = std(C3_band(stage==-2));
% Functions
    function out = bandpass_filter_lubeck(in,Fs)
    % This function creates a 236th order (if the sampling
    % frequency is 100 Hz) Equiripple bandpass filter with
    % passband between 12 and 15 Hz. The filter is -3 dB at
    % 11.3 and 15.7 Hz. The input signal is filtered with the
    % created filter and the filtered signal is returned
    % as output.
        Fstop1 = 10;              % First Stopband Frequency
        Fpass1 = 11.3;            % First Passband Frequency
        Fpass2 = 15.7;            % Second Passband Frequency
        Fstop2 = 17;              % Second Stopband Frequency
        Dstop1 = 1.1220184543e-05;  % First Stopband Attenuation
        Dpass  = 0.057501127785;    % Passband Ripple
        Dstop2 = 1.1220184543e-05;  % Second Stopband Attenuation
        dens   = 20;              % Density Factor
        % Calculate the order from the parameters using FIRPMORD.
        [N, Fo, Ao, W] = ...
            firpmord([Fstop1 Fpass1 Fpass2 Fstop2]/(Fs/2),...
            [0 1 0], [Dstop1 Dpass Dstop2]);
        % Calculate the coefficients using the FIRPM function.
        b  = firpm(N, Fo, Ao, W, {dens});
        out = filtfilt(b,1,in);
    end
end % % ..... end of 'a2_moelle_spindle_detection0.m' ......

% % ......... beginning of a2_moelle_init_bounds.m .........
function a2_moelle_init_bounds
% initialize the searching boundaries of a2's operating parameters
global bounds;
% using 5 adjustable parameters (default value)
% p(1) time resolution (0.05s)
% p(2) rms window (0.1s)
% p(3) spindle upper boundary threshold ratio (1.5)
% p(4) shortest duration (0.3~0.5s)
% p(5) longest duration (3s)
% (set the range of parameters as bounds=[min max;...])
%         p(1)     p(2)     p(3)   p(4)  p(5)
```

```
bounds = [0.05 0.5;0.05 0.5;0.1 10;0.3 1;0.3 3];
end % % ........ end of a2_moelle_init_bounds.m ..........

% % ......... beginning of a2_moelle_init_data.m .........
function a2_moelle_init_data(Ovlp,database,trains,g_std,...
    p0,stime,seed2)
% Initialization of a2's data struc for computational efficiency
% input arguments: see 'a0_demo_moea.m' for description
global ovlp;% JACCARD overlap coef for true detection grading
global bandsqN;% store data used in moelle method
global DDN;% ground truth [heads,tails] for each segment
global d0N;% beginnings of segments
global lenM;% data signal length
global stdM;% store data used in moelle method
global fsM;% sampling frequency for each segment
global segM;% number of segments in a dataset
global DNUM;% total number of datasets
ovlp=Ovlp;
% pre-process sleep dataset
DNUM=length(trains);% total number of training datasets
NUM=0;
for i=1:DNUM
    seg_no=0;
    % load training datasets
    % ******************   !! IMPORTANT !! ********************
    % User need to provide a meaningful function to load C3 data
    % refer to 'a0_preprocess_data.m'
    [C3,stage,fs,DD]=get_datai_local(trains(i),g_std,database);
    % preprocessing datasets
    [C3_band,std_C3band] = ...
        a2_moelle_spindle_detection0(C3,stage,fs);
    bandsq=C3_band.^2;
    if nargin==4 % using the whole data as a segment for training
        % for short C3 data such as the 'DREAMS' database
        NUM=NUM+1;
        seg_no=seg_no+1;
        bandsqN{NUM}=bandsq;% intermediate data used in a2 method
        DDN{NUM}=DD;% groundtruth [heads,tails] for the i-th data
        d0N(NUM)=1;
        ttime=length(C3)/fs/60;T0=ttime;
    elseif nargin==7 % using p0 to crop segments for long C3 data
        % for long C3 data such as the 'MASS' database
        detection=a2_moelle_spindle_detection1_c(bandsq,...
            std_C3band,fs,p0);
        dd=detection2location(detection);
        [SS,T0]=compCroppedSegments(dd,DD,length(C3),fs,...
            stime,seed2);%crop
        ttime=sum(SS(:,2)-SS(:,1))/fs/60;
        for j=1:size(SS,1) % cropped segments from whole C3 signal
            NUM=NUM+1;
            seg_no=seg_no+1;
            d0=SS(j,1);d1=SS(j,2); % head and tail of a segment
            bandsqN{NUM}=bandsq(d0:d1);
            d0N(NUM)=d0;
```

```
            ss=compSpindles(DD,d0,d1);% get ground truth
            DDN{NUM}=ss-d0+1;%groundtruth local head-tail locations
        end
    end
    lenM(i)=length(C3);% signal length
    segM(i)=seg_no;% number of segments in the i-th data
    stdM(i)=std_C3band;%intermediate data used in a2 method
    fsM(i)=fs;% sampling frequency for the i-th data
    disp(['data',num2str(i),' ',num2str(seg_no),' segments',...
      ' ',num2str(ttime,3),' out of ',num2str(T0,3),' minutes']);
end % end of for-i loop
end % ............ end of a2_moelle_init_data.m .............

% % ........... beginning of 'a2_moelle_train.m' .............
function [R]=a2_moelle_train(p)
% called by spea2 moea during optimization
global ovlp;% JACCARD overlap coef for true detection grading
global bandsqN;% store data used in moelle method
global DDN;% ground truth [heads,tails] for each segment
global stdM;% store data used in moelle method
global fsM;% sampling frequency for each segment
global segM;% number of segments in a dataset
global DNUM;% total number of datasets
R = [Inf, Inf];
FP=0;% false positives
FN=0;% false negatives
num=0;
for i=1:DNUM
    fs=fsM(i);
    stdC3band=stdM(i);
    for j=1:segM(i)
        num=num+1;
        detect=a2_moelle_spindle_detection1_c(...
            bandsqN{num},stdC3band,fs,p);
        [fp,fn,~]=assessByEventJaccard_c(detect,DDN{num},ovlp);
        FN=FN+fn;
        FP=FP+fp;
    end
end
R(1)=FP;
R(2)=FN;
end % % ........... end of 'a2_moelle_train.m' .............

% % .......... beginning of 'a2_moelle_test.m' .............
function [P,R,F1]=a2_moelle_test(database,tests,genes,ovlp,g_std)
% test the parameters in 'genes' for data listed in tests
% refer to a0_train_test_MASS for input arguments' description
[PN]=compTotalTP(database,tests,g_std);%find total true positives
npar=size(genes,1);% number of parameter sets
FP=zeros(npar,1);% false positives by each parameter
FN=zeros(npar,1);% false negatives by each parameter
for i=1:length(tests)
    disp(tests(i));
    [C3,stage,fs,DD]=get_datai_local(tests(i),g_std,database);
```

```
    [C3_band,std_C3band]=a2_moelle_spindle_detection0(C3,stage,fs);
    bandsq=C3_band.^2;
    for j=1:npar
        p0=genes(j,:);
        detect=a2_moelle_spindle_detection1_c(bandsq,...
            std_C3band,fs,p0);
        [fp,fn,tp]=assessByEventJaccard_c(detect,DD,ovlp);
        FN(j)=FN(j)+fn;
        FP(j)=FP(j)+fp;
    end
end
TP=PN-FN;% detected true positives
P=TP./(TP+FP);% precision
R=TP./(TP+FN);% recall
F1=2*P.*R./(P+R);% F1-score
end % % ........... end of 'a2_moelle_test.m' .............

% % % % % % % % % % % % % % % % % % % % % % % % % % % % % % % %
% 2.2 prepare c code for training detector d2
%     copy the following c file and save in the directory
%     a2_moelle_spindle_detection1_c.c
%     build MEX function "mex a2_moelle_spindle_detection1_c.c"
%     a file a2_moelle_spindle_detection1_c.mexw64 is created
% % % % % % % % % % % % % % % % % % % % % % % % % % % % % % % %
//% % ..... beginning of a2_moelle_spindle_detection1_c.c .....
#include <math.h>
#include "mex.h"
/* c program using moelle spindle detection method */
/* author: Adam Huang, adamhuan@gmail.com */
/* [detect]=a2_moelle_spindle_detection1_c(C3_band_sq,std_C3band,fs,p)
 */
void mexFunction(int nlhs,mxArray *plhs[],int nrhs,const mxArray
 *prhs[])
{
    double *C3_band_sq,*p,*detect;//,*T,*RMS;
    double std_C3band,fs,window,thres,dura0,dura1,tot,tt;
    int i,j,i0,i1,len,tlen,halfwin,time_res,t,t0,t1,flag;
    len=(int)mxGetM(prhs[0]);//number of rows in array
    i=(int)mxGetN(prhs[0]);  //number of columns in array
    C3_band_sq=mxGetPr(prhs[0]);
    std_C3band=*mxGetPr(prhs[1]);
    fs=*mxGetPr(prhs[2]);
    p=mxGetPr(prhs[3]);
    plhs[0]=mxCreateDoubleMatrix(len,1,mxREAL);
    detect=mxGetPr(plhs[0]);
    time_res=(int)floor(p[0]*fs+0.5); // 0.05 seconds
    window=p[1];   // 0.1 seconds
    thres=p[2]*std_C3band;    // 1.5
    dura0=p[3]*fs;    // 0.3  seconds
    dura1=p[4]*fs;    // 3    seconds
    halfwin=(int)floor(window*fs/2+0.5);
    tlen=(int)floor((len-1-time_res)/time_res);
    t=time_res;
    flag=0;
```

```
for (i=0;i<tlen;i++){//find RMS > thres
    i0=t-halfwin;
    if (i0<0){
        i0=0;
    }
    i1=t+halfwin;
    if (i1>=len){
        i1=len-1;
    }
    tot=0;
    for (j=i0;j<=i1;j++){
        tot+=C3_band_sq[j];
    }
    if (sqrt(tot/(i1-i0+1))>thres){
        if (flag==0) {
            flag=1;
            t0=t;
            t1=t;
        }
        else {
            t1=t;
        }
    }
    else {
        if (flag>0) {
            flag=0;
            for (j=t0;j<=t1;j++){
                detect[j]=1;
            }
        }
    }
    t+=time_res;
}
if (flag>0) {
    for (j=t0;j<=t1;j++){
        detect[j]=1;
    }
}
tot=0;
for (i=0;i<len;i++){//check duration
    if (tot>0) {
        if (detect[i]>0) {
            tot+=1;
        }
        else {
            if (tot<dura0 || tot>dura1){
                for (j=i0;j<i;j++){
                    detect[j]=0;
                }
            }
            tot=0;
        }
    }
    else {
```

```
            if (detect[i]>0) {
                tot+=1;
                i0=i;
            }
        }
    }
}//% % ..... end of a2_moelle_spindle_detection1_c.c .....

% % % % % % % % % % % % % % % % % % % % % % % % % % % % % % % %
% 2.3 train/test detector d2 with the following example command
%     >> a0_run_example(2,'1',60,1); or a0_run_example(2,'1');
%     >> a0_run_example(2,'2');
%     run d2 on any C3 data with a chosen p0
%     >> [detect]=a2_moelle_run1(C3,stage,fs,p0);
% p0=[0.0504 0.0705 1.8961 0.4412 2.7958] MASS scorer 1 g-std
% p0=[0.2374 0.4927 1.3564 0.3834 2.7097] MASS scorer 2 g-std
% % % % % % % % % % % % % % % % % % % % % % % % % % % % % % % %

% % % % % % % % % % % % % % % % % % % % % % % % % % % % % % % %
% 3.1 prepare code for training detector d3
%     copy the following 6 M files and save in the directory
%     a3_martin_run1.m
%     a3_martin_spindle_detection0.m
%     a3_martin_init_bounds.m
%     a3_martin_init_data.m
%     a3_martin_train.m
%     a3_martin_test.m
% % % % % % % % % % % % % % % % % % % % % % % % % % % % % % % %
% % ............. beginning of 'a3_martin_run1.m' .............
function [detect]=a3_martin_run1(C3,stage,fs,p0)
% to find spindles using given parameter 'p0' for 1 data
% input: C3, (Nx1)
%        stage, (Nx1) sleep stage (ref to 'a0_preprocess_data.m')
%        fs: sample frequency
%        p0: parameter
% output: detect (Nx1) 1 for positive, 0 for negative detections
p1=0.1;% time resolution (fixed at 0.1s, 25 points for fs=250Hz)
p2=0.25;% rms window (fixed at 0.25s)
[C3band,C3nrem,dT] = a3_martin_spindle_detection0(C3,stage,fs);
C3bandsq=C3band.^2;
C3nremsq=C3nrem.^2;
p=[p1 p2 p0];
percentile=p(3);
[RMS]=a3_rms_C3nrem234_c(C3nremsq,dT,fs,p(1),p(2));
threshold = prctile(RMS,percentile);
detect = a3_montreal_c(C3bandsq,fs,threshold,p);
end % % ........... end of 'a3_martin_run1.m' ..............

% % ....... beginning of a3_martin_spindle_detection0.m ......
function [C3_band,C3nrem,dT] = ...
    a3_martin_spindle_detection0(C3,stage,fs)
% MARTIN_SPINDLE_DETECTION Detect sleep spindles using the Martin
% algorithm. N. Martin et al. "Topography of age-related changes
% in sleep spindles", Neurobio Aging 34(2), 2013, pp 468-476
```

```
%
% Implemented by M.Sc. Sabrina Lyngbye Wendt, July 2013
% published in S.C. Warby, S.L. Wendt et al. "Sleep-spindle
% detection: crowdsourcing and evaluating performance of
% experts, non-experts and automated methods", Nature Methods
% 11(4):385-392, 2014
%
% Adopted from S.L. Wendt's code for finding Pareto front using SPEA2
% Adam Huang and Min-Yin Liu, National Central University
% July 2014
IX=(stage<=-2);% index to signals at sleep stages 2,3,4
[m,n]=size(IX);
if m<n
    IX=IX';
end
ix=double(IX);
ix=[0;ix;0];
head=find((ix(2:end-1)-ix(1:end-2))>0);
tail=find((ix(2:end-1)-ix(3:end))>0);
C3_N234=cell(length(head),1);
for i=1:length(head)
    C3_N234{i}=C3(head(i):tail(i));
end
C3_band = bandpass_filter_montreal(C3,fs);
[C3nrem,dT] = threshold_montreal_part0(C3_N234,fs);

    function out = bandpass_filter_montreal(in,Fs)
    % BANDPASS_FILTER_MONTREAL Bandpass filter used in Martin
    % spindle detection. This function creates a 1023rd order
    % (if the sampling frequency is 100 Hz) Rectangular bandpass
    % filter with passband between 11.5 and 14.5 Hz. The filter
    % is -3 dB at 11.1 and 14.9 Hz. The input signal is filtered
    % with the filter and the filtered signal is returned as
    % output.
        N    = 1023;     % Order
        Fc1  = 11.08;    % First Cutoff Frequency
        Fc2  = 14.92;    % Second Cutoff Frequency
        flag = 'scale';  % Sampling Flag
        % Create the window vector for the design algorithm.
        win = rectwin(N+1);
        % Calculate the coefficients using the FIR1 function.
        b  = fir1(N, [Fc1 Fc2]/(Fs/2), 'bandpass', win, flag);
        out = filtfilt(b,1,in);
    end

    function [C3nrem,dT] = threshold_montreal_part0(C3_N234,fs)
        C3nrem=[];
        dT=[];
        for k = 1:length(C3_N234)
            signal = C3_N234{k};
            t_total = length(signal);
            if t_total < 3*1023+1
                samples2zeropad = 3*1023+1-t_total;
                first = round(samples2zeropad/2);
```

```
                second = samples2zeropad-first;
                signal=[zeros(first,1); signal; zeros(second,1)];
            else
                first = 0;
                second = 0;
            end
            signal_band = bandpass_filter_montreal(signal,fs);
            signal_band = signal_band(first+1:end-second);
            C3nrem=[C3nrem;signal_band];
            dT=[dT;length(signal_band)];
        end
    end
end % % ..... end of a3_martin_spindle_detection0.m .....

% % ........ beginning of a3_martin_init_bounds.m ........
function a3_martin_init_bounds
% initialize the boundaries of a3's operating parameters
global bounds;
% using 3 adjustable parameters
% p(1) spindle upper boundary threshold ratio (95 percentile)
% p(2) shortest duration (0.3~0.5s)
% p(3) longest duration (3s)
% (set the range of parameters as bounds=[min max;...])
%         p(1)   p(2)  p(3)
bounds = [0.1 99;0.3 1;0.3 3];
% time resolution (fixed at 0.1s, 25 points for fs=250Hz)
% rms window (fixed at 0.25s)
end % % ........ end of a3_martin_init_bounds.m ..........

% % ....... beginning of 'a3_martin_init_data.m' ..........
function a3_martin_init_data(Ovlp,database,trains,g_std,...
    p0,stime,seed2)
% Initialization of a3's data struc for computational efficiency
% input arguments: see a0_train_test_MASS.m for description
global ovlp;% JACCARD overlap coef for true detection grading
global C3bandsqN;% store data used in martin method
global DDN;% ground truth [heads,tails] for each segment
global d0N;% beginnings of segments
global P1;% time resolution (fixed at 0.1s)
global P2;% rms window (fixed at 0.25s)
global rmsPrcM;%rms percentile pre-computed table for stages 2,3,4
global lenM;% data signal length
global fsM;% sampling frequency for each dataset
global segM;% number of segments in each dataset
global DNUM;% total number of datasets
ovlp=Ovlp;
% pre-process sleep dataset
DNUM=length(trains);% total number of training datasets
P1=0.1;% time resolution (fixed at 0.1s, 25 points for fs=250Hz)
P2=0.25;% rms window (fixed at 0.25s)
NUM=0;
for i=1:DNUM
    seg_no=0;
    % load training datasets
```

```
    % *****************   !! IMPORTANT !! ********************
    % User need to provide a meaningful function to load C3 data
    % refer to 'a0_preprocess_data.m'
    [C3,stage,fs,DD]=get_datai_local(trains(i),g_std,database);
    % preprocessing datasets
    [C3band,C3nrem,dT] = a3_martin_spindle_detection0(C3,...
        stage,fs);
    C3bandsq=C3band.^2;
    C3nremsq=C3nrem.^2;
    [RMS]=a3_rms_C3nrem234_c(C3nremsq,dT,fs,P1,P2);
    rmsPrc=zeros(10000,1);% percentile table for 0.01 to 100.00
    for j=1:10000         % percentile table for 0.01 to 100.00
        rmsPrc(j)=prctile(RMS,j/100);
    end
    rmsPrcM{i}=rmsPrc;
    if nargin==4 % using the whole data as a segment for training
        % for small data such as the 'DREAMS' dataset
        NUM=NUM+1;
        seg_no=seg_no+1;
        C3bandsqN{NUM}=C3bandsq;% intermediate data used in a3
        DDN{NUM}=DD;% groundtruth [heads,tails] for the i-th data
        d0N(NUM)=1;
        ttime=length(C3)/fs/60;T0=ttime;
    elseif nargin==7 % using p0 to crop segments for long C3 data
        % for long C3 data such as the 'MASS' database
        percentile=p0(1);
        threshold = prctile(RMS,percentile);
        detection = a3_montreal_c(C3bandsq,fs,threshold,...
            [P1 P2 p0]);
        dd=detection2location(detection);
        [SS,T0]=compCroppedSegments(dd,DD,length(C3),fs,...
            stime,seed2);%crop
        ttime=sum(SS(:,2)-SS(:,1))/fs/60;
        for j=1:size(SS,1) % cropped segments from whole C3 signal
            NUM=NUM+1;
            seg_no=seg_no+1;
            d0=SS(j,1);d1=SS(j,2); % head and tail of a segment
            C3bandsqN{NUM}=C3bandsq(d0:d1);
            d0N(NUM)=d0;
            ss=compSpindles(DD,d0,d1);% get ground truth
            DDN{NUM}=ss-d0+1;%groundtruth local head-tail locations
        end
    end
    lenM(i)=length(C3);% signal length
    segM(i)=seg_no;% number of segments in the i-th data
    fsM(i)=fs;% sampling frequency for the i-th data
    disp(['data',num2str(i),' ',num2str(seg_no),...
        ' segments',' ',num2str(ttime,3),' out of ',...
        num2str(T0,3),' minutes']);
end % end of for-i loop
end % % ........ end of 'a3_martin_init_data.m' ..........

% % ......... beginning of 'a3_martin_train.m' .........
function [R]=a3_martin_train(p)
```

```
% called by spea2 moea during optimization
global ovlp;% JACCARD overlap coef for true detection grading
global C3bandsqN;% store data used in martin method
global DDN;% ground truth [heads,tails] for each segment
global P1;% time resolution (fixed at 0.1s)
global P2;% rms window (fixed at 0.25s)
global rmsPrcM;% rms percentile pre-computed table for stage 2
global fsM;% sampling frequency for each segment
global segM;% number of segments in a dataset
global DNUM;% total number of datasets
R = [Inf, Inf];
FP=0;% false positives
FN=0;% false negatives
num=0;
for i=1:DNUM
    fs=fsM(i);
    for j=1:segM(i)
        num=num+1;
        threshold = rmsPrcM{i}(floor(p(1)*100));
        detect = a3_montreal_c(C3bandsqN{num},fs,...
            threshold,[P1 P2 p]);
        [fp,fn,~]=assessByEventJaccard_c(detect,DDN{num},ovlp);
        FN=FN+fn;
        FP=FP+fp;
    end
end
R(1)=FP;
R(2)=FN;
end % % .......... end of 'a3_martin_train.m' .............

% % ......... beginning of 'a3_martin_test.m' ..........
function [P,R,F1]=a3_martin_test(database,tests,...
    genes,ovlp,g_std)
% test the parameters in 'genes' for data listed in tests
% refer to 'a0_demo_train_and_test.m' for input arguments'
% description
p1=0.1;% time resolution (fixed at 0.1s)
p2=0.25;% rms window (fixed at 0.25s)
[PN]=compTotalTP(database,tests,g_std);%find total true positives
npar=size(genes,1);% number of parameter sets
FP=zeros(npar,1);% false positives by each parameter
FN=zeros(npar,1);% false negatives by each parameter
for i=1:length(tests)
    disp(tests(i));
    [C3,stage,fs,DD]=get_datai_local(tests(i),g_std,database);
    [C3band,C3nrem,dT]=a3_martin_spindle_detection0(C3,stage,fs);
    C3bandsq=C3band.^2;
    C3nremsq=C3nrem.^2;
    for j=1:npar
        p0=genes(j,:);
        if length(p0)==3
            p=[p1 p2 p0];
        end
        percentile=p(3);
```

```
            [RMS]=a3_rms_C3nrem234_c(C3nremsq,dT,fs,p(1),p(2));
            threshold = prctile(RMS,percentile);
            detect = a3_montreal_c(C3bandsq,fs,threshold,p);
            [fp,fn,tp]=assessByEventJaccard_c(detect,DD,ovlp);
            FN(j)=FN(j)+fn;
            FP(j)=FP(j)+fp;
        end
    end
    TP=PN-FN;% detected true positives
    P=TP./(TP+FP);% precision
    R=TP./(TP+FN);% recall
    F1=2*P.*R./(P+R);% F1-score
    end % % ........... end of 'a3_martin_test.m' ..............

    % % % % % % % % % % % % % % % % % % % % % % % % % % % % % % % %
    % 3.2 prepare c code for training detector d3
    %     copy the following 2 c files and save in the directory
    %     a3_rms_C3nrem_c.c
    %     a3_montreal_c.c
    %     build MEX function "mex a3_rms_C3nrem_c.c"
    %     a mex file a3_rms_C3nrem_c.mexw64 is created
    %     build MEX function "mex a3_montreal_c.c"
    %     a mex file a3_montreal_c.mexw64 is created
    % % % % % % % % % % % % % % % % % % % % % % % % % % % % % % % %
    //% % ..... beginning of a3_rms_C3nrem234_c.c .......
    #include <math.h>
    #include "mex.h"
    /* [RMS]=a3_rms_C3nrem234_c(C3nrem_sq,dT,fs,resolution,window_length);
     */
    void mexFunction(int nlhs,mxArray *plhs[],int nrhs,const mxArray
     *prhs[])
    {
        double *C3nrem_sq,*dT,*RMS;
        double fs,resolution,window_length,tmp;
        int i,j,i0,i1,j0,j1,len,tlen,halfwin,tres,tot,count;
        len=(int)mxGetM(prhs[0]);//get the number of rows in array
        C3nrem_sq=mxGetPr(prhs[0]);
        tlen=(int)mxGetM(prhs[1]);
        dT=mxGetPr(prhs[1]);
        fs=*mxGetPr(prhs[2]);
        resolution=*mxGetPr(prhs[3]);
        tres=(int)floor(resolution*fs);
        window_length=*mxGetPr(prhs[4]);
        window_length=floor(window_length*fs);
        halfwin=(int)floor(window_length/2);
        tot=0;
        for (j=0;j<tlen;j++){
            count=0;
            for (i=halfwin;i<dT[j]-halfwin;i+=tres) {
                count++;
            }
            tot+=count;
        }
        plhs[0]=mxCreateDoubleMatrix(tot,1,mxREAL);
```

```
        RMS=mxGetPr(plhs[0]);
        count=0;
        i0=0;
        for (i=0;i<tlen;i++) {
            i1=i0+dT[i];
            j0=i0;
            j=j0+halfwin;
            j1=j+halfwin;
            while (j<i1 && count<tot) {
                tmp=0;
                if (j1>=i1)
                    j1=i1-1;
                for (j=j0;j<=j1;j++) {
                    tmp+=C3nrem_sq[j];
                }
                RMS[count]=sqrt(tmp/window_length);
                count++;
                j0+=tres;
                j+=tres;
                j1+=tres;
            }
            i0=i1;
        }
    } //% % ..... end of a3_rms_C3nrem234_c.c .......

    //% % ..... beginning of a3_montreal_c.c ......
    #include "mex.h"
    /* [detect]=a3_montreal_c(C3band_sq,fs,threshold,p); */
    void mexFunction(int nlhs,mxArray *plhs[],int nrhs,const mxArray
     *prhs[])
    {
        double *C3band_sq,*p,*detect;//,*T,*RMS;
        double fs,window,thres,dura0,dura1,tot;
        int i,j,i0,i1,len,tlen,halfwin,time_res,t,t0,t1,flag;
        len=(int)mxGetM(prhs[0]);//number of rows in array
        i=(int)mxGetN(prhs[0]);  //number of columns in array
        C3band_sq=mxGetPr(prhs[0]);
        fs=*mxGetPr(prhs[1]);
        thres=*mxGetPr(prhs[2]);
        p=mxGetPr(prhs[3]);
        plhs[0]=mxCreateDoubleMatrix(len,1,mxREAL);
        detect=mxGetPr(plhs[0]);
        time_res=(int)floor(p[0]*fs); // 0.05 seconds
        window=floor(p[1]*fs);   // 0.1 seconds
        halfwin=(int)floor(window/2);
        dura0=p[3]*fs;    // 0.3  seconds
        dura1=p[4]*fs;    // 3    seconds

        tlen=(int)floor((len-1)/time_res);
        flag=0;
        for (i=halfwin;i<len-halfwin;i+=time_res){//find RMS > thres
            i0=i-halfwin;
            if (i0<0){
                i0=0;
```

```
        }
        i1=i+halfwin;
        if (i1>=len){
            i1=len-1;
        }
        tot=0;
        for (j=i0;j<=i1;j++){
            tot+=C3band_sq[j];
        }
        if (sqrt(tot/window)>thres){
            if (flag==0) {
                flag=1;
                t0=i0;
                t1=i1;
            }
            else {
                t1=i1;
            }
        }
        else {
            if (flag>0) {
                i1=t1-t0+1;
                for (j=t0;j<=t1;j++){
                    detect[j]=1;
                }
                flag=0;
            }
        }
    }
    if (flag>0) {
        i1=t1-t0+1;
        for (j=t0;j<=t1;j++){
            detect[j]=1;
        }
    }
    flag=0;
    for (i=0;i<len;i++){
        if (detect[i]>0){
            if (flag<1){
                t0=i;
                flag=1;
            }
        }
        else{
            if (flag>0){
                flag=0;
                i1=i-t0;
                if (i1<dura0 || i1>dura1){
                    for(j=t0;j<i;j++){
                        detect[j]=0;
                    }
                }
            }
        }
```

```
    }
    if (flag>0){
        i1=len-t0;
        if (i1<dura0 || i1>dura1){
            for (j=t0;j<len;j++){
                detect[j]=0;
            }
        }
    }
} //% % ...... end of a3_montreal_c.c ......

% % % % % % % % % % % % % % % % % % % % % % % % % % % % % % % %
% 3.3 train/test detector d3 with the following example command
%     >> a0_run_example(3,'1',60,1); or a0_run_example(3,'1');
%     >> a0_run_example(3,'2');
%     run d3 on any C3 data with a chosen p0
%     >> [detect]=a3_martin_run1(C3,stage,fs,p0);
% p0=[94.6105 0.6509 2.0138] trained by MASS scorer1 gstd
% p0=[79.0110 0.9974 2.8316] trained by MASS scorer2 gstd
% % % % % % % % % % % % % % % % % % % % % % % % % % % % % % % %

% % % % % % % % % % % % % % % % % % % % % % % % % % % % % % % %
% 4.1 prepare code for training detector d4
%     copy the following 6 M files and save in the directory
%     a4_tsanas_run1.m
%     a4_tsanas_spindle_detection0.m
%     a4_tsanas_init_bounds.m
%     a4_tsanas_init_data.m
%     a4_tsanas_train.m
%     a4_tsanas_test.m
% % % % % % % % % % % % % % % % % % % % % % % % % % % % % % % %
% % ......... beginning of 'a4_tsanas_run1.m' ..........
function [detect]=a4_tsanas_run1(C3,fs,p0)
% to find spindles using given parameter 'p0' for 1 data
% input: C3, (Nx1) refer to 'a0_preprocess_data.m'
%        fs: sample frequency
%        p0: parameter
% output: detect ((N*100/fs)x1) 1 for positive, 0 for negative
%         the detect is reported in resampled points (N*100/fs)
K=10;% top K cwt coef, signal resampled to 100 Hz
[CoefsTop10,pseudo_freqCWT,new_fs]=...
    a4_tsanas_spindle_detection0(C3,fs,K);
p=p0;
[mini,frq1] = min(abs(pseudo_freqCWT-p(1)));
[mini,frq2] = min(abs(pseudo_freqCWT-p(2)));
[psi2,my2]=a4_tsanas_spindle_detection1_c(CoefsTop10',...
    frq2,frq1,p(3)*new_fs);
p(5:15)=p(5:15)*new_fs;
p(7)=p(7)/new_fs;
p(10)=p(10)/new_fs;
[detect]=a4_tsanas_spindle_detection2_c(my2,p(4:6));
[D]=detection2location(detect);
if size(D,1)>0
    [detect]=a4_tsanas_spindle_detection3_c(my2,p(7:15),...
```

```
        D(:,1)-1,D(:,2)-1,D(:,1));
end
end % .......... end of 'a4_tsanas_run1.m' ...........

% ..... beginning of 'a4_tsanas_spindle_detection0.m' ......
function [CoefsTop10,pseudo_freqCWT,new_fs]=...
    a4_tsanas_spindle_detection0(C3,fs,K)
    new_fs = 100; % resampling at 100Hz
    fs_original = fs;
    C3 = resample(C3, new_fs, fs_original); fs = new_fs;
    len=length(C3);
    CoefsTop10=zeros(10,len);
    wname = 'morl'; % Morlet wavelet
    % scales corresponding to pseudo-frequencies
    scales = 2:0.1:15;
    pseudo_freqCWT = scal2frq(scales,wname,1/fs);
    pseudo_freqCWT=pseudo_freqCWT(:);
    mxlen=180000;
    if len<=mxlen
        coefs = cwt(C3,scales,wname); % Apply CWT
        S = abs(coefs.*coefs); SC = 100*S./sum(S(:));
        Xcoef = SC;
        [temp1, sortIndex] = sort(Xcoef,'descend');
        CoefsTop10=sortIndex(1:K,:);
    else
        num=floor(len/mxlen);
        if len>num*mxlen+1000
            num=num+1;
        end
        for i=1:num
            if i==1
                i0=1;ii0=1;j0=1;
                i1=min(len,i*mxlen+1000);
                j1=i1;
                coefs = cwt(C3(i0:i1),scales,wname);%Apply CWT
                S = abs(coefs.*coefs); SC = 100*S./sum(S(:));
                Xcoef = SC;
                [temp1, sortIndex] = sort(Xcoef,'descend');
                CoefsTop10(:,ii0:i1)=sortIndex(1:K,j0:j1);
            else
                i0=(i-1)*mxlen+1-1000;
                ii0=(i-1)*mxlen+1;
                j0=1001;
                i1=min(len,i*mxlen+1000);
                j1=i1-i0+1;
                coefs = cwt(C3(i0:i1),scales,wname);%Apply CWT
                S = abs(coefs.*coefs); SC = 100*S./sum(S(:));
                Xcoef = SC;
                [temp1, sortIndex] = sort(Xcoef,'descend');
                CoefsTop10(:,ii0:i1)=sortIndex(1:K,j0:j1);
            end
        end
    end
end % % ... end of 'a4_tsanas_spindle_detection0.m' ...
```

```
% % ...... beginning of a4_tsanas_init_bounds.m .......
function a4_tsanas_init_bounds
% initialize the boundaries of a4's operating parameters
global bounds;
% using 15 adjustable parameters (default value)
% p(1) spindle lower frequency boundary (11 Hz)
% p(2) spindle upper frequency boundary (16 Hz)
% p(3) smoothing window (0.11 s)
% p(4) threshold (0.3)
% p(5) (0.03 s) min duration
% p(6) (0.1 s) merge gap
% p(7) strong threshold (0.7)
% p(8) (0.1 s) duration
% p(9) (0.3 s) duration
% p(10) intermediate threshold (0.6)
% p(11) (0.1 s) duration
% p(12) (0.2 s) duration
% p(13) (0.3 s)
% p(14) shortest duration (0.4 s)
% p(15) longest mergeable duration (1.5 s)
% bounds of adjustable parameters
% (set the range of parameters as bounds=[min max;...])
% p=     [11   16    0.11      0.3     0.03     0.1
%   0.7     0.1       0.3     0.6      0.1       0.2
%   0.3     0.4     1.5];
bounds = [8 12;14 18;0.05 0.25;0.1 0.8;0.01 0.1;0.05 0.25;...
    0.3 0.9;0.05 0.25;0.1 0.5;0.3 0.80;0.05 0.25;0.05 0.5;...
    0.1 0.5;0.3 1.0;0.3 3.0];
end % % ......... end of a4_tsanas_init_bounds.m .........

% % ....... beginning of a4_tsanas_init_data.m ........
function a4_tsanas_init_data(Ovlp,database,trains,g_std,...
    p0,stime,seed2)
% Initialization of a4's data struc for computational efficiency
% input argument description: see 'a0_train_test_MASS.m'
global ovlp;% JACCARD overlap coef for true detection grading
global coefN;% store data used in tsanas method
global freqM;% store data used in tsanas method
global DDN;% ground truth [heads,tails] for each segment
global d0N;% beginnings of segments
global lenM;% data signal length
global fsM;% sampling frequency for each dataset
global segM;% number of segments in each dataset
global DNUM;% total number of datasets
ovlp=Ovlp;
% pre-process sleep dataset
DNUM=length(trains);% total number of training datasets
NUM=0;
for i=1:DNUM
    seg_no=0;
    % load training datasets
    % ****************   !! IMPORTANT !! *****************
    % User need to provide a meaningful function to load C3 data
```

```
        % refer to 'a0_preprocess_data.m'
        [C3,stage,fs,DD]=get_datai_local(trains(i),g_std,database);
        % preprocessing datasets
        K=10;% top K cwt coef, signal resampled to 100 Hz
        [CoefsTop10,pseudo_freqCWT,new_fs]=...
            a4_tsanas_spindle_detection0(C3,fs,K);
        DD=floor((DD-1)*new_fs/fs)+1;
        if nargin==4 %using the whole data as a segment for training
            % for small data such as the 'DREAMS' dataset
            NUM=NUM+1;
            seg_no=seg_no+1;
            coefN{NUM}=CoefsTop10';% intermediate data used in a4
            DDN{NUM}=DD;%groundtruth [heads,tails] for the ith data
            d0N(NUM)=1;
            ttime=size(CoefsTop10,2)/new_fs/60;T0=ttime;
        elseif nargin==7 %using p0 to crop segments for long C3 data
            % for long C3 data such as the 'MASS' database
            p=p0;
            [mini,frq1] = min(abs(pseudo_freqCWT-p(1)));
            [mini,frq2] = min(abs(pseudo_freqCWT-p(2)));
            [psi2,my2]=a4_tsanas_spindle_detection1_c(...
                CoefsTop10',frq2,frq1,p(3)*new_fs);
    % P=[11 16 0.11 0.3 0.03*100 0.1*100 0.7 0.1*100 0.3*100 0.6 ...
    %    0.1*100 0.2*100 0.3*100 0.4*100 1.5*100];
    %    default parameters
            p(5:15)=p(5:15)*new_fs;
            p(7)=p(7)/new_fs;
            p(10)=p(10)/new_fs;
            [detection]=a4_tsanas_spindle_detection2_c(my2,p(4:6));
            [D]=detection2location(detection);
            if size(D,1)>0
                [detection]=a4_tsanas_spindle_detection3_c(my2,...
                    p(7:15),D(:,1)-1,D(:,2)-1,D(:,1));
            end
            dd=detection2location(detection);
            % signal length (resampled at 100 Hz)
            len=size(CoefsTop10,2);
            [SS,T0]=compCroppedSegments(dd,DD,len,new_fs,...
                stime,seed2);%crop
            ttime=sum(SS(:,2)-SS(:,1))/new_fs/60;
            for j=1:size(SS,1) %cropped segments from whole C3 signal
                NUM=NUM+1;
                seg_no=seg_no+1;
                d0=SS(j,1);d1=SS(j,2); % head and tail of a segment
                coefN{NUM}=CoefsTop10(:,d0:d1)';
                d0N(NUM)=d0;
                ss=compSpindles(DD,d0,d1);% get ground truth
                DDN{NUM}=ss-d0+1;%groundtruth local head-tail locations
            end
        end
        freqM{i}=pseudo_freqCWT;
        % signal length (resampled at 100 Hz)
        lenM(i)=size(CoefsTop10,2);
        segM(i)=seg_no;% number of segments in the i-th data
```

```
    fsM(i)=new_fs;% sampling frequency for the i-th data
    disp(['data',num2str(i),' ',num2str(seg_no),...
        ' segments',' ',num2str(ttime,3),' out of ',...
        num2str(T0,3),' minutes']);
end % end of for-i loop
end % % ........ end of 'a4_tsanas_init_data.m' .........

% % ......... beginning of 'a4_tsanas_train.m' ..........
function [R]=a4_tsanas_train(p0)
% called by spea2 moea during optimization
global ovlp;% JACCARD overlap coef for true detection grading
global coefN;% store data used in tsanas method
global freqM;% store data used in tsanas method
global DDN;% ground truth [heads,tails] for each segment
global fsM;% sampling frequency for each dataset
global segM;% number of segments in each dataset
global DNUM;% total number of datasets
R = [Inf, Inf];
FP=0;% false positives
FN=0;% false negatives
num=0;
for i=1:DNUM
    p=p0;
    fs=fsM(i);
    [mini,frq1] = min(abs(freqM{i}-p(1)));
    [mini,frq2] = min(abs(freqM{i}-p(2)));
    p(5:15)=p(5:15)*fs;
    p(7)=p(7)/fs;
    p(10)=p(10)/fs;
    for j=1:segM(i)
        num=num+1;
        [psi2,my2]=a4_tsanas_spindle_detection1_c(...
            coefN{num},frq2,frq1,p(3)*fs);
        [detect]=a4_tsanas_spindle_detection2_c(my2,p(4:6));
        [D]=detection2location(detect);
        if size(D,1)>0
            [detect]=a4_tsanas_spindle_detection3_c(my2,...
                p(7:15),D(:,1)-1,D(:,2)-1,D(:,1));
        end
        [fp,fn,~]=assessByEventJaccard_c(detect,DDN{num},ovlp);
        FN=FN+fn;
        FP=FP+fp;
    end
end
R(1)=FP;
R(2)=FN;
end % % ........ end of 'a4_tsanas_train.m' ...........

% % ........... beginning of 'a4_tsanas_test.m' ..........
function [P,R,F1]=a4_tsanas_test(database,tests,genes,ovlp,g_std)
% test the parameters in 'genes' for data listed in tests
% refer to 'a0_train_test_MASS.m' for input argument description
[PN]=compTotalTP(database,tests,g_std);%find total true positives
npar=size(genes,1);% number of parameter sets
```

```
FP=zeros(npar,1);% false positives by each parameter
FN=zeros(npar,1);% false negatives by each parameter
for i=1:length(tests)
    disp(tests(i));
    [C3,stage,fs,DD]=get_datai_local(tests(i),g_std,database);
    K=10;% top K cwt coef, signal resampled to 100 Hz
    [CoefsTop10,pseudo_freqCWT,new_fs]=...
        a4_tsanas_spindle_detection0(C3,fs,K);
    DD=floor((DD-1)*new_fs/fs)+1;
    for j=1:npar
        p0=genes(j,:);
        p=p0;
        [mini,frq1] = min(abs(pseudo_freqCWT-p(1)));
        [mini,frq2] = min(abs(pseudo_freqCWT-p(2)));
        [psi2,my2]=a4_tsanas_spindle_detection1_c(...
            CoefsTop10',frq2,frq1,p(3)*new_fs);
        p(5:15)=p(5:15)*new_fs;
        p(7)=p(7)/new_fs;
        p(10)=p(10)/new_fs;
        [detect]=a4_tsanas_spindle_detection2_c(my2,p(4:6));
        [D]=detection2location(detect);
        if size(D,1)>0
            [detect]=a4_tsanas_spindle_detection3_c(...
                my2,p(7:15),D(:,1)-1,D(:,2)-1,D(:,1));
        end
        [fp,fn,tp]=assessByEventJaccard_c(detect,DD,ovlp);
        FN(j)=FN(j)+fn;
        FP(j)=FP(j)+fp;
    end
end
TP=PN-FN;% detected true positives
P=TP./(TP+FP);% precision
R=TP./(TP+FN);% recall
F1=2*P.*R./(P+R);% F1-score
end % % ........... end of 'a4_tsanas_test.m' ...........

% % % % % % % % % % % % % % % % % % % % % % % % % % % % % % % %
% 4.2 prepare c code for training detector d4
%     copy the following 3 c files and save in the directory
%     a4_tsanas_spindle_detection1_c.c
%     a4_tsanas_spindle_detection2_c.c
%     a4_tsanas_spindle_detection3_c.c
%     build MEX function "mex fileName.c"
%     mex files fileName.mexw64 are created
% % % % % % % % % % % % % % % % % % % % % % % % % % % % % % % %
//% % ..... beginning of a4_tsanas_spindle_detection1_c.c .....
#include "mex.h"
/* [psi,my]=a4_tsanas_spindle_detection1_c(CoefsTop10,frq2,frq1,win);
 */
void mexFunction(int nlhs,mxArray *plhs[],int nrhs,const mxArray
 *prhs[])
{
    double *CoefsTop10,*Psi,*my;
    double frq1,frq2,L,win,tmp,tot;
```

```
int i,j,ix,rows,cols,halfwin;
rows=(int)mxGetM(prhs[0]);//get the number of rows in array
cols=(int)mxGetN(prhs[0]);//get the number of columns in array
frq2=*mxGetPr(prhs[1]);
frq1=*mxGetPr(prhs[2]);
win=*mxGetPr(prhs[3]);
CoefsTop10=mxGetPr(prhs[0]);
plhs[0]=mxCreateDoubleMatrix(rows,1,mxREAL);
Psi=mxGetPr(plhs[0]);
plhs[1]=mxCreateDoubleMatrix(rows,1,mxREAL);
my=mxGetPr(plhs[1]);
L=0;
for (i=1;i<=10;i++){
    L+=(1.0/(double)i);
}
for (i=0;i<rows;i++){
    tmp=0;
    for (j=0;j<10;j++){
        ix=i+j*rows;
        if (CoefsTop10[ix]>=frq2 && CoefsTop10[ix]<=frq1) {
            tmp+=(1.0/(double)(j+1));
        }
    }
    Psi[i]=tmp/L;
}
halfwin=(int)floor(win/2);
for (i=0;i<halfwin;i++){
    tmp=0;
    for (j=0;j<=i+halfwin;j++){
        tmp+=Psi[j];
    }
    tmp/=(double)(j+1);
    if (tmp>Psi[i]){
        my[i]=tmp;
    }
    else {
        my[i]=Psi[i];
    }
}
for (i=halfwin;i<rows-halfwin;i++){
    tmp=0;
    for (j=i-halfwin;j<=i+halfwin;j++){
        tmp+=Psi[j];
    }
    tmp/=(double)(2*halfwin+1);
    if (tmp>Psi[i]){
        my[i]=tmp;
    }
    else {
        my[i]=Psi[i];
    }
}
for (i=rows-halfwin;i<rows;i++){
    tmp=0;
```

```
            tot=0;
            for (j=i-halfwin;j<rows;j++){
                tmp+=Psi[j];
                tot+=1.0;
            }
            tmp/=tot;
            if (tmp>Psi[i]){
                my[i]=tmp;
            }
            else {
                my[i]=Psi[i];
            }
        }
    } //% % ..... end of a4_tsanas_spindle_detection1_c.c .....

    //% % ..... beginning of a4_tsanas_spindle_detection2_c.c .....
    #include "mex.h"
    /* [detect]=a4_tsanas_spindle_detection2_c(my,p); */
    void mexFunction(int nlhs,mxArray *plhs[],int nrhs,const mxArray
     *prhs[])
    {
        double *my,*p,*detect;
        double thres4,dur5,gap6;//parameters
        double val,dur;
        int i,j,i0,i1,i2,i3,rows,cols,flag;
        rows=(int)mxGetM(prhs[0]);//get the number of rows in array
        cols=(int)mxGetN(prhs[1]);//get the number of columns in array
        my=mxGetPr(prhs[0]);
        p=mxGetPr(prhs[1]);
        plhs[0]=mxCreateDoubleMatrix(rows,1,mxREAL);
        detect=mxGetPr(plhs[0]);

        thres4 =p[0];
        for (i=0;i<rows;i++){
            if (my[i]>thres4){
                detect[i]=1;
            }
        }

        //shortest duration too short 0.03 sec
        dur5=p[1];
        flag=0;
        for (i=0;i<rows;i++){
            if (detect[i]>0){
                if (flag==0){
                    flag=1;
                    i0=i;
                }
            }
            else {
                if (flag>0){
                    flag=0;
                    if (i-i0-1<dur5){
                        for (j=i0;j<i;j++){
```

```
                        detect[j]=0;
                    }
                }
            }
        }
    }

    gap6=p[2];
    //group gap2<=0.1sec
    flag=0;
    for (i=0;i<rows;i++){
        if (detect[i]>0){
            if (flag==0){
                flag=1;
            }
            else if (flag==2){
                if (i-i0<=gap6){
                    for (j=i0;j<i;j++){
                        detect[j]=1;
                    }
                }
                flag=1;
            }
        }
        else {
            if (flag==1){
                i0=i-1;
                flag=2;
            }
        }
    }
} //% % ..... end of a4_tsanas_spindle_detection2_c.c .....

//% % ..... beginning of a4_tsanas_spindle_detection3_c.c .....
#include "mex.h"
/* [detect]=a4_tsanas_spindle_detection3_c(my,p(7:15),
 * D(:,1)-1,D(:,2)-1,D(:,1)); */
void mexFunction(int nlhs,mxArray *plhs[],int nrhs,const mxArray
 *prhs[])
{
    double *my,*p,*detect,*D0,*D1,*T;
    double thresS7,durS8,durS9,thresW10;
    double durW11,durW12,gap13,durmn14,durmx15;
    double tmp1,tmp2;
    int i,j,i0,rows,cols,flag,len;
    rows=(int)mxGetM(prhs[0]);//get the number of rows in array
    cols=(int)mxGetN(prhs[1]);//get the number of columns in array
    my=mxGetPr(prhs[0]);
    p=mxGetPr(prhs[1]);
    len=(int)mxGetM(prhs[2]);
    D0=mxGetPr(prhs[2]);
    D1=mxGetPr(prhs[3]);
    T=mxGetPr(prhs[4]);
    plhs[0]=mxCreateDoubleMatrix(rows,1,mxREAL);
```

```
    detect=mxGetPr(plhs[0]);

//  min_spindle_duration = 0.4; max_spindle_duration = 1.5;
//  expected spindle duration in seconds
//  Now attempt to group candidate spindles which have a
//  gap further apart but where the candidate spindles
//  before and after appear to be "strong"

    thresS7=p[0];
    durS8=p[1];
    durS9=p[2];
    thresW10=p[3];
    durW11=p[4];
    durW12=p[5];
    gap13=p[6];
    durmn14=p[7];
    durmx15=p[8];

    for(i=0;i<len;i++) {
        for (j=(int)D0[i];j<=(int)D1[i];j++) {
            detect[j]=1;
        }
    }

    for (i=0;i<len-1;i++) {
        if ((D0[i+1]-D1[i] <= gap13) && (D1[i]-D0[i]+D1[i+1]-D0[i+1]
                <= durmx15)) {
            T[i]=1;
        }
        else {
            T[i]=0;
        }
    }

    for (i=0;i<len-1;i++) {
        if (T[i]>0){
            T[i]=0;
            if ((D1[i]-D0[i]>durS8 && D1[i+1]-D0[i+1]>durS9) ||
                    (D1[i]-D0[i]>durS9 && D1[i+1]-D0[i+1]>durS8)){
                tmp1=0;
                for (j=(int)D0[i];j<=(int)D1[i];j++){
                    tmp1+=my[j];
                }
                tmp1/=(double)(D1[i]-D0[i]+1);
                tmp2=0;
                for (j=(int)D0[i+1];j<=(int)D1[i+1];j++){
                    tmp2+=my[j];
                }
                tmp2/=(double)(D1[i+1]-D0[i+1]+1);
                if (tmp1>thresW10 && tmp2>thresW10){
                    T[i]=1;
                }
            }
            else if ((D1[i]-D0[i]>durW11 && D1[i+1]-D0[i+1]>durW12) ||
```

```
                    (D1[i]-D0[i]>durW12 && D1[i+1]-D0[i+1]>durW11)){
                tmp1=0;
                for (j=(int)D0[i];j<=(int)D1[i];j++){
                    tmp1+=my[j];
                }
                tmp1/=(double)(D1[i]-D0[i]+1);
                tmp2=0;
                for (j=(int)D0[i+1];j<=(int)D1[i+1];j++){
                    tmp2+=my[j];
                }
                tmp2/=(double)(D1[i+1]-D0[i+1]+1);
                if (tmp1>thresS7 && tmp2>thresS7){
                    T[i]=1;
                }
            }
        }
    }

    for (i=0;i<len-1;i++){
        if (T[i]>0) {
            for (j=(int)D1[i];j<=(int)D0[i+1];j++){
                detect[j]=1;
            }
        }
    }

    flag=0;
    for (i=0;i<rows;i++){
        if (detect[i]>0){
            if (flag==0){
                flag=1;
                i0=i;
            }
        }
        else {
            if (flag>0){
                flag=0;
                if (i-i0-1<durmn14){
                    for (j=i0;j<i;j++){
                        detect[j]=0;
                    }
                }
            }
        }
    }
} //% % .... end of a4_tsanas_spindle_detection3_c.c .....

% % % % % % % % % % % % % % % % % % % % % % % % % % % % % % % %
% 4.3 train/test detector d4 with the following example command
%     >> a0_run_example(4,'1',60,1);or a0_run_example(4,'1');
%     >> a0_run_example(4,'2');
%     run d4 on any C3 data with a chosen p0
%     >> [detect]=a4_tsanas_run1(C3,fs,p0);
% p0=[11.8479 15.3682 0.2474 0.6726 0.0698 0.0791 0.8601 0.2494
```

```
%     0.1003 0.3176 0.0504 0.4057 0.4794 0.3245 2.9207] s1-gstd
% p0=[11.9869 15.0393 0.2404 0.4427 0.0808 0.0626 0.6583 0.1902
%     0.2832 0.5567 0.0575 0.3233 0.4949 0.3492 2.9936] s2-gstd
% % % % % % % % % % % % % % % % % % % % % % % % % % % % % % % %

% % % % % % % % % % % % % % % % % % % % % % % % % % % % % % % %
% 5.1 prepare code for training detector d5
%     copy the following 7 M files and save in the directory
%     a5_causa_run1.m
%     a5_causa_spindle_detection0.m
%     a5_causa_init_bounds.m
%     a5_causa_init_data.m
%     a5_causa_train.m
%     a5_causa_test.m
%     a5_avgfreq1.m
% % % % % % % % % % % % % % % % % % % % % % % % % % % % % % % %
% % ..... beginning of a5_causa_run1.m .....
function [detect]=a5_causa_run1(C3,fs,p0)
% to find spindles using given parameter 'p0' for 1 data
% input: C3, (Nx1) refer to 'a0_preprocess_data.m'
%        fs: sample frequency
%        p0: parameter (refer to 'a5_causa_init_bounds.m')
% output: detect (Nx1) 1 for positive, 0 for negative detections
[a_imf,f_imf] = a5_causa_spindle_detection0(C3,fs);
detect = a5_causa_spindle_detection1_c(a_imf,f_imf,fs,p0);
end % % ....... end of a5_causa_run1.m .....

% % ..... beginning of a5_causa_spindle_detection0.m .....
function [a_imf,f_imf] = a5_causa_spindle_detection0(C3,fs)
    [C3_imf]=rcada_eemd(C3,0,1,4);
    [m,n]=size(C3_imf);
    m=3;
    a_imf=zeros(m,n);
    f_imf=zeros(m,n);
    for i=1:3
        [a,f]=afnht(C3_imf(i,:),1/fs);
        [avgf] = a5_avgfreq1(C3_imf(i,:),fs);
        a_imf(i,:)=a;
        for j=1:length(avgf)
            f_imf(i,j)=avgf(j);
        end
    end

    function [a,f] = afnht(vimf, Ts)
        abs_vimf=abs(vimf);
        Nnormal=5;
        dd=1:1:length(abs_vimf);
        for jj=1:Nnormal,
            [spmax, spmin]=extrema(abs_vimf);
            upper= spline(spmax(:,1),spmax(:,2),dd);
            abs_vimf=abs_vimf./(abs(upper)+0.001);
            if jj==1
                a=abs(upper);
            end
```

```
            end
            % nvimf=abs(abs_vimf);
            % theta = angle(hilbert(nvimf));
            % f=diff(theta)/Ts/(2*pi);
            % f=[f,f(end)];
            nvimf=abs(abs_vimf);
            ix=(vimf<0);
            nvimf(ix)=-nvimf(ix);
            theta = angle(hilbert(nvimf));
            f=diff(theta)/Ts/(2*pi);
            f=[f,f(end)];
        end

        function [spmax,spmin]=extrema(x)
            n=length(x);
            t=[x(1),x,x(end)];
            peak=(t(2:n+1)>=t(1:n))&(t(2:n+1)>=t(3:n+2));
            trough=(t(2:n+1)<=t(1:n))&(t(2:n+1)<=t(3:n+2));
            peak(1)=1;
            peak(n)=1;
            trough(1)=1;
            trough(n)=1;
            ix=1:n;
            spmax=[(ix(peak))',(x(peak))'];
            spmin=[(ix(trough))',(x(trough))'];
        end
    end % % ....... end of a5_causa_spindle_detection0.m .....

    % % ..... beginning of a5_causa_init_bounds.m .....
    function a5_causa_init_bounds
    % initialize the searching boundaries of a5's operating parameters
    global bounds;
    % using 12 adjustable parameters (default value)
    % p(1) fuzzy logic ramp distance below p(2)  (5uV)
    % p(2) fuzzy logic lower amplitude boundary   (15uV)
    % p(3) fuzzy logic amplitude plateau(?, upper boundary=p(2)+p(3)uV)
    % p(4) fuzzy logic ramp distance above p(3) (30 muV)
    % p(5) fuzzy logic ramp distance below p(6) (0.5 Hz)
    % p(6) fuzzy logic lower frequency boundary  (10 Hz)
    % p(7) fuzzy logic frequency plateau(6, upper boundary=p(6)+p(7)Hz)
    % p(8) fuzzy logic ramp distance above p(7) (0.5 Hz)
    % p(9) the maximum instantaneous product threshold (0.5)
    % p(10) pulse merging gap threshold (0.2s)
    % p(11) shortest duration (0.3~0.5s)
    % p(12) longest duration (3s)
    % bounds of adjustable parameters
    % (set the range of parameters as bounds=[min max;...])
    bounds = [1  10;5 40;10 120; 1 50;0.1 4;8 13.5;1 8;0.1 4;...
        0.01 0.99;0.05 0.5;0.3 1;0.3 3];
    end % % ....... end of a5_causa_init_bounds.m .....

    % % ..... beginning of a5_causa_init_data.m .....
    function a5_causa_init_data(Ovlp,database,trains,g_std,...
        p0,stime,seed2)
```

```
% Initialize of a5's data struc for computational efficiency
% input arguments: see 'a0_demo_moea.m' for description
global ovlp;% JACCARD overlap coef for true detection grading
global aimfN;% store data for segments used in causa method
global fimfN;% store data for segments used in causa method
global DDN;% ground truth [heads,tails] for each segment
global d0N;% beginnings of segments
global lenM;% data signal length
global fsM;% sampling frequency for each dataset
global segM;% number of segments in each dataset
global DNUM;% total number of datasets
ovlp=Ovlp;
% pre-process sleep dataset
DNUM=length(trains);% total number of training datasets
NUM=0;
for i=1:DNUM
    seg_no=0;
    % load training datasets
    [C3,stage,fs,DD]=get_datai_local(trains(i),g_std,database);
    % preprocessing datasets
    [a_imf,f_imf] = a5_causa_spindle_detection0(C3,fs);
    if nargin==4 % using the whole data as a segment for training
        % for small data such as the 'DREAMS' dataset
        NUM=NUM+1;
        seg_no=seg_no+1;
        aimfN{NUM}=a_imf;% intermediate data used in causa method
        fimfN{NUM}=f_imf;% intermediate data used in causa method
        DDN{NUM}=DD;% groundtruth [heads,tails] for the i-th data
        d0N(NUM)=1;
        ttime=length(C3)/fs/60;T0=ttime;
    elseif nargin==7 % using p0 to crop segments for long C3 data
        % for long C3 data such as the 'MASS' database
        detection = a5_causa_spindle_detection1_c(a_imf,...
            f_imf,fs,p0);
        % get [heads tails] of detections
        dd=detection2location(detection);
        [SS,T0]=compCroppedSegments(dd,DD,length(C3),fs,...
            stime,seed2);%crop
        ttime=sum(SS(:,2)-SS(:,1))/fs/60;
        for j=1:size(SS,1) %cropped segments from whole C3 signal
            NUM=NUM+1;
            seg_no=seg_no+1;
            d0=SS(j,1);d1=SS(j,2); % head and tail of a segment
            aimfN{NUM}=a_imf(:,d0:d1);
            fimfN{NUM}=f_imf(:,d0:d1);
            d0N(NUM)=d0;
            ss=compSpindles(DD,d0,d1);% get ground truth
            DDN{NUM}=ss-d0+1;%grnd-truth local head-tail locations
        end
    end
    lenM(i)=length(C3);% signal length
    segM(i)=seg_no;% number of segments in the i-th data
    fsM(i)=fs;% sampling frequency for the i-th data
    disp(['data',num2str(i),' ',num2str(seg_no),...
```

```
        ' segments',' ',num2str(ttime,3),' out of ',...
        num2str(T0,3),' minutes']);
end % end of for-i loop
end % % ....... end of a5_causa_init_data.m .....

% % ..... beginning of a5_causa_train.m .....
function [R]=a5_causa_train(p)
% called by spea2 moea during optimization
global ovlp;% JACCARD overlap coef for true detection grading
global aimfN;% store data used in causa method
global fimfN;% store data used in causa method
global DDN;% ground truth [heads,tails] for each segment
global fsM;% sampling frequency for each dataset
global segM;% number of segments in each dataset
global DNUM;% total number of datasets
R = [Inf, Inf];
FP=0;% false positives
FN=0;% false negatives
num=0;
for i=1:DNUM
    fs=fsM(i);
    for j=1:segM(i)
        num=num+1;
        detect = a5_causa_spindle_detection1_c(aimfN{num},...
            fimfN{num},fs,p);
        [fp,fn,~]=assessByEventJaccard_c(detect,DDN{num},ovlp);
        FN=FN+fn;
        FP=FP+fp;
    end
end
R(1)=FP;
R(2)=FN;
end % % ....... end of a5_causa_train.m .....

% % ..... beginning of a5_causa_test.m .....
function [P,R,F1]=a5_causa_test(database,tests,genes,ovlp,g_std)
% test the parameters in 'genes' for data listed in tests
% ref to a0_demo_train_and_test.m for input arg description
[PN]=compTotalTP(database,tests,g_std);% find total true positives
npar=size(genes,1);% number of parameter sets
FP=zeros(npar,1);% false positives by each parameter
FN=zeros(npar,1);% false negatives by each parameter
for i=1:length(tests)
    disp(tests(i));
    [C3,stage,fs,DD]=get_datai_local(tests(i),g_std,database);
    [a_imf,f_imf] = a5_causa_spindle_detection0(C3,fs);
    for j=1:npar
        p0=genes(j,:);
        detect = a5_causa_spindle_detection1_c(a_imf,...
            f_imf,fs,p0);
        [fp,fn,tp]=assessByEventJaccard_c(detect,DD,ovlp);
        FN(j)=FN(j)+fn;
        FP(j)=FP(j)+fp;
    end
```

```
end
TP=PN-FN;% detected true positives
P=TP./(TP+FP);% precision
R=TP./(TP+FN);% recall
F1=2*P.*R./(P+R);% F1-score
end % % ....... end of a5_causa_test.m .....

% % ..... beginning of a5_avgfreq1.m .....
function [avgf] = a5_avgfreq1(vimf,fs)
% compute frequency from 1/2 cycles from zero-crossings
len=length(vimf);
avgf=zeros(len,1);
hlf=zeros(len,1);
counter=1;
hlf(counter)=1;
if vimf(1)>=0
    flag=1;
else
    flag=0;
end
for i=2:len
    if flag>0
        if vimf(i)<0
            dt=min(1,vimf(i-1)/(vimf(i-1)-vimf(i)+0.0001));
            t1=i-1+dt; % zero-crossing
            counter=counter+1;
            hlf(counter)=t1;
            flag=0;
        end
    else
        if vimf(i)>=0
            dt=min(1,-vimf(i-1)/(vimf(i)-vimf(i-1)+0.0001));
            t1=i-1+dt; % zero-crossing
            counter=counter+1;
            hlf(counter)=t1;
            flag=1;
        end
    end
end
if t1<len
    counter=counter+1;
    hlf(counter)=len;
end
for i=1:counter-1
    i0=ceil(hlf(i));
    i1=floor(hlf(i+1));
    avgf(i0:i1)=0.5*fs/(hlf(i+1)-hlf(i));
end
end % % ....... end of a5_avgfreq1.m .....

% % % % % % % % % % % % % % % % % % % % % % % % % % % % % % % %
% 5.2 prepare c code for training detector d5
%     copy the following 1 c files and save in the directory
%     a5_causa_spindle_detection1_c.c
```

```
%     build MEX function "mex a5_causa_spindle_detection1_c.c"
%     a mex file a5_causa_spindle_detection1_c.mexw64 is created
% % % % % % % % % % % % % % % % % % % % % % % % % % % % % % % %
//% % ..... beginning of a5_causa_spindle_detection1_c.c .....
#include "mex.h"
double fuzzi(double t,double p1,double p2, double p3, double p4);
/* [detect]=a5_causa_spindle_detection1_c(a_imf,f_imf,fs,p);*/
void mexFunction(int nlhs,mxArray *plhs[],int nrhs,const mxArray
 *prhs[])
{
    int col,row,i,j,k,g,flag,j0,j1;
    double *ptr,*ptr1,*ptr2;
    double fs,p1,p2,p3,p4,p5,p6,p7,p8,p9,p10,p11,p12;
    double t,t1,t2,amp,frq;

    col=mxGetN(prhs[0]);
    row=mxGetM(prhs[0]);
    i=mxGetN(prhs[1]);
    if (i<col) {
        col=i;
    }
    i=mxGetM(prhs[1]);
    if (i<row) {
        row=i;
    }
    fs=*mxGetPr(prhs[2]);
    ptr=mxGetPr(prhs[3]);
    p1=*ptr;
    p2=*(ptr+1);
    p3=*(ptr+2);
    p4=*(ptr+3);
    p1=p2-p1;
    p3=p2+p3;
    p4=p3+p4;
    p5=*(ptr+4);
    p6=*(ptr+5);
    p7=*(ptr+6);
    p8=*(ptr+7);
    p5=p6-p5;
    p7=p6+p7;
    p8=p7+p8;
    p9=*(ptr+8);
    p10=*(ptr+9);
    p11=*(ptr+10);
    p12=*(ptr+11);
    p10=p10*fs;
    p11=p11*fs;
    p12=p12*fs;
    plhs[0]=mxCreateDoubleMatrix(col,1,mxREAL);
    ptr =mxGetPr(plhs[0]);
    ptr1=mxGetPr(prhs[0]);
    ptr2=mxGetPr(prhs[1]);
    for (j=0;j<col;j++) {
        t=0;
```

```
        g=row*j;
        for (i=0;i<row;i++) {
            k=g+i;
            amp=ptr1[k];
            t1=fuzzi(amp,p1,p2,p3,p4);
            frq=ptr2[k];
            t2=fuzzi(frq,p5,p6,p7,p8);
            t1*=t2;
            if (t1 > t) {
                t=t1;
            }
        }
        if (t>=p9) {
            ptr[j]=1;
        }
        else {
            ptr[j]=0;
        }
    }
    flag=0;//merge
    for (j=0;j<col;j++) {
        switch (flag) {
            case 0:
                if (ptr[j]>0) {
                    j0=j;
                    flag=1;
                }
                break;
            case 1:
                if (ptr[j]>0) {
                    j0=j;
                }
                else {
                    flag=2;
                }
                break;
            case 2:
                if (ptr[j]>0) {
                    j1=j;
                    if (j1-j0 < p10) {
                        for (i=j0+1;i<j1;i++) {
                            ptr[i]=1;
                        }
                    }
                    j0=j;
                    flag=1;
                }
                break;
            default:
                break;
        }
    }
    flag=0;//duration check
    for (j=0;j<col;j++) {
```

```
            switch (flag) {
                case 0:
                    if (ptr[j]>0) {
                        j0=j;
                        flag=1;
                    }
                    break;
                case 1:
                    if (ptr[j]<1) {
                        j1=j;
                        flag=0;
                        if ((j1-j0 < p11) || (j1-j0 > p12)) {
                            for (i=j0;i<j1;i++) {
                                ptr[i]=0;
                            }
                        }
                    }
                    break;
                default:
                    break;
            }
        }
        if (flag>0) {
            if ((col-j0 < p11) || (col-j0 > p12)) {
                for (i=j0;i<col;i++) {
                    ptr[i]=0;
                }
            }
        }
    }

    double fuzzi(double t,double p1,double p2, double p3, double p4)
    {
        double p;
        if ((t<=p1) || (t>=p4)) {
            p=0;
        }
        else {
            if (t<p2) {
                p=(t-p1)/(p2-p1);
            }
            else if (t<=p3) {
                p=1;
            }
            else {
                p=(p4-t)/(p4-p3);
            }
        }
        return p;
    }//% % ....... end of a5_causa_spindle_detection1_c.c .....

    % % % % % % % % % % % % % % % % % % % % % % % % % % % % % % % %
    % 5.3 EMD code preparation
    %     download 'emd.mexw64' at:
```

```
%     http://rcada.ncu.edu.tw/FEEMD.rar
%     accessed 2016/11/21
%     COPY the following 'rcada_eemd.m' to the directory
% % % % % % % % % % % % % % % % % % % % % % % % % % % % % % %
% % ........ beginning of rcada_eemd.m .....
function [allmode] = rcada_eemd(Y,NoiseLevel,NE,numImf,varargin)
% interface to call fast EMD/EEMD/CEEMD code:
%  Copyright (C) RCADA, National Central University; 2013
%  ***** For Educational and Academic Purposes Only ******
%  Author : Yung-Hung Wang, RCADA, NCU
%
% Please Cite:
% Y. H. Wang, C. H. Yeh, H. W. V. Young, K. Hu and M. T. Lo,
% "On the computational complexity of the empirical mode
% decomposition algorithm"
% Physica A: Statistical Mechanics and its Applications,
% vol. 400,Issue 15, pp. 159-167, 2014
%
% Usage:
% (A) [allmode]=rcada_eemd(Y, NoiseLevel, NE, numImf)
% (B) [allmode]=rcada_eemd(x,NoiseLevel,NE,numImf,runCEEMD,...
% maxSift,typeSpline,toModifyBC,randType,seedNo,checkInput);
%----------------------------------------------------------------
% INPUTS :
%        Y: input signal
%        NoiseLevel: noise level
%        NE: ensemble number; if NE=1 and NoiseLevel = 0,
%            then run EMD [2]
%        numImf: number of prescribed imf; if it is less
%            than zero,it will be log2(n), n=data length
%
%-------------Additional Input Properties------------------
% runCEEMD: default=0; 0: run EEMD [3]; 1: CEEMD [4], add
% anti-phase noise, so input signal is reconstructed;
% maxSift:default = 10; sifting iteration number;
% typeSpline: default=2; 1: clamped spline; 2: not a knot
% spline; 3: natural cubic spline;
% toModifyBC: default=1; 0: None ; 1: modified linear
% extrapolation; 2: Mirror Boundary
% randType: fefault=2; 1: uniformly distributed random
% noise; 2: gaussian white noise
% seedNo: default=1; random seed number in generating
% white noise; The seed number must be an integer between
% 0 and 2^32 - 1
% checkSignal: default=1;  0=> dont check  the input
% signal;  1: check there is any  NaN or Infinity in input;
%
% OUTPUTS :
%  allmode[m][t]: returned imfs; m=imf index, t=time index

fprintf('Copyright (C) RCADA, NCU, Taiwan.\n');
allmode = [];
%verifyCode = 20041017; % For verification of emd
[Y,NoiseLevel,NE,numImf,runCEEMD,maxSift,typeSpline,...
```

```
    toModifyBC,randType,seedNo,IsInputOkay] = ...
    parse_checkProperty(Y, NoiseLevel, NE, numImf, varargin);

if(~IsInputOkay)
    fprintf('ERROR : The process is not executed.\n');
    return;
end

if (NoiseLevel == 0)
    allmode = emd(Y, toModifyBC, typeSpline, numImf, maxSift);
    allmode = allmode';
    return;
end

xsize = size(Y,2);
Ystd = std(Y);

allmode = zeros(xsize,numImf);

savedState = set_seed(seedNo);
if (runCEEMD)
   % YHW0202_2011: flip noise to balance the perturbed noise
   NE = 2*NE;
end

for iii=1:NE  % ensemble loop
    if (runCEEMD)
        if (mod(iii,2) ~= 0)
            if (randType == 1) % White Noise
               temp = ((2*rand(1,xsize)-1)*NoiseLevel).*Ystd;
            elseif (randType == 2) % Gaussian Noise
               temp = (randn(1,xsize)*NoiseLevel).*Ystd;
            end
         else % Even number
           temp = -temp;
        end
    else % runCEEMD = 0
        if (randType == 1)
            % temp is Ystd*[0 1]
            temp = (2*rand(1,xsize)-1)*NoiseLevel.*Ystd;
        elseif (randType == 2)
            % temp is Ystd*[0 1]
            temp = randn(1,xsize)*NoiseLevel.*Ystd;
        end
    end
    xend =  Y + temp;
    imf = emd(xend, toModifyBC, typeSpline, numImf, maxSift);
    allmode = allmode + imf;
end % iii: ensemble loop

return_seed(savedState);
allmode = allmode/NE;

allmode = allmode'; % 0318_2014
```

```
return; % end eemd

end

function savedState = set_seed(seedNo)
defaultStream = RandStream.getGlobalStream;
savedState = defaultStream.State;
rand('seed',seedNo);
randn('seed',seedNo);

end

function return_seed(savedState)
RandStream.getDefaultStream.State = savedState;
end

function [Y, NoiseLevel, NE, numImf, runCEEMD, maxSift,...
    typeSpline,toModifyBC,randType,seedNo, IsInputOkay] =...
    parse_checkProperty(Y, NoiseLevel, NE, numImf, varargin)
% Default Parameters
runCEEMD = 0; % Original EEMD
maxSift = 10; % maxSift = 10
typeSpline = 2;
toModifyBC = 1;
randType = 2;
seedNo = 1; % now
checkSignal = 0;
IsInputOkay = true;

if(~isempty(varargin{1}))

for iArg = 1 : length(varargin{1});

if(iArg == 1)
   runCEEMD = varargin{1}{iArg};
   if(runCEEMD ~= 0 && runCEEMD ~= 1)
    fprintf('ERROR : runCEEMD must be 0 (Off) or 1 (On).\n');
    IsInputOkay = false;
    return;
   end
end
if(iArg == 2)
   maxSift = varargin{1}{iArg};
   if(maxSift < 1 || (mod(maxSift, 1) ~= 0))
    txt=['ERROR : Number of Iteration must be an',...
        ' integer more than 0.\n'];
    fprintf(txt);
    IsInputOkay = false;
    return;
   end
end
if(iArg == 3)
    typeSpline = varargin{1}{iArg};
    if(typeSpline ~= 1 && typeSpline ~= 2 && typeSpline ~= 3)
```

```
        txt=['ERROR : typeSpline must be 1 (clamped spline);'...
            ' 2 (not a knot spline).\n'];
        fprintf(txt);
        IsInputOkay = false;
        return;
        end
    end
    if(iArg == 4)
        toModifyBC = varargin{1}{iArg};
        if(toModifyBC ~= 0 && toModifyBC ~= 1 && toModifyBC ~= 2)
        txt=['ERROR : toModifyBC must be 0 (None) ; 1',...
            ' (modified linear extrapolation); 2',...
            ' (Mirror Boundary)\n'];
        fprintf(txt);
        IsInputOkay = false;
        return;
        end
    end
    if(iArg == 5)
        randType = varargin{1}{iArg};
        if(randType ~= 1 && randType ~= 2)
        txt=['ERROR : randType must be 1 (uniformly',...
            ' distributed white noise) ; 2 (gaussian',...
            ' white noise).\n'];
        fprintf(txt);
        IsInputOkay = false;
        return;
        end
    end
    if(iArg == 6)
        seedNo = varargin{1}{iArg};
        if(seedNo < 0 || seedNo >  2^32-1 || (mod(seedNo, 1) ~= 0))
        txt=['ERROR : The value of seed must be an integer',...
            ' between 0 and 2^32 - 1. \n'];
        fprintf(txt);
        IsInputOkay = false;
        return;
        end
    end
    if(iArg == 7)
        checkSignal = varargin{1}{iArg};
        if(checkSignal ~= 0 && checkSignal ~= 1)
        txt=['ERROR : Number of checksignal must be 1',...
            ' (Yes) or 0 (No).\n'];
        fprintf(txt);
        IsInputOkay = false;
        return;
        end
    end

    end

    end
```

```
if(NoiseLevel == 0)
    fprintf('If NoiseLevel is ZERO, run EMD algorithm.\n');
end
if ((NE < 1) || (mod(NE, 1) ~= 0))
    fprintf('ERROR : Number of Ensemble must > 0.\n');
    IsInputOkay = false;
    return;
end


[m,n] = size(Y);
if(m ~= 1)
    if((n ~= 1))
       fprintf('ERROR : EMD could not input matrix array !\n');
       IsInputOkay = false;
       return;
    else
        Y =Y';
        xsize = m;
    end
else
    xsize = n;
end

if (checkSignal == 1)
    if((any(isinf(Y(:)) == 1)) || (any(isnan(Y(:)) == 1)))
        txt=['ERROR : The input signal has NaN or',...
            ' Infinity elements.\n'];
        fprintf(txt);
        IsInputOkay = false;
        return;
    end
end

if(mod(numImf, 1) ~= 0)
    txt=['ERROR : numImf must be an integer more than 0. \n'];
    fprintf(txt);
    IsInputOkay = false;
    return;
end

if (numImf <= 0) % automatic estimating number of imf
    numImf=fix(log2(xsize));
end

end
% % ........ end of rcada_eemd.m .....

% % % % % % % % % % % % % % % % % % % % % % % % % % % % % % % %
% 5.4 train/test detector d5 with the following command
%     >> a0_run_example(5,'1',60,1);or a0_run_example(5,'1');
%     >> a0_run_example(5,'2');
%     run d5 on any C3 data with a chosen p0
%     >> [detect]=a5_causa_run1(C3,fs,p0);
```

```
% p0=[6.5427 13.4609 38.4767 42.5246 3.5135 12.38 2.7942 1.6175
%     0.1657 0.05 0.4899 2.5457] s1-gtsd
% p0=[2.9939 6.0575 74.9148 39.3272 3.9688 13.4238 1.0034 2.7247
%     0.0363 0.0503 0.7581 2.9536] s2-gstd
% % % % % % % % % % % % % % % % % % % % % % % % % % % % % % % %

% % % % % % % % % % % % % % % % % % % % % % % % % % % % % % % %
% 6.1 prepare code for training/testing detector d6
%     copy the following 10 M files and save in the directory
%     a6_huang_run1.m
%     a6_huang_spindle_detection0.m
%     a6_huang_init_bounds.m
%     a6_huang_init_data.m
%     a6_huang_train.m
%     a6_huang_test.m
%     rcada_rbsift_2.m
%     get_rUrLi_local.m
%     comp_rUrL.m
%     rcada_rb_init.m
% % % % % % % % % % % % % % % % % % % % % % % % % % % % % % % %
% % ..... beginning of a6_huang_run1.m .....
function [detect]=a6_huang_run1(C3,fs,p0)
% to find spindles using given parameter 'p0' for 1 data
% input: C3, (Nx1) refer to 'a0_preprocess_data.m'
%        fs: sample frequency
%        p0: parameter (refer to 'a6_huang_init_bounds.m')
% output: detect (Nx1) 1 for positive, 0 for negative detections
disp(['comp rU rL ...',' takes 10-20 minutes']);
[rU,rL]=comp_rUrL(C3,fs);
[a_imf,f_imf] = a6_huang_spindle_detection0(C3,rU,rL,fs);
detect = a6_huang_spindle_detection1_c(a_imf,f_imf,fs,p0);
end % % ....... end of a6_huang_run1.m .....

% % ..... beginning of a6_spindle_detection0.m .....
function [a_imf,f_imf] = ...
    a6_huang_spindle_detection0(C3,rU,rL,fs)
n=length(C3);
m=5;
a_imf=zeros(1,n);
f_imf=zeros(m,n);
[H,L,upper,lower]=...
    rcada_rbsift_2((1:length(C3))',C3,rU,rL,fs,10,0);
a_imf(1,:)=(upper-lower)';
for i=1:m
    [avgf] = avgfreq1(H,fs,i);
    for j=1:length(avgf)
        f_imf(i,j)=avgf(j);
    end
end

function [avgf] = avgfreq1(vimf,fs,w)
% compute frequency from w*(1/2) cycles from zero-crossings
len=length(vimf);
avgf=zeros(len,1);
```

```
hlf=zeros(len,1);
counter=1;
hlf(counter)=1;
if vimf(1)>=0
    flag=1;
else
    flag=0;
end
for i=2:len
    if flag>0
        if vimf(i)<0
            dt=min(1,vimf(i-1)/(vimf(i-1)-vimf(i)+0.0001));
            t1=i-1+dt; % zero-crossing
            counter=counter+1;
            hlf(counter)=t1;
            flag=0;
        end
    else
        if vimf(i)>=0
            dt=min(1,-vimf(i-1)/(vimf(i)-vimf(i-1)+0.0001));
            t1=i-1+dt; % zero-crossing
            counter=counter+1;
            hlf(counter)=t1;
            flag=1;
        end
    end
end
if t1<len
    counter=counter+1;
    hlf(counter)=len;
end
for i=1:counter-1
    i0=ceil(hlf(i));
    i1=floor(hlf(i+1));
    avgf(i0:i1)=0.5*fs/(hlf(i+1)-hlf(i));
end
if w>1
    for i=w:counter-w
        i0=ceil(hlf(i));
        i1=floor(hlf(i+1));
        avgf(i0:i1)=0.5*(2*w-1)*fs/(hlf(i+w)-hlf(i+1-w));
    end
end % % ....... end of a6_spindle_detection0.m .....

% % ..... beginning of a6_huang_init_bounds.m .....
function a6_huang_init_bounds
% init the searching boundaries of a6's operating parameters
global bounds;
% using 12 adjustable parameters (default value)
% p(1) fuzzy logic ramp distance below p(2)  (5uV)
% p(2) fuzzy logic lower amplitude boundary   (15uV)
% p(3) fuzzy logic amp plateau(?, upper bound=p(2)+p(3)uV)
% p(4) fuzzy logic ramp distance above p(3) (30 muV)
% p(5) fuzzy logic ramp distance below p(6) (0.5 Hz)
```

```
% p(6) fuzzy logic lower frequency boundary  (10 Hz)
% p(7) fuzzy logic freq plateau(6, upper bound=p(6)+p(7)Hz)
% p(8) fuzzy logic ramp distance above p(7) (0.5 Hz)
% p(9) the maximum instantaneous product threshold (0.5)
% p(10) pulse merging gap threshold (0.2s)
% p(11) shortest duration (0.3~0.5s)
% p(12) longest duration (3s)
% bounds of adjustable parameters
% (set the range of parameters as bounds=[min max;...])
bounds = [1  10;5 40;10 120; 1 50;0.1 4;8 13.5;1 8;0.1 4;...
    0.01 0.99;0.05 0.5;0.3 1;0.3 3];
end % % ....... end of a6_huang_init_bounds.m .....

% % ..... beginning of a6_huang_init_data.m .....
function a6_huang_init_data(Ovlp,database,trains,g_std,...
    p0,stime,seed2)
% Initialize a6's data struc for computational efficiency
% input arguments: see 'a0_train_test_MASS.m' for description
global ovlp;% JACCARD overlap coef for true detection grading
global aimfN;% store data for segments used in huang method
global fimfN;% store data for segments used in huang method
global DDN;% ground truth [heads,tails] for each segment
global d0N;% beginnings of segments
global lenM;% data signal length
global fsM;% sampling frequency for each segment
global segM;% number of segments in a dataset
global DNUM;% total number of datasets
ovlp=Ovlp;
% pre-process sleep dataset
DNUM=length(trains);% total number of training datasets
NUM=0;
for i=1:DNUM
    seg_no=0;
    % load training datasets
    [C3,stage,fs,DD]=get_datai_local(trains(i),g_std,database);
    [rU,rL]=get_rUrLi_local(trains(i),database,C3,fs);
    len=length(C3);
    % preprocessing datasets
    [a_imf,f_imf] = a6_huang_spindle_detection0(C3,rU,rL,fs);
    if nargin==4 % using the whole data as a segment for training
        % for small data such as the 'DREAMS' dataset
        NUM=NUM+1;
        seg_no=seg_no+1;
        aimfN{NUM}=a_imf;% intermediate data used in causa method
        fimfN{NUM}=f_imf;% intermediate data used in causa method
        DDN{NUM}=DD;% ground truth [heads,tails] for the i-th data
        d0N(NUM)=1;
        ttime=length(C3)/fs/60;T0=ttime;
    elseif nargin==7 % using p0 to crop segments for long C3 data
        % for long C3 data such as the 'MASS' database
        detection = ...
            a6_huang_spindle_detection1_c(a_imf,f_imf,fs,p0);
        % get [heads tails] of detections
        dd=detection2location(detection);
```

```
        [SS,T0]=compCroppedSegments(dd,DD,length(C3),...
            fs,stime,seed2);%crop
        ttime=sum(SS(:,2)-SS(:,1))/fs/60;
        for j=1:size(SS,1) %cropped segments from whole C3
            NUM=NUM+1;
            seg_no=seg_no+1;
            d0=SS(j,1);d1=SS(j,2); % head and tail of a segment
            aimfN{NUM}=a_imf(:,d0:d1);
            fimfN{NUM}=f_imf(:,d0:d1);
            d0N(NUM)=d0;
            ss=compSpindles(DD,d0,d1);% get ground truth
            % groundtruth local head-tail locations
            DDN{NUM}=ss-d0+1;
        end
    end
    lenM(i)=length(C3);% signal length
    segM(i)=seg_no;% number of segments in the i-th data
    fsM(i)=fs;% sampling frequency for the i-th data
    disp(['data',num2str(i),' ',num2str(seg_no),...
        ' segments',' ',num2str(ttime,3),' out of ',...
        num2str(T0,3),' minutes']);
end % end of for-i loop
end % % ....... end of a6_huang_init_data.m .....

% % ..... beginning of a6_huang_train.m .....
function [R]=a6_huang_train(p)
% called by spea2 moea during optimization
global ovlp;% JACCARD overlap coef for true detection grading
global aimfN;% store data used in causa method
global fimfN;% store data used in causa method
global DDN;% ground truth [heads,tails] for each segment
global fsM;% sampling frequency for each dataset
global segM;% number of segments in each dataset
global DNUM;% total number of datasets
R = [Inf, Inf];
FP=0;% false positives
FN=0;% false negatives
num=0;
for i=1:DNUM
    fs=fsM(i);
    for j=1:segM(i)
        num=num+1;
        detect = a6_huang_spindle_detection1_c(...
            aimfN{num},fimfN{num},fs,p);
        [fp,fn,~]=assessByEventJaccard_c(detect,DDN{num},ovlp);
        FN=FN+fn;
        FP=FP+fp;
    end
end
R(1)=FP;
R(2)=FN;
end % % ....... end of a6_huang_train.m .....

% % ..... beginning of a6_huang_test.m .....
```

```
function [P,R,F1]=a6_huang_test(database,tests,genes,ovlp,g_std)
% test the parameters in 'genes' for data listed in tests
% refer to 'a0_demo_train_and_test.m' for input arg' description
[PN]=compTotalTP(database,tests,g_std);%find total true positives
npar=size(genes,1);% number of parameter sets
FP=zeros(npar,1);% false positives by each parameter
FN=zeros(npar,1);% false negatives by each parameter
for i=1:length(tests)
    disp(tests(i));
    [C3,stage,fs,DD]=get_datai_local(tests(i),g_std,database);
    [rU,rL]=get_rUrLi_local(tests(i),database,C3,fs);
    [a_imf,f_imf] = a6_huang_spindle_detection0(C3,rU,rL,fs);
    for j=1:npar
        p0=genes(j,:);
        detect= a6_huang_spindle_detection1_c(a_imf,f_imf,fs,p0);
        [fp,fn,tp]=assessByEventJaccard_c(detect,DD,ovlp);
        FN(j)=FN(j)+fn;
        FP(j)=FP(j)+fp;
    end
end
TP=PN-FN;% detected true positives
P=TP./(TP+FP);% precision
R=TP./(TP+FN);% recall
F1=2*P.*R./(P+R);% F1-score
end % % ....... end of a6_huang_test.m .....

% % ..... beginning of rcada_rbsift_2.m .....
function [H,L,upper,lower]=rcada_rbsift_2(x,y,rU,rL,fs,ft,flag)
% rolling ball sifting algorithm
% input: y signal
%        fs signal sample frequency
%        ft threshold (cutoff) frequency
%        s re-scale factor y=s*y
%        flag>0 to draw results
% output: H high frequency component
%         L low frequency component
%         upper envelope
%         lower envelope
% author: Adam Huang, RCADA, National Central University
% email: adamhuan@gmail.com
% date: 2016/03/17

% Eq. (1), R-ball's radius for frequency sifting threshold: Ft
r=fs/ft/4;
% % step 0--get the radius info rU,rL by Delaunay triangulation

% % step 1--find alpha-shape envelope by rolling ball radius r
iU1=(rU>=r); % upper envelope pts touched by upper ball
iL1=(rL>=r); % lower envelope pts touched by lower ball
% first and last points are also marked as "touched"
iU1(1)=1;iL1(1)=1;iU1(end)=1;iL1(end)=1;
% % step 2--inflate and form init intermittent signal segments
iU2=iU1;iL2=iL1; % updated point touching info
% local max and min points
```

```
iMX=((y-[y(1);y(1:end-1)]>=0) & (y-[y(2:end);y(end)]> 0)) |...
    ((y-[y(1);y(1:end-1)]> 0) & (y-[y(2:end);y(end)]>=0));
iMN=((y-[y(1);y(1:end-1)]<=0) & (y-[y(2:end);y(end)]< 0)) |...
    ((y-[y(1);y(1:end-1)]< 0) & (y-[y(2:end);y(end)]<=0));
ii=(iU1>iL1); % pts touched by upper R-ball but not lower one
jj=ii & iMX; % plus condition: local maxima
[sg0,sg1]=findSegments(ii); %segments touched by upperball only
for i=1:length(sg0) %only keep local max touched by upperball
    iU2(sg0(i):sg1(i))=jj(sg0(i):sg1(i));
end
ii=(iL1>iU1); % pts touched by lower R-ball but not upper one
jj=ii & iMN; % plus condition: local minima
[sg0,sg1]=findSegments(ii);%segments touched by lowerball only
for i=1:length(sg0) % only keep local min touched by lowerball
    iL2(sg0(i):sg1(i))=jj(sg0(i):sg1(i));
end
iL2(1)=1;iL2(end)=1;iU2(1)=1;iU2(end)=1;
% % step 3 recover faint local extrema
iU3=iU2;iL3=iL2; % updated point touching info
iL3(iU2>0 & iMX)=0;... % Eq. (2) % recover touched maxima
iU3(iL2>0 & iMN)=0;... % Eq. (3) % recover touched minima
iL3(1)=1;iL3(end)=1;iU3(1)=1;iU3(end)=1;
% % step 4-- merge fragmental segments
iU4=iU3;iL4=iL3;  % updated point touching info
pflag=0;% peak flag
tflag=0;% trough flag
for i=2:length(y)-1
    if iU3(i)>0 && iL3(i)<1 % envelope local peak
        ipeak=i;
        pflag=1;
    elseif iU3(i)<1 && iL3(i)>0 % envelope local trough
        itrou=i;
        tflag=1;
    end
    % merge any peak-trough segment <= 2*r+2
    if pflag>0 && tflag>0
        if itrou>ipeak
            pflag=0;
            % allows some peak-trough error
            if x(itrou)-x(ipeak)<=2*r+0.5
                iU4(ipeak+1:itrou-1)=0;
                iL4(ipeak+1:itrou-1)=0;
            end
        else
            tflag=0;
            % allows some peak-trough error
            if x(ipeak)-x(itrou)<=2*r+0.5
                iU4(itrou+1:ipeak-1)=0;
                iL4(itrou+1:ipeak-1)=0;
            end
        end
    end
end
% % stap 5--remove single peaks and troughs
```

```
% exam every inflated segment and count its peaks and troughs
% remove stand-alone peaks and trough
iU5=iU4;iL5=iL4; % updated point touching info
fflag=0;
npeak=0;
ntrou=0;
for i=2:length(y)-1
    switch (fflag)
        case 0
            if iU4(i)<1 || iL4(i)<1 % inflated segment starts
                b0=i;
                fflag=1;
            end
        case 1
            if iU4(i)>0 && iL4(i)>0 % inflated segment end
                fflag=0;
                if npeak<1 || ntrou<1 %stand alone peak/trough
                    iU5(b0:i-1)=1;
                    iL5(b0:i-1)=1;
                end
                npeak=0; % reset peak counter
                ntrou=0; % reset trough counter
            end
            if iU4(i)>0 && iL4(i)<1 % peak
                npeak=npeak+1;
            end
            if iL4(i)>0 && iU4(i)<1 % trough
                ntrou=ntrou+1;
            end
    end
end
% % step 6--compute upper/lower envelopes
% %         and derive high/low-freq components
upper=envlp(x,y,find(iU5>0));% rolling ball upper envelope
lower=envlp(x,y,find(iL5>0));% rolling ball lower envelope
%low-freq component as the mean of upper&lower envelopes
L=(upper+lower)/2;
H=y-L;% high-freq component
% % draw results if flag is set
if flag>0
    mx=max(y(:));
    figure;
    hold on;%original data
    drawResults(x,y-2*mx,iU1,iL1);%initial alpha-shape envelope
    drawResults(x,y-4*mx,iU2,iL2);%inflate
    drawResults(x,y-6*mx,iU4,iL4);%recover faint signal and merge
    drawResults(x,y-8*mx,iU5,iL5);%remove stand alone peak/trough
    plot(x,y-10*mx,'k',x,H-10*mx,'r');%high-freq component
    plot(x,y-12*mx,'k',x,L-12*mx,'g');%low-freq component
    hold off;
end

function [y1]=envlp(x,y,iX)
% find the envelope with spline using pchip
```

```
x0=x(iX);
y0=y(iX);
y1=pchip(x0,y0,x);

function [sg0,sg1]=findSegments(ii)
ii=double(ii);
ii0=[0;ii(1:end-1)]-ii;
sg0=find(ii0<0);% strip starting
ii1=ii-[ii(2:end);0];
sg1=find(ii1>0);% strip ending

function drawResults(x,y,iU,iL)
upper=envlp(x,y,find(iU>0)); % piecewise linear upper envelope
lower=envlp(x,y,find(iL>0)); % piecewise linear lower envelope
plot(x,y,'k','LineWidth',0.5);
plot(x,upper,'k','LineWidth',0.75);
plot(x,lower,'k','LineWidth',0.75);
plot(x,(upper+lower)/2,'k--','LineWidth',0.75);
% % ....... end of rcada_rbsift_2.m .....

% % ..... beginning of get_rUrLi_local.m .....
function [rU,rL]=get_rUrLi_local(i,datadir,C3,fs)
tmp=exist(['..\',datadir,'\rUrL',num2str(i),'.mat'],'file');
if tmp>0
    load(['..\',datadir,'\rUrL',num2str(i),'.mat']);
else
    [rU,rL]=comp_rUrL(C3,fs);
end % % ....... end of get_rUrLi_local.m .....

% % ..... beginning of comp_rUrL.m .....
function [rU,rL]=comp_rUrL(C3,fs)
leny=length(C3);
rU=zeros(leny,1);
rL=zeros(leny,1);
ws=0.95;
s=ws*fs/50;
epoch=floor(leny/600/fs);%600 seconds/epoch
for j=1:epoch
    % jj0...30s...j0......600s......j1...30s...jj1
    % 1...........k0..........................k1
    j0=(j-1)*600*fs+1;
    j00=max(1,j0-30*fs);
    k0=j0-j00+1;
    j1=j*600*fs;
    if j<epoch
        j11=min(leny,j1+30*fs);
    else
        j11=leny;
    end
    k1=j11-j00+1;
    [~,~,rU0,rL0]=rcada_rb_init(C3(j00:j11),s);
    rU(j0:j11)=rU0(k0:k1);
    rL(j0:j11)=rL0(k0:k1);
end % % ....... end of comp_rUrL.m .....
```

```
% % ..... beginning of rcada_rb_init.m .....
function [x0,y0,rU,rL]=rcada_rb_init(y0,s)
% % arrange data as a column (m by 1) vector
[nrow,ncol]=size(y0);
if nrow<ncol
   y0=y0';
end
% % step 0--find the radius info rU,rL by Delaunay triangulation
% preprocessing data, find touching ball's radius info
% rU & rL (Eqs. 2&3) for upper envelope and lower envelope
x0=(1:length(y0))';
% find tangibility radius info by Delaunay triangulation
[x,y,ix]=resample1(x0,y0,s);% insert extra data pts if necessary
TR=delaunay(x,y);% Delaunay triangulation
% sort triangles (denoted as ABC) so x-coord: x(A)<x(B)<x(C)
TR=sort(TR,2);
AC=[x(TR(:,3))-x(TR(:,1)) y(TR(:,3))-y(TR(:,1))];% vectors AC
BC=[x(TR(:,3))-x(TR(:,2)) y(TR(:,3))-y(TR(:,2))];% vectors BC
AB=[x(TR(:,2))-x(TR(:,1)) y(TR(:,2))-y(TR(:,1))];% vectors AB
NAC=sqrt(sum(AC.*AC,2));% lengths of side AC
NBC=sqrt(sum(BC.*BC,2));% lengths of side BC
NAB=sqrt(sum(AB.*AB,2));% lengths of side AB
AREA=(AB(:,1).*AC(:,2)-AC(:,1).*AB(:,2))*0.5;%area by cross product
RADI=(NAC.*NBC.*NAB./abs(AREA))*0.25;% circumscribed radius formula
% find rU and rL Eqs. (a) and (b)
rU=zeros(length(x),1);
rL=zeros(length(x),1);
for i=1:size(TR,1)
    if AREA(i)>=0 % triangle is above the curve
        for j=1:3
            rU(TR(i,j))=max(rU(TR(i,j)),RADI(i));
        end
    else % triangle is below the curve
        for j=1:3
            rL(TR(i,j))=max(rL(TR(i,j)),RADI(i));
        end
    end
end
rU=rU(ix); % only return for the original data points
rL=rL(ix);

function [x1,y1,ix]=resample1(x,y,s)
% resample data so that distance between neighbor points < 1
len=length(x);
ix=(1:len)';% index to the original data position
y=y*s;% rescale
dx=x(2:len)-x(1:len-1);
dy=y(2:len)-y(1:len-1);
d=floor(((dx.^2+dy.^2).^0.5));% distance between points
% if neighbor points' distance d >= 1, insert d points
len1=len+sum(d);
x1=zeros(len1,1);
% arrange insertion
```

```
counter=0;
for i=1:len-1
    counter=counter+1;
    ix(i)=counter;
    x1(counter)=x(i);
    dx=(x(i+1)-x(i))/(d(i)+1);
    for j=1:d(i)
        counter=counter+1;
        x1(counter)=x(i)+j*dx;
    end
end
counter=counter+1;
ix(len)=counter;
x1(counter)=x(len);
y1=pchip(x,y,x1);% monotone piecewise cubic spline interpolation
% % ....... end of rcada_rb_init.m .....

% % % % % % % % % % % % % % % % % % % % % % % % % % % % % % % %
% 6.2 prepare c code for training detector d6
%     copy the following c file and save in the directory
%     a6_huang_spindle_detection1_c.c
%     build MEX function "mex a6_huang_spindle_detection1_c.c"
%     a mex file a6_huang_spindle_detection1_c.mexw64 is created
% % % % % % % % % % % % % % % % % % % % % % % % % % % % % % % %
//% % ..... beginning of a6_huang_spindle_detection1_c.c .....
#include "mex.h"
double fuzzi(double t,double p1,double p2, double p3, double p4);
/* rbsift_causa_spindle_detection1_c(a_imf,f_imf,fs,p);*/
void mexFunction(int nlhs,mxArray *plhs[],int nrhs,const mxArray
 *prhs[])
{
    int col,row,i,j,k,flag,j0,j1;
    double *ptr,*ptr1,*ptr2;
    double fs,p1,p2,p3,p4,p5,p6,p7,p8,p9,p10,p11,p12;
    double t,t1,t2;

    col=mxGetN(prhs[0]);
    row=mxGetM(prhs[1]);
    fs=*mxGetPr(prhs[2]);
    ptr=mxGetPr(prhs[3]);
    p1=*ptr;
    p2=*(ptr+1);
    p3=*(ptr+2);
    p4=*(ptr+3);
    p1=p2-p1;
    p3=p2+p3;
    p4=p3+p4;
    p5=*(ptr+4);
    p6=*(ptr+5);
    p7=*(ptr+6);
    p8=*(ptr+7);
    p5=p6-p5;
    p7=p6+p7;
    p8=p7+p8;
```

```
p9=*(ptr+8);
p10=*(ptr+9);
p11=*(ptr+10);
p12=*(ptr+11);
p10=p10*fs;
p11=p11*fs;
p12=p12*fs;
plhs[0]=mxCreateDoubleMatrix(col,1,mxREAL);
ptr =mxGetPr(plhs[0]);
ptr1=mxGetPr(prhs[0]);
ptr2=mxGetPr(prhs[1]);
for (j=0;j<col;j++) {
    t=0;
    t1=fuzzi(ptr1[j],p1,p2,p3,p4);
    if (t1>0) {
        k=row*j;
        for (i=0;i<row;i++) {//row == 5 the default setting
            t2=fuzzi(ptr2[k],p5,p6,p7,p8);
            t2*=t1;
            if (t2 > t) {
                t=t2;
            }
            k++;
        }
    }
    if (t>=p9) {
        ptr[j]=1;
    }
    else {
        ptr[j]=0;
    }
}
flag=0;//merge
for (j=0;j<col;j++) {
    switch (flag) {
        case 0:
            if (ptr[j]>0) {
                j0=j;
                flag=1;
            }
            break;
        case 1:
            if (ptr[j]>0) {
                j0=j;
            }
            else {
                flag=2;
            }
            break;
        case 2:
            if (ptr[j]>0) {
                j1=j;
                if (j1-j0 < p10) {
                    for (i=j0+1;i<j1;i++) {
```

```
                            ptr[i]=1;
                        }
                    }
                    j0=j;
                    flag=1;
                }
                break;
            default:
                break;
        }
    }
    flag=0;//duration check
    for (j=0;j<col;j++) {
        switch (flag) {
            case 0:
                if (ptr[j]>0) {
                    j0=j;
                    flag=1;
                }
                break;
            case 1:
                if (ptr[j]<1) {
                    j1=j;
                    flag=0;
                    if ((j1-j0 < p11) || (j1-j0 > p12)) {
                        for (i=j0;i<j1;i++) {
                            ptr[i]=0;
                        }
                    }
                }
                break;
            default:
                break;
        }
    }
}

double fuzzi(double t,double p1,double p2, double p3, double p4)
{
    double p;
    if ((t<=p1) || (t>=p4)) {
        p=0;
    }
    else {
        if (t<p2) {
            p=(t-p1)/(p2-p1);
        }
        else if (t<=p3) {
            p=1;
        }
        else {
            p=(p4-t)/(p4-p3);
        }
    }
```

```
    return p;
}//% % ....... end of a6_huang_spindle_detection1_c.c .....

% % % % % % % % % % % % % % % % % % % % % % % % % % % % % % % %
% 6.3 Preprocess rUrL1.mat to rUrL19.mat for MASS for a6 method
%     COPY the following M file in the directory
%     a6_pre_comp_rUrL.m
%     RUN a6_pre_comp_rUrL ONCE (only need to run this once)
%     >> a6_pre_comp_rUrL;
% % % % % % % % % % % % % % % % % % % % % % % % % % % % % % % %
% % ..... beginning of a6_pre_comp_rUrL.m .....
function a6_pre_comp_rUrL
datadir='MASS';
for i=1:19
    tmp=exist(['..\',datadir,'\data',num2str(i),'.mat'],'file');
    if tmp>0
        disp([num2str(i),' comp rU rL takes 10-20 minutes']);
        load(['..\',datadir,'\data',num2str(i),'.mat']);
        [rU,rL]=comp_rUrL(C3,fs);
        save(['..\',datadir,'\rUrL',num2str(i),'.mat'],'rU','rL');
    else
        txt=['..\',datadir,'\data',num2str(i),'.mat'];
        disp([txt,' FILE does not exist']);
    end
end % % ....... end of pre_comp_rUrL.m .....

% % % % % % % % % % % % % % % % % % % % % % % % % % % % % % % %
% 6.4 train/test detector d6 with the following command
%     >> a0_run_example(6,'1',60,1);or a0_run_example(6,'1');
%     >> a0_run_example(6,'2');
%     run d6 on any C3 data with a chosen p0
%     >> [detect]=a6_huang_run1(C3,fs,p0);
% p0=[9.5836 25.4728 57.2041 7.2513 1.7971 13.3680 1.0409 0.1004
%     0.3574 0.0501 0.4312 2.9909] s1-gstd
% p0=[9.8418 21.3246 59.971 12.735 0.3092 13.1939 1.0204 0.2523
%     0.4068 0.1027 0.3946 2.9998] s2-gstd
% % % % % % % % % % % % % % % % % % % % % % % % % % % % % % % %


% % % % % % % % % % % % % % % % % % % % % % % % % % % % % % % %
% 7.1 prepare code for training detector d7
%     copy the following 5 M files and save in the directory
%     a7_huang_moelle_run1.m
%     a7_huang_moelle_init_bounds.m
%     a7_huang_moelle_init_data.m
%     a7_huang_moelle_train.m
%     a7_huang_moelle_test.m
% % % % % % % % % % % % % % % % % % % % % % % % % % % % % % % %
% % ..... beginning of a7_huang_moelle_run1.m .....
function detect=a7_huang_moelle_run1(C3,stage,fs,p0)
% to find spindles using given parameter 'p0' for 1 data
% input: C3, (Nx1) refer to 'a0_preprocess_data.m'
%        fs: sample frequency
%        p0: parameter (refer to 'a7_causa_init_bounds.m')
```

```
% output: detect (Nx1) 1 for positive, 0 for negative detections
[rU,rL]=comp_rUrL(C3,fs);
[a_imf,f_imf] = a6_huang_spindle_detection0(C3,rU,rL,fs);
detect = a6_huang_spindle_detection1_c(a_imf,f_imf,fs,p0(1:12));
[C3_band,std_C3band] = a2_moelle_spindle_detection0(C3,stage,fs);
bandsq=C3_band.^2;
detect = a2_moelle_spindle_detection1_c(bandsq.*detect,...
            std_C3band,fs,p0(13:17));
end % % ....... end of a7_huang_moelle_run1.m .....

% % ..... beginning of a7_huang_moelle_init_bounds.m .....
function a7_huang_moelle_init_bounds
% initialize the boundaries of a7's operating parameters
global bounds;
% using 17 adjustable parameters
% (p(1:12), huang_alg; p(13:17), moelle_alg)
% p(1) fuzzy logic ramp distance below p(2)  (5 muV)
% p(2) fuzzy logic lower amplitude boundary   (15 muV)
% p(3) fuzzy logic amp plateau width(?,upper bound=p(2)+p(3)uV)
% p(4) fuzzy logic ramp distance above upper bound (30 uV)
% p(5) fuzzy logic ramp distance below p(6) (0.5 Hz)
% p(6) fuzzy logic lower frequency boundary  (10 Hz)
% p(7) fuzzy logic freq plateau width(6,upper bound=p(6)+p(7)Hz)
% p(8) fuzzy logic ramp distance above upper boundary (0.5 Hz)
% p(9) the maximum instantaneous product threshold (0.5)
% p(10) time merging gap threshold (0.2s)
% p(11) shortest duration (0.3~0.5s)
% p(12) longest duration (3s)
% p(13) time resolution (0.05s)
% p(14) rms window (0.1s)
% p(15) spindle upper boundary threshold ratio (1.5)
% p(16) shortest duration (0.3~0.5s)
% p(17) longest duration (3s)
% bounds of adjustable parameters
% (set the range of parameters as bounds=[min max;...])
bounds = [1 10;5 40;10 120;1 50;0.1 4;8 13.5;1 8;0.1 4;...
    0.01 0.99;0.05 0.5;0.3 1;0.3 3;... %p(1:12)
    0.05 0.5;0.05 0.5;0.1 10;0.3 1;0.3 3]; %p(13:17)
end % % ....... end of a7_huang_moelle_init_bounds.m .....

% % ..... beginning of a7_huang_moelle_init_data.m .....
function a7_huang_moelle_init_data(Ovlp,database,trains,...
        g_std,p0,stime,seed2)
% Initialization of a7's data struc for computational efficiency
% input arguments: see 'a0_demo_moea.m' for description
global ovlp;% JACCARD overlap coef for true detection grading
global aimfN;% store data for segments used in huang method
global fimfN;% store data for segments used in huang method
global bandsqN;% store data used in moelle method
global DDN;% ground truth [heads,tails] for each segment
global d0N;% beginnings of segments
global stdM;% store data used in moelle method
global lenM;% data signal length
global fsM;% sampling frequency for each data
```

```
global segM;% number of segments in a dataset
global DNUM;% total number of datasets
ovlp=Ovlp;
% pre-process sleep dataset
DNUM=length(trains);% total number of training datasets
NUM=0;
for i=1:DNUM
    seg_no=0;
    % load training datasets
    [C3,stage,fs,DD]=get_datai_local(trains(i),g_std,database);
    [rU,rL]=get_rUrLi_local(trains(i),database,C3,fs);
    % preprocessing datasets
    [a_imf,f_imf] = a6_huang_spindle_detection0(C3,rU,rL,fs);
    [C3_band,std_C3band] = ...
        a2_moelle_spindle_detection0(C3,stage,fs);
    bandsq=C3_band.^2;
    if nargin==4 % use the whole data as a segment for training
        % for small data such as the 'DREAMS' dataset
        NUM=NUM+1;
        seg_no=seg_no+1;
        aimfN{NUM}=a_imf;% intermediate data used in huang_alg
        fimfN{NUM}=f_imf;% intermediate data used in huang_alg
        bandsqN{NUM}=bandsq;% intermed data used in moelle_alg
        DDN{NUM}=DD;
        d0N(NUM)=1;
        ttime=length(C3)/fs/60;T0=ttime;
    elseif nargin==7 % use p0 to crop segments for long C3 data
        % for long C3 data such as the 'MASS' database
        % apply a6_huang algorithm first
        detect0 = a6_huang_spindle_detection1_c(a_imf,...
            f_imf,fs,p0(1:12));
        % then apply a2_moelle algorithm
        detect = a2_moelle_spindle_detection1_c(...
            bandsq.*detect0,std_C3band,fs,p0(13:17));
        % get [heads tails] of detections
        dd=detection2location(detect);
        [SS,T0]=compCroppedSegments(dd,DD,length(C3),...
            fs,stime,seed2);%crop
        ttime=sum(SS(:,2)-SS(:,1))/fs/60;
        for j=1:size(SS,1) %cropped segments from long C3 signal
            NUM=NUM+1;
            seg_no=seg_no+1;
            d0=SS(j,1);d1=SS(j,2); % head and tail of a segment
            aimfN{NUM}=a_imf(:,d0:d1);
            fimfN{NUM}=f_imf(:,d0:d1);
            bandsqN{NUM}=bandsq(d0:d1);
            d0N(NUM)=d0;
            ss=compSpindles(DD,d0,d1);% get ground truth
            % ground truth local head-tail locations
            DDN{NUM}=ss-d0+1;
        end
    end
    lenM(i)=length(C3);% signal length
    segM(i)=seg_no;% number of segments in each data
```

```
        stdM(i)=std_C3band; % intermediate data used in a2 alg
        fsM(i)=fs;% sampling frequency for the i-th data
        disp(['data',num2str(i),' ',num2str(seg_no),...
            ' segments',' ',num2str(ttime,3),' out of ',...
            num2str(T0,3),' minutes']);
    end % end of for-i loop
    end % % ....... end of a7_huang_moelle_init_data.m .....

    % % ..... beginning of a7_huang_moelle_train.m .....
    function [R]=a7_huang_moelle_train(p)
    % called by spea2 moea during optimization
    global ovlp;
    global aimfN;% store data for segments used in causa method
    global fimfN;% store data for segments used in causa method
    global bandsqN;% store data used in moelle method
    global stdM;% store data used in moelle method
    global DDN;% ground truth [heads,tails] for spindles
    global fsM;% sampling frequency for each segment
    global segM;% number of segments in a dataset
    global DNUM;% total number of datasets
    R = [Inf, Inf];
    FP=0;% false positives
    FN=0;% false negatives
    num=0;
    for i=1:DNUM
        fs=fsM(i);
        for j=1:segM(i)
            num=num+1;
            detect = a6_huang_spindle_detection1_c(aimfN{num},...
                fimfN{num},fs,p(1:12));
            detect = a2_moelle_spindle_detection1_c(...
                bandsqN{num}.*detect,stdM(i),fs,p(13:17));
            [fp,fn,~]=assessByEventJaccard_c(detect,DDN{num},ovlp);
            FN=FN+fn;
            FP=FP+fp;
        end
    end
    R(1)=FP;
    R(2)=FN;
    end % % ....... end of a7_huang_moelle_train.m .....

    % % ..... beginning of a7_huang_moelle_test.m .....
    function [P,R,F1]=...
            a7_huang_moelle_test(database,tests,genes,ovlp,g_std)
    % test the parameters in 'genes' for data listed in tests
    % refer to 'a0_train_test_MASS.m' for input arg description
    [PN]=compTotalTP(database,tests,g_std);%find total true positives
    npar=size(genes,1);% number of parameter sets
    FP=zeros(npar,1);% false positives by each parameter
    FN=zeros(npar,1);% false negatives by each parameter
    for i=1:length(tests)
        disp(tests(i));
        [C3,stage,fs,DD]=get_datai_local(tests(i),g_std,database);
        [rU,rL]=get_rUrLi_local(tests(i),database,C3,fs);
```

```
    % preprocessing datasets
    [a_imf,f_imf] = a6_huang_spindle_detection0(C3,rU,rL,fs);
    [C3_band,std_C3band] = ...
        a2_moelle_spindle_detection0(C3,stage,fs);
    bandsq=C3_band.^2;
    for j=1:npar
        p0=genes(j,:);
        % apply a6_huang_alg first
        detect0 = a6_huang_spindle_detection1_c(a_imf,...
            f_imf,fs,p0(1:12));
        % then apply a2_moelle_alg
        detect=a2_moelle_spindle_detection1_c(...
            bandsq.*detect0,std_C3band,fs,p0(13:17));
        [fp,fn,tp]=assessByEventJaccard_c(detect,DD,ovlp);
        FN(j)=FN(j)+fn;
        FP(j)=FP(j)+fp;
    end
end
TP=PN-FN;% detected true positives
P=TP./(TP+FP);% precision
R=TP./(TP+FN);% recall
F1=2*P.*R./(P+R);% F1-score
end % % ....... end of a7_huang_moelle_test.m .....

% % % % % % % % % % % % % % % % % % % % % % % % % % % % % % % %
% 7.2 prepare c code for training detector d7
%     RUN steps 2.1-2.2,6.1-6.3 before proceeding the following
%
% % % % % % % % % % % % % % % % % % % % % % % % % % % % % % % %

% % % % % % % % % % % % % % % % % % % % % % % % % % % % % % % %
% 7.3 train/test detector d7 with the following command
%     >> a0_run_example(7,'1',60,1);
%     >> a0_run_example(7,'2');
%     run d7 on any C3 data with a chosen p0
%     >> [detect]=a7_huang_moelle_run1(C3,stage,fs,p0);
% p0=[5.6312 18.2081 117.0672 18.6245 3.9949 13.4502 1.4203 1.214
%     0.6193 0.0503 0.4438 2.9987
%     0.0505 0.4896 1.4879 0.5431 2.9589] s1-gstd
% p0=[9.6855 16.4218 100.1513 5.0105 0.1529 12.3280 5.5736 3.7623
%     0.3793 0.0508 0.3439 2.9996
%     0.2602 0.4978 0.9646 0.4569 2.9869] s2-gstd
% % % % % % % % % % % % % % % % % % % % % % % % % % % % % % % %

% % % % % % % % % % % % % % % % % % % % % % % % % % % % % % % %
% 8.1 prepare code for training detector d8
%     copy the following 6 M files and save in the directory
%     a8_moelle_huang_run1.m
%     a8_moelle_huang_init_bounds.m
%     a8_moelle_huang_init_data.m
%     a8_moelle_huang_train.m
%     a8_moelle_huang_test.m
%     comp_rUrL_dd.m
% % % % % % % % % % % % % % % % % % % % % % % % % % % % % % % %
```

```
% % ..... beginning of a8_moelle_huang_run1.m .....
function detect=a8_moelle_huang_run1(C3,stage,fs,p0)
% to find spindles using given parameter 'p0' for 1 data
% input: C3, (Nx1) refer to 'a0_preprocess_data.m'
%        fs: sample frequency
%        p0: parameter (refer to 'a8_causa_init_bounds.m')
% output: detect (Nx1) 1 for positive, 0 for negative detections
[C3_band,std_C3band] = a2_moelle_spindle_detection0(C3,stage,fs);
bandsq=C3_band.^2;
detect0 = a2_moelle_spindle_detection1_c(bandsq,...
    std_C3band,fs,p0(1:5));
dd=detection2location(detect0);
[rU,rL]=comp_rUrL_dd(C3,fs,dd);
[a_imf,f_imf] = a6_huang_spindle_detection0(C3,rU,rL,fs);
detect=a8_moelle_huang_spindle_detection1_c(a_imf,...
    f_imf,detect0,fs,p0(6:17));
end % % ....... end of a8_moelle_huang_run1.m .....

% % ..... beginning of a8_moelle_huang_init_bounds.m .....
function a8_moelle_huang_init_bounds
% initialize the searching boundaries of a8's operating parameters
global bounds;
% using 17 adjustable parameters
% (p(1:5), moelle_alg; p(6:17), huang_alg)
% p(1) time resolution (0.05s)
% p(2) rms window (0.1s)
% p(3) spindle upper boundary threshold ratio (1.5)
% p(4) shortest duration (0.3~0.5s)
% p(5) longest duration (3s)
% p(6) fuzzy logic ramp distance below p(7)  (5 muV)
% p(7) fuzzy logic lower amplitude boundary   (15 muV)
% p(8) fuzzy logic amp plateau width(upper bound=p(7)+p(8)uV)
% p(9) fuzzy logic ramp distance above upper boundary (30 uV)
% p(10) fuzzy logic ramp distance below p(11) (0.5 Hz)
% p(11) fuzzy logic lower frequency boundary  (10 Hz)
% p(12) fuzzy logic freq width(upper bound=p(11)+p(12)Hz)
% p(13) fuzzy logic ramp distance above upper bound (0.5Hz)
% p(14) the maximum instantaneous product threshold (0.5)
% p(15) time merging gap threshold (0.2s)
% p(16) shortest duration (0.3~0.5s)
% p(17) longest duration (3s)
% bounds of adjustable parameters
% (set the range of parameters as bounds=[min max;...])
bounds = [0.05 0.5;0.05 0.5;0.1 10;0.3 1;0.3 3;... %p (1:5)
    1  10;5 40; 10 120; 1 50;0.1  4;8 13.5;1 8;0.1 4; ...
    0.01 0.99;0.05 0.5;0.3 1;0.3 3;];
end % % ....... end of a8_moelle_huang_init_bounds.m .....

% % ..... beginning of a8_moelle_huang_init_data.m .....
function a8_moelle_huang_init_data(Ovlp,database,trains,...
        g_std,p0,stime,seed2)
% Initialize a8's data struc for computational efficiency
% input arguments: see 'a0_train_test_MASS.m' for description
global ovlp;% JACCARD overlap coef for true detection grading
```

```
global aimfN;% store data for segments used in huang method
global fimfN;% store data for segments used in huang method
global bandsqN;% store data used in moelle method
global DDN;% ground truth [heads,tails] for each segment
global d0N;% beginnings of segments
global stdM;% store data used in moelle method
global lenM;% data signal length
global fsM;% sampling frequency for each data
global segM;% number of segments in a dataset
global DNUM;% total number of datasets
ovlp=Ovlp;
% pre-process sleep dataset
DNUM=length(trains);% total number of training datasets
NUM=0;
for i=1:DNUM
    seg_no=0;
    % load training datasets
    [C3,stage,fs,DD]=get_datai_local(trains(i),g_std,database);
    [rU,rL]=get_rUrLi_local(trains(i),database,C3,fs);
    % preprocessing datasets
    [a_imf,f_imf] = a6_huang_spindle_detection0(C3,rU,rL,fs);
    [C3_band,std_C3band] = ...
        a2_moelle_spindle_detection0(C3,stage,fs);
    bandsq=C3_band.^2;
    if nargin==4 % use the whole data as a segment for training
        % for small data such as the 'DREAMS' dataset
        NUM=NUM+1;
        seg_no=seg_no+1;
        aimfN{NUM}=a_imf;% intermediate data used in huang_alg
        fimfN{NUM}=f_imf;% intermediate data used in huang_alg
        bandsqN{NUM}=bandsq;% intermed data used in moelle_alg
        DDN{NUM}=DD;
        d0N(NUM)=1;
        ttime=length(C3)/fs/60;T0=ttime;
    elseif nargin==7 % use p0 to crop segments for long C3 data
        % for long C3 data such as the 'MASS' database
        detect0 = a2_moelle_spindle_detection1_c(bandsq,...
            std_C3band,fs,p0(1:5)); % apply a2_moelle first
        detect=a8_moelle_huang_spindle_detection1_c(a_imf,...
            f_imf,detect0,fs,p0(6:17)); % then apply a6_huang
        % get [heads tails] of detections
        dd=detection2location(detect);
        [SS,T0]=compCroppedSegments(dd,DD,length(C3),fs,...
            stime,seed2);%crop
        ttime=sum(SS(:,2)-SS(:,1))/fs/60;
        for j=1:size(SS,1) %cropped segments from long C3 signal
            NUM=NUM+1;
            seg_no=seg_no+1;
            d0=SS(j,1);d1=SS(j,2); % head and tail of a segment
            aimfN{NUM}=a_imf(:,d0:d1);
            fimfN{NUM}=f_imf(:,d0:d1);
            bandsqN{NUM}=bandsq(d0:d1);
            d0N(NUM)=d0;
            ss=compSpindles(DD,d0,d1);
```

```
            DDN{NUM}=ss-d0+1;
        end
    end
    lenM(i)=length(C3);% signal length
    segM(i)=seg_no;% number of segments in each data
    stdM(i)=std_C3band;  % intermed data used in Moelle method
    fsM(i)=fs;% sampling frequency for the i-th data
    disp(['data',num2str(i),' ',num2str(seg_no),...
        ' segments',' ',num2str(ttime,3),' out of ',...
        num2str(T0,3),' minutes']);
end % end of for-i loop
end % % ....... end of a8_moelle_huang_init_data.m .....

% % ..... beginning of a8_moelle_huang_train.m .....
function [R]=a8_moelle_huang_train(p)
% called by spea2 moea during optimization
global ovlp;
global aimfN;% store data for segments used in causa method
global fimfN;% store data for segments used in causa method
global bandsqN;% store data used in moelle method
global stdM;% store data used in moelle method
global DDN;% ground truth [heads,tails] for spindles
global fsM;% sampling frequency for each segment
global segM;% number of segments in a dataset
global DNUM;% total number of datasets
R = [Inf, Inf];
FP=0;% false positives
FN=0;% false negatives
num=0;
for i=1:DNUM
    fs=fsM(i);
    for j=1:segM(i)
        num=num+1;
        detect = a2_moelle_spindle_detection1_c(...
            bandsqN{num},stdM(i),fs,p(1:5));
        detect = a8_moelle_huang_spindle_detection1_c(...
            aimfN{num},fimfN{num},detect,fs,p(6:17));
        [fp,fn,~]=assessByEventJaccard_c(detect,DDN{num},ovlp);
        FN=FN+fn;
        FP=FP+fp;
    end
end
R(1)=FP;
R(2)=FN;
end % % ....... end of a8_moelle_huang_train.m .....

% % ..... beginning of a8_moelle_huang_test.m .....
function [P,R,F1]=...
    a8_moelle_huang_test(database,tests,genes,ovlp,g_std)
% test the parameters in 'genes' for data listed in tests
% refer to 'a0_train_test_MASS.m' for input arg description
[PN]=compTotalTP(database,tests,g_std);%find total true positives
npar=size(genes,1);% number of parameter sets
FP=zeros(npar,1);% false positives by each parameter
```

```
FN=zeros(npar,1);% false negatives by each parameter
for i=1:length(tests)
    disp(tests(i));
    [C3,stage,fs,DD]=get_datai_local(tests(i),g_std,database);
    [rU,rL]=get_rUrLi_local(tests(i),database,C3,fs);
    % preprocessing datasets
    [a_imf,f_imf] = a6_huang_spindle_detection0(C3,rU,rL,fs);
    [C3_band,std_C3band] = ...
        a2_moelle_spindle_detection0(C3,stage,fs);
    bandsq=C3_band.^2;
    for j=1:npar
        p0=genes(j,:);
        detect0 = a2_moelle_spindle_detection1_c(bandsq,...
            std_C3band,fs,p0(1:5)); % apply a2_moelle first
        detect=a8_moelle_huang_spindle_detection1_c(...
            a_imf,f_imf,detect0,fs,p0(6:17)); % then apply a6
        [fp,fn,tp]=assessByEventJaccard_c(detect,DD,ovlp);
        FN(j)=FN(j)+fn;
        FP(j)=FP(j)+fp;
    end
end
TP=PN-FN;% detected true positives
P=TP./(TP+FP);% precision
R=TP./(TP+FN);% recall
F1=2*P.*R./(P+R);% F1-score
end % % ....... end of a8_moelle_huang_test.m .....

% % ..... beginning of comp_rUrL_dd.m .....
function [rU,rL]=comp_rUrL_dd(C3,fs,dd)
leny=length(C3);
rU=zeros(leny,1);
rL=zeros(leny,1);
ws=0.95;
s=ws*fs/50;
seg=size(dd,1);% 5 seconds/segment
for j=1:seg
    % j00....1s....j0......5s......j1....1s....j11
    % 1............k0..............k1
    t1=dd(j,1);t2=dd(j,2);
    j0=max(1,floor((t2+t1)/2-2.5*fs));% begin of cropped segments
    j00=max(1,j0-fs);
    j1=min(leny,floor(j0+5*fs));      % end of cropped segments
    k0=j0-j00+1;
    j11=min(leny,j1+fs);
    k1=j1-j00+1;
    [~,~,rU0,rL0]=rcada_rb_init(C3(j00:j11),s);
    rU(j0:j1)=rU0(k0:k1);
    rL(j0:j1)=rL0(k0:k1);
end % % ....... end of comp_rUrL_dd.m .....

% % % % % % % % % % % % % % % % % % % % % % % % % % % % % % % %
% 8.2 prepare c code for training detector d8
%     RUN steps 2.1-2.2,6.1-6.3 before proceeding the following
%     copy the following c file and save in the directory
```

```
%     a8_moelle_huang_spindle_detection1_c.c
%     build MEX function
%     >> mex a8_moelle_huang_spindle_detection1_c.c
%     a8_moelle_huang_spindle_detection1_c.mexw64 is created
% % % % % % % % % % % % % % % % % % % % % % % % % % % % % % % %
//% % .. beginning of a8_moelle_huang_spindle_detection1_c.c ..
#include "mex.h"
double fuzzi(double t,double p1,double p2, double p3, double p4);
/* rbsift_causa_spindle_detection1_c(a_imf,f_imf,dd,fs,p); */
void mexFunction(int nlhs,mxArray *plhs[],int nrhs,const mxArray
 *prhs[])
{
    int col,row,i,j,k,flag,j0,j1;
    double *ptr,*ptr1,*ptr2,*dd;
    double fs,p1,p2,p3,p4,p5,p6,p7,p8,p9,p10,p11,p12;
    double t,t1,t2;

    col=mxGetN(prhs[0]);
    row=mxGetM(prhs[1]);
    dd=mxGetPr(prhs[2]);
    fs=*mxGetPr(prhs[3]);
    ptr=mxGetPr(prhs[4]);
    p1=*ptr;
    p2=*(ptr+1);
    p3=*(ptr+2);
    p4=*(ptr+3);
    p1=p2-p1;
    p3=p2+p3;
    p4=p3+p4;
    p5=*(ptr+4);
    p6=*(ptr+5);
    p7=*(ptr+6);
    p8=*(ptr+7);
    p5=p6-p5;
    p7=p6+p7;
    p8=p7+p8;
    p9=*(ptr+8);
    p10=*(ptr+9);
    p11=*(ptr+10);
    p12=*(ptr+11);
    p10=p10*fs;
    p11=p11*fs;
    p12=p12*fs;
    plhs[0]=mxCreateDoubleMatrix(col,1,mxREAL);
    ptr =mxGetPr(plhs[0]);
    ptr1=mxGetPr(prhs[0]);
    ptr2=mxGetPr(prhs[1]);
    for (j=0;j<col;j++) {
        t=0;
        t1=fuzzi(ptr1[j],p1,p2,p3,p4);
        if (t1>0) {
            k=row*j;
            for (i=0;i<row;i++) {//row == 5 the default setting
                t2=fuzzi(ptr2[k],p5,p6,p7,p8);
```

```
                t2*=t1;
                if (t2 > t) {
                    t=t2;
                }
                k++;
            }
        }
        t*=dd[j];
        if (t>=p9) {
            ptr[j]=1;
        }
        else {
            ptr[j]=0;
        }
    }
    flag=0;//merge
    for (j=0;j<col;j++) {
        switch (flag) {
            case 0:
                if (ptr[j]>0) {
                    j0=j;
                    flag=1;
                }
                break;
            case 1:
                if (ptr[j]>0) {
                    j0=j;
                }
                else {
                    flag=2;
                }
                break;
            case 2:
                if (ptr[j]>0) {
                    j1=j;
                    if (j1-j0 < p10) {
                        for (i=j0+1;i<j1;i++) {
                            ptr[i]=1;
                        }
                    }
                    j0=j;
                    flag=1;
                }
                break;
            default:
                break;
        }
    }
    flag=0;//duration check
    for (j=0;j<col;j++) {
        switch (flag) {
            case 0:
                if (ptr[j]>0) {
                    j0=j;
```

```
                        flag=1;
                    }
                    break;
                case 1:
                    if (ptr[j]<1) {
                        j1=j;
                        flag=0;
                        if ((j1-j0 < p11) || (j1-j0 > p12)) {
                            for (i=j0;i<j1;i++) {
                                ptr[i]=0;
                            }
                        }
                    }
                    break;
                default:
                    break;
            }
        }
    }

    double fuzzi(double t,double p1,double p2, double p3, double p4)
    {
        double p;
        if ((t<=p1) || (t>=p4)) {
            p=0;
        }
        else {
            if (t<p2) {
                p=(t-p1)/(p2-p1);
            }
            else if (t<=p3) {
                p=1;
            }
            else {
                p=(p4-t)/(p4-p3);
            }
        }
        return p;
    }//% % ..... end of a8_moelle_huang_spindle_detection1_c.c ....

    % % % % % % % % % % % % % % % % % % % % % % % % % % % % % % % %
    % 8.3 train/test detector d8 with the following command
    %     >> a0_run_example(8,'1',60,1);or a0_run_example(8,'1');
    %     >> a0_run_example(8,'2');
    %     run d8 on any C3 data with a chosen p0
    %     >> [detect]=a8_moelle_huang_run1(C3,stage,fs,p0);
    % p0=[0.0501 0.4849 1.7724 0.3025 2.9903
    %    8.861 17.9827 95.4421 43.9608 1.0969 12.4204 4.9864 1.2069
    %     0.8015 0.05 0.4456 2.8276] s1-gstd
    % p0=[0.0529 0.4669 1.3288 0.415 2.5127
    %  4.4462 13.2504 112.8461 28.1524 1.1942 12.9668 5.2226 2.3198
    %     0.1145 0.0511 0.496 2.8055] s2-gstd
    % % % % % % % % % % % % % % % % % % % % % % % % % % % % % % % %
```

```
% % % % % % % % % % % % % % % % % % % % % % % % % % % % % % % %
% 9.1 prepare code for training detector d9
%     copy the following 4 M files and save in the directory
%     a9_tsanas_huang_init_bounds.m
%     a9_tsanas_huang_init_data.m
%     a9_tsanas_huang_train.m
%     a9_tsanas_huang_test.m
% % % % % % % % % % % % % % % % % % % % % % % % % % % % % % % %
% % ..... beginning of a9_tsanas_huang_init_bounds.m .....
function a9_tsanas_huang_init_bounds
% initialize searching boundaries of a9's operating parameters
global bounds;
% using 27 adjustable parameters (default value)
% p(1) spindle lower frequency boundary (11 Hz)
% p(2) spindle upper frequency boundary (16 Hz)
% p(3) smoothing window (0.11 s)
% p(4) threshold (0.3)
% p(5) (0.03 s) min duration
% p(6) (0.1 s) merge gap
% p(7) strong threshold (0.7)
% p(8) (0.1 s) duration
% p(9) (0.3 s) duration
% p(10) intermediate threshold (0.6)
% p(11) (0.1 s) duration
% p(12) (0.2 s) duration
% p(13) (0.3 s)
% p(14) shortest duration (0.4 s)
% p(15) longest mergeable duration (1.5 s)
% p(16) fuzzy logic ramp distance below p(17)  (5 muV)
% p(17) fuzzy logic lower amplitude boundary   (15 muV)
% p(18) fuzzy logic amp plateau(?,upper bound=p(17)+p(18) uV)
% p(19) fuzzy logic ramp distance above p(18) (30 muV)
% p(20) fuzzy logic ramp distance below p(21) (0.5 Hz)
% p(21) fuzzy logic lower frequency boundary  (10 Hz)
% p(22) fuzzy logic freq plateau(6,upper bound=p(21)+p(22)Hz)
% p(23) fuzzy logic ramp distance above p(22) (0.5 Hz)
% p(24) the maximum instantaneous product threshold (0.5)
% p(25) pulse merging gap threshold (0.2s)
% p(26) shortest duration (0.3~0.5s)
% p(27) longest duration (3s)
% bounds of adjustable parameters
% (set the range of parameters as bounds=[min max;...])

% bounds of adjustable parameters
% (set the range of parameters as bounds=[min max;...])
% p=     [11   16    0.11      0.3     0.03     0.1
%   0.7     0.1       0.3     0.6      0.1       0.2
%   0.3     0.4     1.5];
bounds = [8 12;14 18;0.05 0.25;0.1 0.8;0.01 0.1;0.05 0.25;...
    0.3 0.9;0.05 0.25;0.1 0.5;0.3 0.80;0.05 0.25;0.05 0.5;...
    0.1 0.5;0.3 1.0;0.3 3.0;...
    1  10;5 40;10 120; 1 50;0.1 4;8 13.5;1 8;0.1 4;...
    0.01 0.99;0.05 0.5;0.3 1;0.3 3];
```

```
end % % ....... end of a9_tsanas_huang_init_bounds.m .....

% % ..... beginning of a9_tsanas_huang_init_data.m .....
function a9_tsanas_huang_init_data(Ovlp,database,trains,g_std,...
    p0,stime,seed2)
% Initialize a8's data struc for computational efficiency
% input arguments: see 'a0_demo_moea.m' for description
global ovlp;% JACCARD overlap coef for true detection grading
global aimfN;% store data for segments used in huang method
global fimfN;% store data for segments used in huang method
global coefN;% store data used in tsanas method
global freqM;% store data used in tsanas method
global DDN;% ground truth [heads,tails] for each segment
global lenM;% data signal length
global fsM;% sampling frequency for each data
global segM;% number of segments in a dataset
global DNUM;% total number of datasets
ovlp=Ovlp;
% pre-process sleep dataset
DNUM=length(trains);% total number of training datasets
NUM=0;
for i=1:DNUM
    seg_no=0;
    % load training datasets
    [C3,stage,fs,DD]=get_datai_local(trains(i),g_std,database);
    [rU,rL]=get_rUrLi_local(trains(i),database,C3,fs);
    % preprocessing datasets
    [a_imf,f_imf] = a6_huang_spindle_detection0(C3,rU,rL,fs);
    K=10;% top K cwt coef, signal resampled to 100 Hz
    [CoefsTop10,pseudo_freqCWT,new_fs]=...
        a4_tsanas_spindle_detection0(C3,fs,K);
    DD=floor((DD-1)*new_fs/fs)+1;
    len=size(CoefsTop10,2);
    IX=(1:len)';
    ix=floor((IX-1)*fs/new_fs)+1;
    a_imf=a_imf(:,ix);
    f_imf=f_imf(:,ix);
    if nargin==4 % use the whole data as a segment for training
        % for small data such as the 'DREAMS' dataset
        NUM=NUM+1;
        seg_no=seg_no+1;
        aimfN{NUM}=a_imf;% intermediate data used in a6
        fimfN{NUM}=f_imf;% intermediate data used in a6
        coefN{NUM}=CoefsTop10';% intermed data used in a4
        DDN{NUM}=DD;
        ttime=length(C3)/fs/60;T0=ttime;
    elseif nargin==7 % use p0 to crop segments for long C3 data
        % for long C3 data such as the 'MASS' database
        % apply a4_tsanas first
        p=p0;
        [mini,frq1] = min(abs(pseudo_freqCWT-p(1)));
        [mini,frq2] = min(abs(pseudo_freqCWT-p(2)));
        [psi2,my2]=a4_tsanas_spindle_detection1_c(...
            CoefsTop10',frq2,frq1,p(3)*new_fs);
```

```
        p(5:15)=p(5:15)*new_fs;
        p(7)=p(7)/new_fs;
        p(10)=p(10)/new_fs;
        [detect]=a4_tsanas_spindle_detection2_c(my2,p(4:6));
        [D]=detection2location(detect);
        if size(D,1)>0
            [detect]=a4_tsanas_spindle_detection3_c(my2,...
                p(7:15),D(:,1)-1,D(:,2)-1,D(:,1));
        end
        detect=a8_moelle_huang_spindle_detection1_c(a_imf,...
            f_imf,detect,new_fs,p0(16:27)); % then apply a6
        % get [heads tails] of detections
        dd=detection2location(detect);
        [SS,T0]=compCroppedSegments(dd,DD,...
            size(CoefsTop10,2),new_fs,stime,seed2);
        ttime=sum(SS(:,2)-SS(:,1))/new_fs/60;
        for j=1:size(SS,1) % cropped segments from C3 signal
            NUM=NUM+1;
            seg_no=seg_no+1;
            d0=SS(j,1);d1=SS(j,2);%head and tail of a segment
            aimfN{NUM}=a_imf(:,d0:d1);
            fimfN{NUM}=f_imf(:,d0:d1);
            coefN{NUM}=CoefsTop10(:,d0:d1)';
            ss=compSpindles(DD,d0,d1);
            DDN{NUM}=ss-d0+1;
        end
    end
    freqM{i}=pseudo_freqCWT;
    % signal length (resampled at 100 Hz)
    lenM(i)=size(CoefsTop10,2);
    segM(i)=seg_no;% number of segments in each data
    fsM(i)=new_fs;% sampling frequency for the i-th data
    disp(['data',num2str(i),' ',num2str(seg_no),...
        ' segments',' ',num2str(ttime,3),' out of ',...
        num2str(T0,3),' minutes']);
end % end of for-i loop
end % % ....... end of a9_tsanas_huang_init_data.m .....

% % ..... beginning of a9_tsanas_huang_train.m .....
function [R]=a9_tsanas_huang_train(p0)
% called by spea2 moea during optimization
global ovlp;% JACCARD overlap coef for true detection grading
global coefN;% store data used in tsanas method
global freqM;% store data used in tsanas method
global aimfN;% store data for segments used in causa method
global fimfN;% store data for segments used in causa method
global DDN;% ground truth [heads,tails] for each segment
global fsM;% sampling frequency for each dataset
global segM;% number of segments in each dataset
global DNUM;% total number of datasets
R = [Inf, Inf];
FP=0;% false positives
FN=0;% false negatives
num=0;
```

```
for i=1:DNUM
    p=p0;
    fs=fsM(i);
    [mini,frq1] = min(abs(freqM{i}-p(1)));
    [mini,frq2] = min(abs(freqM{i}-p(2)));
    p(5:15)=p(5:15)*fs;
    p(7)=p(7)/fs;
    p(10)=p(10)/fs;
    for j=1:segM(i)
        num=num+1;
        [psi2,my2]=a4_tsanas_spindle_detection1_c(...
            coefN{num},frq2,frq1,p(3)*fs);
        [detect]=a4_tsanas_spindle_detection2_c(my2,p(4:6));
        [D]=detection2location(detect);
        if size(D,1)>0
            [detect]=a4_tsanas_spindle_detection3_c(...
                my2,p(7:15),D(:,1)-1,D(:,2)-1,D(:,1));
        end
        detect = a8_moelle_huang_spindle_detection1_c(...
            aimfN{num},fimfN{num},detect,fs,p(16:27));
        [fp,fn,~]=assessByEventJaccard_c(detect,DDN{num},ovlp);
        FN=FN+fn;
        FP=FP+fp;
    end
end
R(1)=FP;
R(2)=FN;
end % % ....... end of a9_tsanas_huang_train.m .....

% % ..... beginning of a9_tsanas_huang_test.m .....
function [P,R,F1]=...
    a9_tsanas_huang_test(database,tests,genes,ovlp,g_std)
% test the parameters in 'genes' for data listed in tests
% refer to 'a0_rain_test_MASS.m' for input arg description
[PN]=compTotalTP(database,tests,g_std);%find total true positives
npar=size(genes,1);% number of parameter sets
FP=zeros(npar,1);% false positives by each parameter
FN=zeros(npar,1);% false negatives by each parameter
for i=1:length(tests)
    disp(tests(i));
    [C3,stage,fs,DD]=get_datai_local(tests(i),g_std,database);
    [rU,rL]=get_rUrLi_local(tests(i),database,C3,fs);
    % preprocessing datasets
    [a_imf,f_imf] = a6_huang_spindle_detection0(C3,rU,rL,fs);
    K=10;% top K cwt coef, signal resampled to 100 Hz
  [CoefsTop10,pseudo_freqCWT,new_fs]=...
      a4_tsanas_spindle_detection0(C3,fs,K);
    DD=floor((DD-1)*new_fs/fs)+1;
    len=size(CoefsTop10,2);
    IX=(1:len)';
    ix=floor((IX-1)*fs/new_fs)+1;
    a_imf=a_imf(:,ix);
    f_imf=f_imf(:,ix);
    for j=1:npar
```

```
            p0=genes(j,:);
            p=p0;
            [mini,frq1] = min(abs(pseudo_freqCWT-p(1)));
            [mini,frq2] = min(abs(pseudo_freqCWT-p(2)));
            [psi2,my2]=a4_tsanas_spindle_detection1_c(...
                CoefsTop10',frq2,frq1,p(3)*new_fs);
            p(5:15)=p(5:15)*new_fs;
            p(7)=p(7)/new_fs;
            p(10)=p(10)/new_fs;
            [detect]=a4_tsanas_spindle_detection2_c(my2,p(4:6));
            [D]=detection2location(detect);
            if size(D,1)>0
                [detect]=a4_tsanas_spindle_detection3_c(...
                    my2,p(7:15),D(:,1)-1,D(:,2)-1,D(:,1));
            end
            detect=a8_moelle_huang_spindle_detection1_c(...
                a_imf,f_imf,detect,new_fs,p0(16:27));%then apply a6
            [fp,fn,tp]=assessByEventJaccard_c(detect,DD,ovlp);
            FN(j)=FN(j)+fn;
            FP(j)=FP(j)+fp;
        end
    end
    TP=PN-FN;% detected true positives
    P=TP./(TP+FP);% precision
    R=TP./(TP+FN);% recall
    F1=2*P.*R./(P+R);% F1-score
    end % % ....... end of a9_tsanas_huang_test.m .....

    % % % % % % % % % % % % % % % % % % % % % % % % % % % % % % % %
    % 9.2 prepare c code for training detector d9
    %     RUN steps 4.1-4.2,6.1-6.3 before proceeding the following
    %
    % % % % % % % % % % % % % % % % % % % % % % % % % % % % % % % %

    % % % % % % % % % % % % % % % % % % % % % % % % % % % % % % % %
    % 9.3 train/test detector d9 with the following command
    %     >> a0_run_example(9,'1',60,1);or a0_run_example(9,'1');
    %     >> a0_run_example(9,'2');
    %     a9_tsanas_huang_run1 was not implemented due to
    %     computational time cost and code complexity
    % % % % % % % % % % % % % % % % % % % % % % % % % % % % % % % %
```

*Published with MATLAB® R2015a*
